# Secreted primary human malignant mesothelioma exosome signature reflects oncogenic cargo

**DOI:** 10.1038/srep32643

**Published:** 2016-09-08

**Authors:** David W. Greening, Hong Ji, Maoshan Chen, Bruce W. S. Robinson, Ian M. Dick, Jenette Creaney, Richard J. Simpson

**Affiliations:** 1 Department of Biochemistry and Genetics, La Trobe Institute for Molecular Science, La Trobe University, Melbourne, Victoria 3086, Australia; 2 National Centre for Asbestos Related Diseases, School of Medicine and Pharmacology, University of Western Australia, 6009, Australia; 3 Department of Respiratory Medicine, Sir Charles Gairdner Hospital, Perth, Western Australia, 6009, Australia; 4 Australian Mesothelioma Tissue Bank, Sir Charles Gairdner Hospital, Perth, Western Australia, 6009, Australia

## Abstract

Malignant mesothelioma (MM) is a highly-aggressive heterogeneous malignancy, typically diagnosed at advanced stage. An important area of mesothelioma biology and progression is understanding intercellular communication and the contribution of the secretome. Exosomes are secreted extracellular vesicles shown to shuttle cellular cargo and direct intercellular communication in the tumour microenvironment, facilitate immunoregulation and metastasis. In this study, quantitative proteomics was used to investigate MM-derived exosomes from distinct human models and identify select cargo protein networks associated with angiogenesis, metastasis, and immunoregulation. Utilising bioinformatics pathway/network analyses, and correlation with previous studies on tumour exosomes, we defined a select mesothelioma exosomal signature (mEXOS, 570 proteins) enriched in tumour antigens and various cancer-specific signalling (HPGD/ENO1/OSMR) and secreted modulators (FN1/ITLN1/MAMDC2/PDGFD/GBP1). Notably, such circulating cargo offers unique insights into mesothelioma progression and tumour microenvironment reprogramming. Functionally, we demonstrate that oncogenic exosomes facilitate the migratory capacity of fibroblast/endothelial cells, supporting the systematic model of MM progression associated with vascular remodelling and angiogenesis. We provide biophysical and proteomic characterisation of exosomes, define a unique oncogenic signature (mEXOS), and demonstrate the regulatory capacity of exosomes in cell migration/tube formation assays. These findings contribute to understanding tumour-stromal crosstalk in the context of MM, and potential new diagnostic and therapeutic extracellular targets.

Malignant mesothelioma (MM) is an incurable malignancy involving serosal tissues, especially the pleura. MM has a median survival from initial diagnosis of 7–9 months^1^. Contributing factors such as the absence of biomarkers and different pathologic subtypes increase the difficulty of treatment, and as a result, individuals with MM generally have a median survival ranging from 11 months with chemotherapy to 7 months with supportive care^2^,^3^. In the next 25 years it is estimated that the diagnosis of MM will increase ~5–10% annually in most industrialized countries at a cost of ~$300 billion worldwide^4^. No single-modality MM therapy including chemotherapy, radiation therapy, immunotherapy, cyto-reductive surgery or surgery has reliably demonstrated superiority to supportive care^5^. Importantly, diagnosis of MM is often difficult and most patients present at an advanced stage. Many blood-based biomarkers for diagnosis of MM have been described, with soluble members of the mesothelin family being the predominant focus^6^,^7^. However, their limited specificity has meant that new tumour-specific markers are being actively sorted^8^,^9^,^10^. Recently, several candidate protein, glycoprotein, antibody, and miRNA markers have been reported^11^,^12^,^13^,^14^,^15^ but still require independent validation. Improved surveillance and early detection of MM using specific markers of initiation and progression are required to improve clinical intervention, and patient survival^16^.

A number of studies in animal models and human patients have demonstrated that inhalation or injection of asbestos fibres results in a chronic inflammatory response characterized primarily by recruitment of cancer-associated fibroblasts (CAFs)^17^ to promote production of chemokines and cytokines in the lung^17^ and pleura^18^. Exposure of human MM cells to asbestos has been shown to facilitate autocrine production and transcriptional regulation of cytokines^19^,^20^. Such findings support a malignant secretory network that can regulate the MM tumour microenvironment and fundamental to understanding the progression of various malignancies, including mesothelioma. Importantly, MM has a highly secretory cell type, and the factors released by cells may act in an autocrine or paracrine fashion on tumour and stroma, where they may modulate the extracellular environment and indeed provide a resource for putative cancer biomarkers^15^. Malignant pleural effusions have been demonstrated to accumulate secreted tumour-derived extracellular vesicles (EVs), specifically exosomes, bearing tumour antigens and antigen-presenting molecules, capable of facilitating anti-tumour immune responses^21^,^22^. Importantly, exosomes from different tumour cells have shown immune activity against not only syngeneic but also allogeneic tumour growth, indicating that tumour-derived exosomes may harbor common tumour antigens capable of inducing antigen-specific immune responses^23^. Therefore, tumour-derived exosomes are a natural and novel source of tumour antigens which could provide alternative diagnostic circulating markers for mesothelioma and its progression but also may represent attractive tumour-specific therapeutic targets^21^,^23^,^24^,^25^.

Exosomes are small (30–150 nm) nano-extracellular vesicles derived from the endosomal pathway by inward budding luminal membranes of multivesicular bodies (MVBs) to form intraluminal vesicles (ILVs); MVBs then traffic to and fuse with the plasma membrane whereupon they release their ILV contents into extracellular space (as exosomes)^26^,^27^. Exosomes have diverse roles in intercellular communication which can be conferred by mediators that are presented on their surface or contained within the lumen. Exosomes contain a specific composition of proteins, lipids, mRNA, regulatory RNA and DNA cargo components^28^. Increasing evidence suggests that exosomes can influence physiological processes such as cell transformation^28^, immunoregulation^25^,^29^, and importantly cancer progression^30^,^31^,^32^,^33^,^34^,^35^,^36^,^37^,^38^, vaccination against infectious disease^39^, and vaccines for possible cancer treatments^40^,^41^,^42^. These studies have led to several clinical and pre-clinical investigations of exosome/EV-based therapies^43^,^44^,^45^,^46^. In the context of therapeutic applications, exosomes of selected cell types have been used as therapeutic agents in immune therapy, vaccination trials, regenerative medicine, and drug delivery^47^. Exosomes also provide an as yet largely untapped source of diagnostic, prognostic, and predictive biomarkers^27^,^29^,^42^. Recently, various innovative therapies involving the *ex vivo* manipulation and subsequent reintroduction of exosome-based therapeutics into humans are being developed and validated, although no exosome-based therapeutics have yet to be brought into the clinic^44^,^47^,^48^.

As a first step towards characterizing the contribution of exosomes in MM, we report in this study an exosomal MM protein signature using four different human MM cell models. Using quantitative proteomic profiling of purified exosomes from each model, we reveal insights into their select oncogenic cargo, demonstrating an enrichment of components associated with immune response, angiogenesis, and cell transformation during cancer progression. Importantly, we demonstrate select proteins known to be associated with cellular migration, invasion, and metastasis enriched in mesothelioma-derived exosomes^49^,^50^,^51^,^52^. Further, we highlight the functional relevance of MM-derived exosomes by pathway analysis associated with immunoregulation. Given that MM progression is associated with vascular remodelling and angiogenesis^53^,^54^, and accumulation of tumour-associated fibroblasts^17^, we investigated the functional effects of oncogenic exosomes from MM on recipient cells in the tumour microenvironment, including fibroblast and endothelial cells. Although studies have shown that many populations of MM contain tumour-associated fibroblasts^55^, little is known about interactions and how tumour-associated fibroblasts are regulated by MM cells. These data support the assertion that selective exosomal cargo may contribute to the progressive changes of mesothelioma by tumour angiogenesis and malignancy. This definitive characterisation and comprehensive informatics analysis of the protein cargo in exosomes derived from different human MM tumours contributes to our understanding of the extracellular environment during MM and identifies potential new diagnostic targets from the extracellular and secreted factors derived from human MM cells.

## Results and Discussion

In this study we have investigated exosomes derived from different human malignant mesothelioma cancer cell models by quantitative protein profiling and functional characterisation to gain insights into secreted modulators of tumourigenicity, immunoregulation, and metastasis.

### Exosomes are released from malignant mesothelioma cells

**Figure 1:**
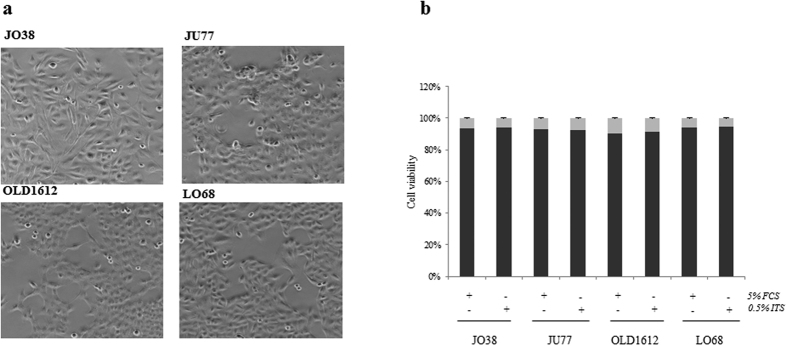
Human malignant mesothelioma cell characterisation. (**a**) Phase contrast images of human MM cells JO38, JU77, OLD1612, and LO68 reveal elongated fibroblast-like morphology. (**b**) Cell viability as determined by the Trypan Blue dye exclusion assay after culture in presence of 5% FCS (foetal calf serum) or 0.5% ITS (insulin-transferrin-selenium) conditions for 24 hr, demonstrating that all cell lines remain >92% viable. Data representative of mean ± SEM of three independent experiments performed in triplicate.

**Figure 2:**
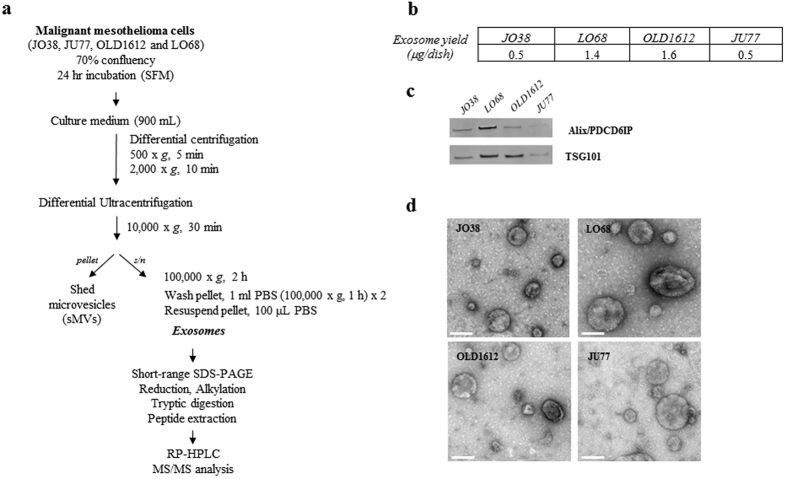
Isolation and characterisation of MM-derived exosomes. (**a**) Workflow showing isolation and purification of exosomes from mesothelioma cell lines using serum-free media (SFM) conditions. Exosomes (10 μg) were solubilised in SDS, separated by 1D-SDS-PAGE and fractions (n = 2) subjected to in-gel reduction, alkylation, and tryptic digestion. Extracted peptides were fractionated and identified using mass spectrometry analysis, data processing database searching, informatics and protein annotation. (**b**) Protein yield (μg/cell dish) for JO38, JU77, OLD1612, and LO68 exosomes is shown (average n = 3). (**c**) For Western blotting, exosome preparations (10 μg) were separated by 1D-SDS-PAGE, electrotransferred, and probed with exosome markers Alix/PDCD6IP and TSG101. Data representative of three independent experiments. (**d**) For transmission electron microscopy, exosomes (2 μg) were negatively stained using uranyl acetate and viewed by transmission EM, revealing a relatively homogenous population of round membranous vesicles 30–150 nm in size for all cell types. Scale bar, 100 nm. Representative image from n = 3 and 5 independent fields of view.

Human MM cells (JO38, JU77, OLD1612, LO68) displayed a fibroblast-like mesenchymal phenotype in culture (Fig. 1a). Exosomes were isolated from cell conditioned medium in serum-free conditions for 24 hr (>92% cell viability) (Fig. 1b). Exosome isolation and enrichment strategy is shown (Fig. 2a). Exosome yield was in range 0.5–1.6 μg protein/dish (mean ~90 μg/~8 × 10^8^ cells) (Fig. 2b). Western blot analysis confirmed expression of exosome markers Alix/PDCD6IP and TSG101 (Fig. 2c), and transmission electron microscopy revealed a relatively homogenous population of spherical membranous vesicles 30–150 nm in diameter (Fig. 2d), which is in accordance with the typical size reported for exosomes^56^,^57^.

### Proteome analysis of human MM-derived exosomes

**Figure 3:**
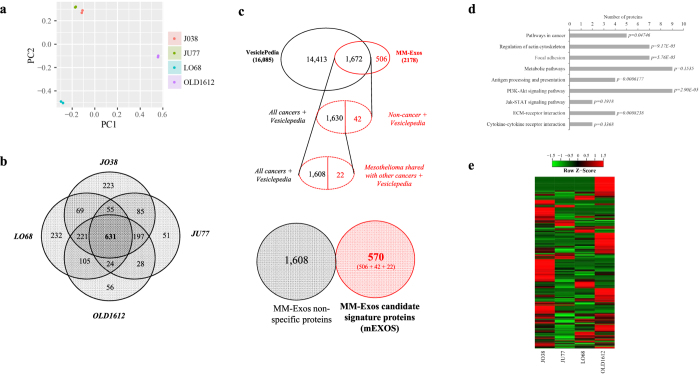
Characterisation of mesothelioma-derived exosomes reveals an exosomal-specific signature (mEXOS). (**a**) Principal component analysis (PCA). Each symbol represents a biological replicate, and the colour represents the group (model). (**b**) A four-way Venn diagram of proteins distributed between each MM-derived exosome type is shown, revealing 631 proteins common to each dataset. (**c**) To determine the classification of proteins in mEXOS we applied a stringent filtering criteria. The total MM-derived exosomal proteins (2,178) were compared with the Vesiclepedia database (comprising 16,085 human entries) and literature searching, of which 506 were unique to this study and not previously reported in the context of extracellular vesicles. Among the 1,672 co-identified proteins, 42 were non-cancer proteins reported in Vesiclepedia. with a further 22 proteins reported shared with mesothelioma and other cancers in Vesiclepedia. Therefore, to determine the unique MM exosome protein signature (mEXOS), we summated the 506, 42, and 22 proteins from these categories to reveal 570 proteins as select exosomal and MM-derived components (Table S3). (**d**) KEGG pathway analysis of mEXOS, with p-values indicated. (**e**) Correlation matrix of mEXOS, representing differential abundance based on normalised spectral count (SpC) values between each of the MM models investigated, showing that each individual sample represents clear distribution and similarity with other MM models.

Quantitative proteomic profiling identified a total of 2,178 proteins in exosomes from different human-derived MM cells (biological duplicate) (Supplementary Dataset). We evaluated the spatial separation for these proteins in the studied groups using principal component analysis (PCA). The result of PCA demonstrated good separation between MM models (Fig. 3a). Distribution of identified proteins between each MM-derived exosome type is shown, revealing a common subset of 631 exosomal proteins identified between all models (Fig. 3b). The 631 common exosomal proteins included proteins involved in exosome biogenesis (including TSG101, VPS26A, VPS29, VPS35, and Alix^58^) and intracellular vesicle trafficking (including tetraspanins CD63 and CD9, CD81/CD82, and small Rab GTPases^59^,^60^,^61^) (Table S1). The majority of the common mesothelioma exosome proteins (508/631 (80%)) have previously been observed in exosomes released from diverse cell types (see EV database compendium Vesiclepedia containing 93,980 protein/mRNA entries (16,085 human proteins/mRNAs)^62^). Similarly, several key proteins associated with exosome biogenesis (ESCRT-associated, tetraspanins), sorting/trafficking (including Rab GTPases, ADP-ribosylation factors, clathrin and coatomer subunits, lipid raft flotillins), and vesicle release (including synaptotagmin 1/2, dynamin 1/2, synaptogyrin, VAMP3 and VAT1) (Table S2) were identified, though were not common to all cells. Nearly a quarter of the total number of exosomal proteins identified from JO38, JU77, OLD1612, and LO68 cells (506/2,178 (23%)) have not been previously reported in the EV database, Vesiclepedia (Supplementary Dataset). Besides common proteins associated with vesicle biogenesis and trafficking and the fact that only a single other proteomic study associated with mesothelioma and EVs is reported in Vesiclepedia^63^, this indicates the selectivity of unique oncogenic cargo within MM-derived exosomes, in addition to improvements in proteomics technologies and mass spectrometry.

In comparison to exosomes derived from human mesothelioma models, Hegmans *et al*.^63^ reported the proteomic characterisation of exosomes derived from two different human mesothelioma cells (PMR-MM7 and PMRMM8) by MALDI-TOF mass spectrometry. The study identified 19 proteins, with components associated with antigen presentation, including MHC class I molecules and Hsp90, although no mesothelioma-associated antigens were identified. In comparison, we report all these proteins in mesothelioma-derived exosomes, and importantly 15 proteins were in the common 631 exosomal protein list (Table S3). Further, Clayton *et al*.^64^ detected Her2-neu, mesothelin, CD9, CD81, LAMP-1 and MHC-like MICA by Western blotting in tumour-derived exosomes from advanced pleural MM. In comparison, this study identified mesothelin, CD9, and CD81 in exosomes, with CD9 and CD81 being common to all mesothelioma-derived exosomes. Clayton *et al*.^65^ further demonstrated a direct interaction between the activating receptor natural killer group 2D (NKG2D) receptor (MHC class-I related) on exosomes from various cancer cell models/primary cells and natural killer (NK) cells or CD8^+^ T cells. This interaction was shown to implicate NKG2D as a target for exosome-mediated tumour immune evasion. Primary mesothelioma cell-derived exosomes were shown to harbour typical exosome markers including MHC class I and TSG101. Expression of MHC-like molecules MICA/MICB were also demonstrated. Interestingly, we report such proteins in MM-derived exosomes (OLD1612 cells). More recently, Manfredi *et al*.^15^ have investigated the secretome using proteomic profiling from different human MM models to reveal proteins commonly expressed between these studies (i.e., MM98: 78/208 common, REN: 46/112). Of note, we report 2,073 exosomal proteins unique to our study. Therefore, this current study on proteomic profiling of MM-derived exosomes provides an extensive insight into the protein cargo of cancer exosomes, direct comparison with previous reports on MM-derived exosomes and the secretome, and significantly increased coverage of exosomal-associated proteins not previously reported in Vesiclepedia or identified from human MM models.

### Mesothelioma-enriched exosome protein cancer signature (mEXOS)

To address the contribution of exosome cargo proteins to mesothelioma in comparison to other cancer types (i.e., mesothelioma-enriched exosome protein cancer signature, mEXOS) we used a stringent filtering criteria utilising a combination of Vesiclepedia, and literature searching of exosomal studies investigating mesothelioma cells/pleural effusion (Fig. 3c) 21,23,63,64,65. Of the 2,178 proteins identified in this study, 1,672 were common to various cancer-types reported in Vesiclepedia (i.e., bladder, breast, colorectal, melanoma, ovarian, prostate, stomach cancers), while the remaining 506 proteins were categorised as unique to MM only. These unique exosomal proteins represent a select and novel dataset for the comprehensive analysis of MM and exosomal proteins. Among the 1,672 proteins, 42 were non-cancer proteins reported in Vesiclepedia, with a further 22 proteins shared by mesothelioma and other cancers in Vesiclepedia (Fig. 3c). In total, we report 570 proteins in defining a unique MM exosome protein signature (mEXOS) containing new candidate biomarkers for MM (Fig. 3c) (Table S4). Interestingly, 142/570 proteins were identified in all MM-derived exosomes. For proteins classified into mEXOS, KEGG pathway analysis and Gene Ontology were used to determine pathways enriched, and protein information on subcellular location, biological function, and cellular component (Figure S1). Of note we observe KEGG enrichment (Fig. 3d) associated with “pathways in cancer” (ko05200), “cytokine receptor interaction” (ko04060), “regulation of actin cytoskeleton” (ko04810), “focal adhesion” (ko04510), “metabolic pathways” (ko01100), “antigen processing and presentation” (ko04612), “extracellular matrix-receptor interaction” (ko04512), “PI3K-Akt signalling pathway” (ko04151) and “Jak-STAT signalling pathway” (ko04630).

Several select protein networks were identified in mEXOS, demonstrating the variability in protein expression between distinct human MM models (Fig. 3a,e). This cluster and normalised heat map analysis revealed that MM-derived exosomes from JU77 and JO38 were most similar in expression profiles, while similarities were identified between LO68 and OLD1612 models. These protein expression cluster analyses align the cell models with the amount of exosomes released from malignant mesothelioma cells, with JU77 and JO38 (low yield producing) and LO68 and OLD1612 (high yield producing) (Figs 2b and 3a). There were several exosomal cargo proteins whose expression and abundance profile were similar across all models, including villin 2 (VIL2), transferrin receptor (TFRC), thrombospondin 1 fragment (THBS1), and lactadherin (MFGE8). Interestingly, such proteins have been shown to be involved in cell-to-cell and cell-to-matrix interactions^66^,^67^,^68^,^69^,^70^,^71^, and as for MFGE8, an important role in the maintenance of intestinal epithelial homeostasis, promotion of mucosal healing, and neovascularization^72^,^73^. A salient finding has been the recent implication of MFGE8 in modulating the tumour microenvironment and promote tumourigenicity in primary lung cancer cells^74^. Such MM-specific information provide an integrative analysis of the selective extracellular cargo components associated with different human-derived MM models *in vitro* and importantly new candidate extracellular biomarkers for MM.

### Exploration of candidate exosomal biomarkers for MM

We report several select MM specific and abundant markers in mEXOS which have direct clinical relevance and correlation with previously identified expression profiling studies investigating MM (Table S4). We report 5 known tubulin isotypes in mEXOS, including proteins TUBB4A (470 SpC), Q8IWP6 (class IVb beta tubulin) (544 SpC), and B3KPS3 (455 SpC). Emerging evidence suggests that tubulins and the tubulin-microtuble network are critically involved in cell stress responses involved in cancer^75^. Tubulins are now considered important targets for chemotherapeutic drugs in many solid tumour malignancies including mesothelioma^76^,^77^. Further, we report galectin-3-binding protein (LG3BP/B4DVE1) (465 SpC) in mEXOS, and importantly, the first report of LG3BP in MM-derived exosomes. As an important mediator of integrin cell adhesion, expression of LG3BP/B4DVE1 has been shown to be significantly up-regulated in malignant pleural mesothelioma^78^ and it has been reported in other tumour-derived exosomes including colorectal, breast, and bladder^79^.

Further potential markers within mEXOS include alpha-enolase (ENO1), annexin A1 (ANXA1), and glucose-6-phosphate 1-dehydrogenase (A8K8D9/G6PD). ENO1 (307 SpC) is a multifunctional enzyme that, as well as its role in glycolysis, plays a part in various processes such as cell control, hypoxia, and the innate immune response^80^. Further, ENO1 is suggested to function in the intravascular and pericellular fibrinolytic system due to its ability to serve as a receptor and activator of plasminogen on the cell surface of several cell-types such as leukocytes and neurons, and further stimulates immunoglobulin production^81^. Of note, ENO1 has previously been identified in mesothelioma exosomes^63^. ANX1 (102 SpC) is known to contribute to the adaptive immune response by enhancing signalling cascades that are triggered by T-cell activation, regulates differentiation and proliferation of activated T-cells, inflammation and wound healing, cell polarization, and cell migration^82^. ANX1 has previously been shown to be important in the response to oxidative stress in mesothelioma cells^83^. Moreover, G6PD (190 SpC) is necessary for oxidative ribose production, controlling the pentose phosphate pathway^84^. Importantly, G6PD has been revealed to be involved in apoptosis, angiogenesis, and the efficacy to anti-cancer therapy, making it a promising target in directing and monitoring cancer therapy^85^. High levels of G6PD are observed in some cancers and its expression can transform fibroblasts to induce tumour formation *in vivo*^86^. These proteins were identified across all MM exosomes, and provide potential common markers that define a MM exosome-related signature. The fact that these proteins can be identified in exosomes is an exciting indication that identification and production of MM factors may be used as biomarkers of tumour development or as indicators of patient responses to therapeutic regimens or surgical resection of MM.

In the context of previous studies investigating novel tumour-derived biomarkers for MM, Suraokar *et al*.^87^ reported gene expression microarray profiling (global transcriptomic microarray analysis) of 53 surgically resected MMs tumours along with paired normal tissue. In comparison to mEXOS, we report 9 of their gene products (BCAT1, CALB2, CCDC68, CFB, HPGD, LAMA1, MAMDC2, MAP2 and SULF1), with 5 genes upregulated in expression correlating with MMs tumours (BCAT1, CALB2, CFB, LAMA1, and SULF1). Crispi *et al*.^88^ performed Affymetrix HGU133A plus 2.0 microarray analysis to molecularly dissect mesothelioma tumour pathways and identify new tumour biomarkers that could be used as early diagnostic markers and possibly as specific molecular therapeutic targets. In comparison, we report 3 proteins (ANXA6, LAMA1 and NRP2) in mEXOS. Annexin A6 (ANXA6) has been identified in exosomes derived from human mesothelioma cells^63^ and shown to regulate membrane-cytoskeleton dynamics and membrane-fusion events between intracellular compartments, and further play a role in the inward vesiculation process^89^. Neuropilins (NRPs) are multifunctional non–tyrosine kinase receptors, expressed at the surface of endothelial cells, with NRP2 expressed in the lymphatic system^90^. The expression of NRP2 contributes to tumour-angiogenesis and lymphangiogenesis^91^, and importantly contribute to cell communication processes, influencing cell positioning and behaviour, and tissue morphogenesis^92^. In comparison to additional known factors identified in mEXOS and associated with MM, we report cyclooxygenase-2 (COX-2) and calretinin (CALB2). COX2 is implicated in many events in the tumorigenic process, producing highly reactive products that can affect cell growth, immune response, apoptosis, and angioneogenesis^93^. Importantly, high COX-2 expression is a marker of poor prognosis in MM^94^. CALB2 is a vitamin D–dependent calcium-binding protein involved in calcium signalling, and recently shown to be an important sensitive and specific diagnostic marker for MM in serous effusions^95^. CALB2 is an established immunohistochemical marker used in the diagnosis of mesothelioma^96^ and frequently used for the diagnosis of MM (as apposed to lung adenocarcinoma)^97^. It has further been reported as a blood-based marker of mesothelioma^98^. Interestingly, CALB2 has previously been found in exosomes from malignant pleural effusion^99^.

**Figure 4:**
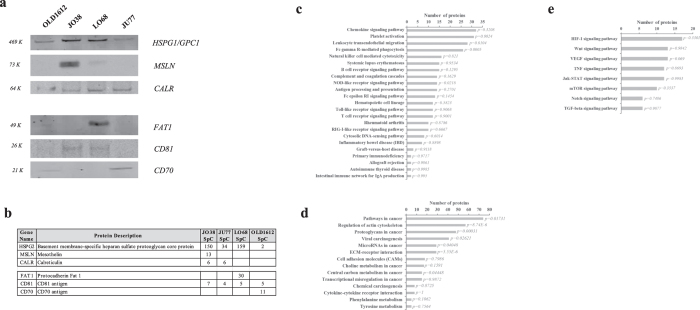
Validation and pathway analysis of mesothelioma-derived exosome cargo. (**a**) To validate the relative abundance of proteins identified using GeLC-MS/MS, Western blot analysis was used to compare expression for selected proteins; glypican-1 (HSPG1/GPC1), mesothelin (MSLN), calreticulin (CALR), and other exosome components protocadherin Fat 1 (FAT1), CD81, and CD70. For Western blotting, exosomes (10 μg) were separated by 1D-SDS-PAGE, electrotransferred, and probed with markers as indicated (n = 2). (**b**) Relative quantitation of label-free spectral counting (SpC) are shown for selected proteins validated using Western immunoblotting (n = 3). (**c**) Immune system and immune disease related pathways in MM-derived exosomes, with p-values indicated. (**d**) Cancer cell biology related pathways in MM-derived exosomes, with p-values indicated. (**e**) Signal transduction related pathways in MM-derived exosomes, with p-values indicated.

Additionally, we report several key proteins in MM-derived exosome cargo (although not specific to mEXOS) known to be expressed in mesothelioma, including mesothelin (MSLN), calreticulin (CALR), RuvB-like proteins (RUVBL1/2), proteins signal transducer and activator of transcription 1 (STAT1), vimentin (VIM), and superoxide dismutase (SOD) 1/2 (Table 1) (Fig. 4a,b)^100^. MSLN is an established marker in mesothelioma diagnosis^6^,^96^. MSLN has been identified enriched in tumour-derived exosomes^21^,^23^,^101^ and exosomes from malignant pleural effusion^99^. The RUVBL1/2 proteins are involved in chromatin remodelling^102^, DNA interaction and repair^103^, and promote interaction of the modified histones with other proteins which positively regulate transcription^104^. These proteins may be required for the activation of transcriptional programs associated with oncogene and proto-oncogene-mediated growth induction, and DNA repair. There has been extensive work on several DNA-inducible genes as human MM biomarkers of exposure to these agents, including p53 induction of DNA strand breaks, p53 expression, and apoptosis in cell lines, particularly in cultured mesothelial cells^105^. Such *in vivo* findings highlight the importance of oxidative damage in asbestos-induced carcinogenesis, however were unable to define the specific differentially expressed components and molecular basis of asbestos-induced disease and MM progression. Further, STAT1 which is known to have an oncogenic function and is constitutively activated in many human cancer cells including human MM^106^,^107^, has not previously been observed in mesothelioma, or lung cancer exosomes. The proteins identified in this profiling study, and specifically the oncogenic signature (mEXOS), represents an extensive and important catalogue of proteins attributed in exosomes specifically in the context of human MM and may represent new selective extracellular and circulating targets in MM progression, diagnosis, and monitoring.

### Mesothelioma-derived exosomes contain immunoregulatory components

Tumour-derived exosomes have been shown to be immunomodulatory during cancer progression^25^. In this study, KEGG pathway annotation for the 2,178 proteins revealed pathways related to immune system and immune diseases (Fig. 4c). For these data, we identified a total of 111 proteins associated with immunoregulation in MM-derived exosomes (Table S5), of which 26 were identified in mEXOS, including oncostatin-M receptor (OSMR), multidrug resistance-associated protein 1 (ABCC1), and the SUMO-1 activating receptor, SAE1. OSMR is a multifunctional cell surface cytokine receptor, which induces several pro-malignant effects, including a pro-angiogenic phenotype and increased cell migration and invasiveness^108^. Recently, OSMR mRNA has been shown to be significantly elevated in malignant pleural mesothelioma, compared with benign asbestos-related pleural effusion^109^. Further, OSMR which is a multifunctional cell surface cytokine, previously found to be over-expressed in mesothelioma, has been suggested to be a candidate for antibody-mediated targeted inhibition^110^. Expression of the ATP-binding cassette superfamily member ABCC1 has been previously demonstrated in non-small-cell lung carcinoma^111^ and is an important immunoregulatory component of chemotherapy resistance^112^. The CD70 antigen is expressed by limited subsets of normal lymphocytes and dendritic cells (DCs), but aberrantly expressed by a broad range of hematologic malignancies and some solid tumours^113^,^114^,^115^. CD70 is associated with MHC class II where it acts as a co-stimulatory molecule, with identification of CD70 previously associated with exosomes in contributing to their immunostimulatory capacity^116^,^117^.

Of the immune-associated components, 30 different proteins were identified associated with MHC I antigen processing and presentation (e.g., TCP-1-beta, protein-methionine sulfoxide oxidase MICAL1, and plastin-3), and 21 proteins identified associated with MHC II antigen processing and presentation (e.g., ACLY variant protein, copine-1, and guanine nucleotide-binding protein subunit beta-2-like 1). Furthermore, various proteins were identified associated with the B-cell response (e.g., vitronectin, and toll interacting protein), NK-cell response (e.g., SUMO-1 activating enzyme, and long-chain-fatty-acid-CoA ligase 4), T-cell response (e.g., tensin-3, and TELO2-interacting protein 1), and other related components associated with the immune response (e.g., STAT1, MX1, and MX2). Further, 28 proteins (24 associated with B-cell response, 4 associated with T-cell response) have been identified in previous studies associated with EVs^117^,^118^,^119^.

Interestingly, we observed exosomal protein components co-identified in a previous immune signature of malignant pleural mesothelioma based on clustering of relevant enriched genes to mesothelioma (tissue, cell lines) compared to non-malignant mesothelial cells^120^. In comparison, this current study identified 9 components in exosomes derived from MM, including the interferon-induced proteins IFIT1, MX1/2, and STAT1. EVs have previously been shown to mediate the transfer and specific activation of STAT1 and pro-inflammatory cytokine signalling in target cells^121^. This current study represents a significant subset of proteins associated with immunoregulation, not previously associated with exosomes or EVs, or attributed to MM (Table S5). Collectively, we highlight the importance of MM-derived exosomes revealed by pathway analysis and bioinformatics analysis associated with immunoregulation Given the chronic inflammatory response characteristic of MM progression, it remains to be determined whether such components are functionally active in tumour-derived exosomes, or in target recipient cells associated with the tumour microenvironment.

### Exosomes as carriers of tumour-derived antigens

Immune responses have been shown to be beneficial in MM patients^122^,^123^, therefore, focusing immunotherapeutic strategies on promoting these immune responses is an attractive approach. Towards this aim, tumour- and immune cell-derived exosomes have been shown to carry tumour antigens and modulate the immune response, leading to eradication of established tumours by CD8+ T cells and CD4+ T cells, as well as directly suppressing tumour growth and resistance to malignant tumour development^24^,^25^,^124^. Therefore, in this study, to investigate the presence of tumour-specific antigens within exosomes in the context of MM, we utilised a combination of bioinformatics and gene ontology analyses to reveal 16 select tumour-derived antigens as cargo components in MM-derived exosomes, including CD70 antigen, cleavage and polyadenylation specificity factor subunit 1 and 3 (CPSF1/3), melanoma-associated antigen D2 (11B6), glypican-1, BJ-HCC-24 tumour antigen, and mesothelin (CAK1 antigen) (Table 2). Several candidates were validated using immunoblotting (Fig. 4a) and correlated with mass spectrometry expression (Fig. 4b). Tumour proteins (including mesothelin) derived from patients with mesothelioma have been shown to induce a spontaneous humoral immune response^125^,^126^. Such responses against tumour-derived antigens include antibodies against these antigens, and may be useful as diagnostic tumour markers and targets for immune-based therapies^127^,^128^,^129^. Previously, exosomes are known to transfer tumour antigens, including mesothelin, to DCs for antigen presentation and cross-presentation^21^,^23^,^130^. The tumour-associated antigen MAGE proteins have attracted considerable interest in vaccine-based cancer immunotherapies^131^,^132^. MAGE-D2 has recently been shown to suppress expression of the apoptotic death receptor 2 (TRAIL-R2), promoting and protecting melanoma cells from apoptosis^133^. Several annexins (ANXA1-6, 11) were further identified across various MM-derived exosomes. Annexins have been implicated in several functions including membrane trafficking, cell signalling, ion transport, inflammation, apoptosis and haemostasis. Previously, ANXA2 has been reported to be a specific and selective antigen overexpressed in lung cancer tissues with high asbestos exposure and capable of inducing humoral immunity in MM patients^134^. Recently, ANXA2 has been identified as an antigenic target for pancreatic cancer immunotherapy^135^.

In addition to tumour-associated antigens, we further report several proteins attributed to overexpression or accumulation in human tumours and biofluids, including melanotransferrin (melanoma-associated antigen p97), protocadherin Fat 1 (FAT1), fibroblast growth factor-binding protein 1, and mucins 5AC/5B/13^136^,^137^,^138^ (Table 2). Several of these proteins were validated using immunoblotting (Fig. 4a) and correlated with mass spectrometry results (Fig. 4b). Interestingly, antibodies against tumour antigens on mucins are widely used clinically as diagnostic tools (serum assays) for different cancer types, including colorectal, breast, ovarian and pancreatic adenocarcinoma^138^. Exosomes have been shown to mediate the transfer of modified tumour antigens, including mucins, to generate an immune response against tumours, highlighting the role of such vesicles as carriers of exogenous tumour antigens and in immunoregulation^130^.

### Cancer signalling networks reflected in mesothelioma-derived exosome protein cargo

To identify pathways associated with cancer signalling in MM exosomes, we performed KEGG pathway annotation for the 2,178 proteins to reveal pathways related to cancer cell biology (cell motility, ECM-receptor interaction) (Fig. 4d), and signal transduction (including Jak-STAT signalling, TGF-β, TNF, mTOR, Wnt, VEGF, and Notch pathways (Fig. 4e). With relevance to Jak-STAT signalling, exosomes have been attributed to IFN-α-induced cell-to-cell transfer of antiviral components from LNPCs to HBV-infected hepatocytes both *in vitro* and *in vivo*^139^. The correlation of activation of Jak-STAT pathway with TGF-β expression in mesothelioma-derived exosomes has been previously associated with anti-proliferation^64^ and implicated in progression of MM^107^,^140^. TGF-β signalling has been attributed to transcription regulation of connective tissue growth factor (CTGF), enhancing expression of CTGF and directly controlling pro-oncogenic effects (cell proliferation, extracellular matrix remodelling) in MM^140^. Expression of STAT1/3/5 have been shown dysregulated in human MM (up-regulation of STAT1/5, down-regulation of STAT3), associated with downstream EGFR signalling, and inversely correlate with patient survival^107^. Further, selective MEK or PI3K kinase inhibitors are equally effective in down-regulating the pro-metastatic phenotype, suggesting that MEK or PI3K are appropriate targets for development of molecular therapeutics for MM^141^. Tumour-derived exosomes from pleural effusions of mesothelioma patients have been shown to partially modulate recipient immune cell function, through TGF-β signalling^65^. Associated with cancer cell signalling and various pathways related to cancer cell biology, we report heparan sulfate proteoglycan glypican-1 (HSPG1/GPC1) in exosomes derived from all MM models in this study. Previously, exosomal GPC1 has been shown to modulate the angiogenic and metastatic potential of human and mouse cancer cells^142^. GPC1 has been attributed to melanoma development and progression^143^, and increased expression in human glioma tumours and glioma-derived cell lines^144^. Further, GPC1 has recently been identified to be specifically enriched on cancer-cell-derived exosomes^37^. Tumour-derived GPC1-positive exosomes were capable of specifically distinguishing healthy subjects and patients with a benign pancreatic disease from patients with early- and late-stage pancreatic cancer, and correlate with tumour burden and survival of pre- and post-surgical patients with pancreatic cancer (carcinoma *in situ*, stage I and stages II–IV)^37^, supporting its utility as a biomarker for all stages of pancreatic cancer and its potential for early detection. As discussed, we highlight the importance of MM-derived exosomal cargo revealed by pathway and network analysis and bioinformatics analysis associated with immunoregulation. These findings may suggest that MICA (or other such related molecules), as cargo components of tumour exosomes, may facilitate targeting tumour exosomes to defined immune cells including CD8+ T cells and NK cells, to deliver ligands in a cell-type selective manner. Interestingly, we report both the identification of MICA-L1 and –L3, and significant elevated expression of components associated with TGF-β signalling in mesothelioma-derived exosomes. It remains unknown of the mechanisms of how tumour-derived exosomes selectively target and transfer ligands to specific immune cells and recipient cells associated with the tumour microenvironment to modulate the immunogenic response.

### Mesothelioma exosomes regulate recipient cells of the tumour microenvironment

**Figure 5:**
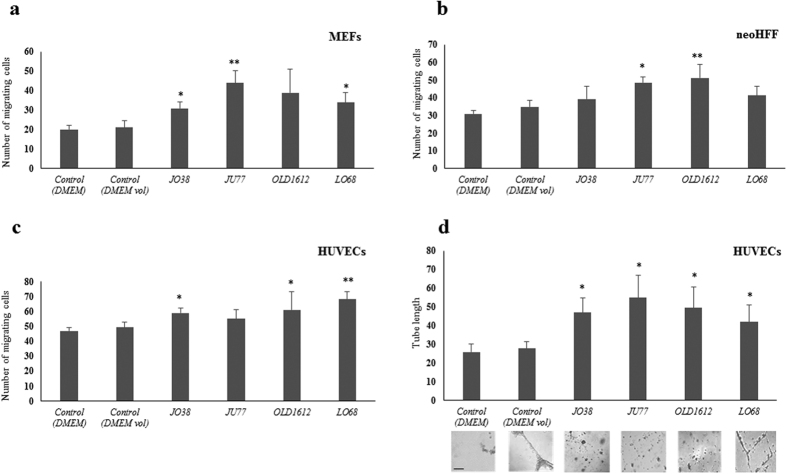
Mesothelioma exosomes regulate recipient cells of the tumour microenvironment. To explore the potential exosome-mediated regulation of cells in the tumour microenvironment, fibroblasts (MEFs, neoHFFs) and endothelial cells (HUVECs) were exposed to MM cell-derived exosomes and recipient cell function analysed. For recipient cell migration, MEF (**a)**, neoHFF (**b**) and HUVEC (**c**) cells were investigated using transwell assays over 24 hr in response to exosomes (30 μg/mL) derived from JO38, JU77, OLD1612, and LO68 cells. Vehicle control (DMEM), in addition to exosome-free control (exosome-depleted) were used. Transversing cells were stained with DAPI, imaged, and counted (n = 3; average ± sem; *p < 0.05, **p < 0.01). Tube formation assays using HUVEC cells (7 × 10^4^), supplemented with MM-derived exosomes (30 μg/mL) and controls and seeded onto Matrigel (1 mg/mL). After 24 hr, tube formation was analysed, imaged, and quantified. Scale bar = 50 μm. (representative images from *n* = 3, average ± sem; *p < 0.05).

To demonstrate that MM-derived exosomes transport functionally active cargo, exosomes were investigated for their ability to regulate fibroblast and endothelial cells within the tumour microenvironment. Various mouse and human fibroblast models (MEFs/neoHFFs) and human endothelial cells (HUVECs) were cultured in DMEM-supplemented with MM exosomes (30 μg/mL), and over a 24 hr period, displayed significantly higher migration rates compared with vehicle controls alone (Fig. 5a–c). Recipient fibroblast cell migration was monitored using the transwell assay, which demonstrated that MM exosomes (30 μg/mL) significantly increased MEF cell migration for exosomes derived from JO38, OLD1612, and LO68 cells compared to vehicle treated cells (Fig. 5a), and exosomes derived from JU77 and OLD1612 significantly increased neoHFF cell migration (Fig. 5b). Further, exosomes from JO38, OLD1612, and LO68 cells resulted in significantly increased endothelial cell migration of HUVECs (Fig. 5c). Further, supplementation with MM-derived exosomes resulted in significantly increased HUVEC tube length formation compared to vehicle (control) treated cells (Fig. 5d). Together, these data demonstrate that exosomes derived from MM cells can differentially promote migratory capacity in recipient fibroblast and endothelial cells and HUVEC angiogenesis.

Previously, tumour exosomes (30 μg/mL) derived from mammary epithelial BT-474 cells have been shown to significantly increase cell proliferation of parental BT-474 cells^145^. Further, gastric cancer SGC7901-cell-derived exosomes have been shown to promote activation of PI3K/Akt and proliferation of SGC7901 and BGC823 cells^146^. Interestingly, proteomic analysis has revealed that abundant cell migratory and angiogenic factors are present in MM-derived exosomes^147^. Exosome uptake was shown to induce upregulation of angiogenesis-related genes and result in enhanced endothelial cell proliferation, migration, and sprouting^148^. Therefore, MM- and tumour-derived exosomes can directly facilitate reprogramming of recipient cells of the tumour microenvironment to facilitate cell migration and angiogenesis.

### Mesothelioma-derived exosomes contain factors associated with metastasis

Tumour-derived exosomes have been shown to modulate the metastatic niche^30^,^149^,^150^,^151^. Importantly, various changes which facilitate metastasis have been described in the lung. These pre-formed lung niches encompass cells recruited from the bone marrow and resident cells such as club/Clara cells^152^ and alveolar macrophages^153^. Factors produced by resident cells in metastatic niches can enhance the survival and outgrowth of disseminated tumour cells. To gain insights into specific components from mesothelioma-derived exosomes that may reprogram the lung microenvironment and promote metastatic formation, we compared our data with several key studies investigating factors associated with the metastatic niche, including the lung^30^,^150^,^151^,^154^,^155^,^156^,^157^,^158^,^159^. Several mediators of tissue invasion, intravasation and metastasis were identified including growth factor receptors, oncoproteins, proteases, and chemoattractants (Table 3). Macrophage migration inhibitory factor (MIF) was expressed in all mesothelioma-derived exosomes, and interestingly has been reported critical in pancreatic exosomes in regulating liver pre-metastatic niche formation and metastasis^30^. Further, compared with patients whose pancreatic tumours did not progress, MIF expression in exosomes was markedly higher from stage I pancreatic cancer patients who later developed liver metastasis^30^. Recent studies indicate that exosomes contain fibronectin on their external surface which facilitate interaction with target cells through heparin sulfate^160^. As the accumulation of fibronectin (FN1) in the metastatic niche is one of the earliest stages of metastasis formation, exosomes are now considered an early and fundamental driver of microenvironment reprogramming. In fact, in this study we observed FN1 in mEXOS and for MM models JU77, LO68, and OLD1612 (Table S4). Interestingly, we note the significant abundance of peptides identified for this protein between these models, indicating the significant expression of FN1 cargo in these exosomes.

Tenascin C (TNC) is associated with extracellular matrix remodelling and deposition and formation of the metastatic niche. TNC has recently been shown from primary breast cancer cells to colonize lung metastatic niche formation^155^,^161^. TNC is associated with development and progression of pulmonary micrometastases^161^, and suggested to promote tumour cell dissemination and survival during metastasis by growth factor binding, and interactions with extracellular matrix components including fibronectin, proteoglycans, fibrinogen, matrix metalloproteinases, and cell surface receptors, EGFR and integrins^162^. Melanoma-derived exosomes have been demonstrated, through a MET signalling-dependent pathway, to promote the metastatic process *in vivo*^32^. Upon activation, various cytoplasmic effector molecules including growth factor receptor-bound protein 2 (GRB2), and SRC are further recruited to the MET receptor^163^. In this study, we report the identification of MET, GRB2 and SRC in mesothelioma-derived exosomes. Previously, in comparison of human metastatic and primary colorectal cancer cell-derived exosomes, we reported the identification and significant up-regulation of MET, GRB2, and SRC^49^. The selective enrichment of metastatic factors in MM-derived exosomes contributes to our understanding of the crosstalk between tumour and stromal cells in the tumour microenvironment in the context of local disease progression. Understanding the functional role of the secretome^15^ – specifically exosome components - in this lung-specific environment remains to be further investigated in the context of MM.

In addition, we report several proteases implicated in the metastatic niche, including matrix metalloproteinases MMP-2, MMP-14, a disintegrin and metalloproteinase 10 (ADAM10), and ADAM with thrombospondin motif 1 (ADAMTS1). Secretion of matrix-degrading enzymes, including MMP-2 and -14 play an essential role in oncogenic cell transformation and subsequent tumour migration/invasion, serving to mediate the breakdown of basement membrane barriers^164^. Primary tumour-derived exosomes have also been demonstrated to mediate changes in expression of MMP-2, and MMP-9 in the metastatic niche^149^,^159^. Further, the chemoattractant S100 calcium binding proteins S100A6/A10/A11 were identified in all mesothelioma cell-derived exosomes. S100 proteins are commonly up-regulated in tumours and typically associated with tumour progression, including metastasis^165^. Of interest, S100A10 has been attributed to tumour growth and invasion, and a key component in metastatic evolution through recruitment of tumour-associated macrophages to the tumour microenvironment, mediating inflammation, angiogenesis, suppressing antitumor immunity, and matrix remodelling^166^. Further, S100A11 is associated with tumour lymph node metastasis in metastatic non-small cell lung cancer and metastatic hepatocellular carcinoma^167^, and pancreatic adenocarcinoma^165^. Recently, secreted S100A11 in normal human keratinocytes was shown to promote cell proliferation, survival, and invasion via activation of EGF family members including EGF^168^. Collectively, although it is evident that specific growth factor receptors, oncoproteins, proteases, and chemoattractants are key regulators of the lung and other sites of tumour metastasis, changes in the lung tumour microenvironment to facilitate local progression of MM by EVs and other secreted networks^17^ remains to be investigated in the context of MM and its progression.

## Summary

Our findings highlight that exosomes derived from MM tumour cells contain select cargo proteins known to be associated with angiogenesis, cell migration, metastasis, and immunoregulation. Using quantitative proteomics, pathway and network analyses, EV database resources, and bioinformatics analyses, we report a mesothelioma-enriched exosome protein cancer signature (mEXOS), associated with tumour antigens and various cancer-specific signalling (HPGD, ENO1, EDIL3, OSMR) and secreted modulators (FN1, ITLN1, MAMDC2, PDGFD, GBP1). To our knowledge, this is the first demonstration of selective enrichment in exosomes of immunomodulatory components (T- and B-cell immune responses and MHC I/II-peptide antigen processing and presentation), signal transduction molecules (ALCAM, HSP90AA1, LGALS1, TNIK), and metastatic factors (MET, MIF, S100A10, S100A11, TNC, MMP2, ADAM10, ADAMTS1) in different human mesothelioma models. We also demonstrate the functional importance of tumour exosomes, revealing for the first time, that MM-derived exosomes from distinct human models, stimulate fibroblast and endothelial cell migration, and promote endothelial cell angiogenesis.

Presently, there appear to be more questions than answers in terms of what mechanisms, functions, and role of these distinct exosomes associated with MM progression in various cell types, including stromal and tumour cells, in addition to immune cells. Chronic pulmonary inflammation has long been a hallmark of asbestos deposition and is thought to contribute to asbestos-related carcinogenesis. Measures of inflammation such as high neutrophil/lymphocyte ratio have been attributed with angiogenesis, cellular proliferation and prognosis in MM patients^169^. Taken together, comprehensive quantitative proteomic analysis of exosomes secreted by MM tumour cells has revealed a large number of candidate and clinically-relevant extracellular molecules in the regulation of tumour progression, migration, cell transformation, metastasis and in immunoregulation. This information could represent potential specific diagnostic targets for factors of MM origin. The biological significance of our findings are highlighted by the oncogenic effect of exosomes on directly influencing cells in the tumour microenvironment (fibroblasts/endothelial cells). Further functional insights and clinical correlation of these enriched exosomal cargo components in biofluids will extend our understanding of the development of mesothelioma, and mechanisms of how the extracellular environment can contribute to and regulate its progression. Tumour-derived exosomes and their cargo therefore represent exciting and potentially early targets for circulating markers of MM (including tumour-derived antigens), and in the design of targeted immunomodulatory therapeutics.

## Methods

### Cell culture

Human mesothelioma JO38, JU77, OLD1612, and LO68 cells were established from different patients presenting with malignant pleural mesothelioma from the National Centre for Asbestos Related Diseases (NCARD) as described^170^. Cells were cultured and maintained in RPMI 1640 (Invitrogen-GIBCO, Carlsbad, USA) supplemented with 2 mM L-Glutamine, 25 mM HEPES, and 5% foetal calf serum (FCS) (Invitrogen-GIBCO), at 37 °C with 10% CO_2_. Mouse embryonic fibroblasts (MEFs) and human endothelial HUVECs were obtained from American Type Culture Collection (Manassas, VA, USA). MEFs and HUVECs were cultured and maintained in Dulbecco’s Modified Eagle’s Medium (DMEM) with 10% FCS at 37 °C with 10% CO_2_.

### Exosome isolation

JO38, JU77, LO68, and OLD1612 cells (150-mm culture dish, total of 90 dishes, ~8 × 10^8^) were cultured to 70% confluence in RPMI-1640 + 5% FCS, washed three times with RPMI 1640 supplemented with 0.5% insulin-transferrin-selenium (ITS), and 100 μg/mL streptomycin, and cultured in this medium for 24 hr. These serum-free cell culture conditions were initially optimized with assessment for cell morphology and viability. Cell viability of MM cells were measured using the Trypan blue assay following 24 hr culture in RPMI 1640 containing 5% FCS. Viability was expressed as percentage of viable cells from total cells and presented as mean ± SEM. Conditioned media (CM) was collected (~900 mL) and centrifuged (500× *g* for 5 min, 2000× *g* for 10 min) as described^49^,^171^. Supernatants were centrifuged at 10,000× *g* for 30 min at 4 °C, and the resulting supernatants at 100,000× *g* for 1 hr to isolate exosomes^50^,^56^,^57^,^171^. Exosomes were washed in PBS, and ultracentrifuged at 100,000× *g* for 1 hr, as previously described^172^. Pellets were resuspended in 50 μl of PBS for downstream analysis^56^.

### Protein quantification and immunoblotting

Protein content was estimated by 1D-SDS-PAGE/SYPRO^®^ Ruby protein staining densitometry, as previously described^173^. For immunoblotting (10 μg), membranes were probed with primary antibodies [mouse anti-TSG101 (BD Transduction Laboratories; 1:500), mouse anti-Alix (Cell Signaling Technology; 1:1000), rabbit anti-CD70 (Abcam; 1:1000), mouse anti-HSPG1 (Abcam; 1:200), rabbit anti-MSLN (Cell Signaling Technology; 1:1000), rabbit anti-FAT1 (GeneTex; 1:2000), mouse anti-CD81 (Santa Cruz Biotechnology; 1:1000), rabbit anti-CALR (Abcam; 1:1000) for 3 hr at room temperature (RT) in 50 mM Tris, 150 mM NaCl, 0.05% (v/v) Tween 20 (TTBS) followed by incubation with either IRDye 800 goat anti-mouse or goat IgG or IRDye 680 goat anti-rabbit IgG (1:15000, LI-COR Biosciences) for 1 hr at RT in TTBS. Immunoblots were visualized using the Odyssey Infrared Imaging System and Image Studio™ Software (v3.0, LI-COR Biosciences, Nebraska USA).

### Transmission electron microscopy

Exosome samples (2 μg in 10 μl PBS) were applied to 400 mesh carbon-coated copper grids and negatively stained with 10 μL of a 2% uranyl acetate solution for 10 min (ProSciTech, Queensland, Australia). Grids were air dried and viewed using a JEOL JEM-2010 transmission electron microscope operated at 80 kV as previously described^173^.

### Proteomic analysis

Proteomic experiments were performed in biological duplicate (n = 2) as previously described, with MIAPE-compliance^171^,^174^. Briefly, exosomes from each cell line (10 μg) were lysed in SDS sample buffer (4% (w/v) SDS, 20% (v/v) glycerol, 0.01% (v/v) bromophenol blue, 0.125 M Tris-HCl, pH 6.8), and proteins separated by short-range SDS-PAGE (10 × 6 mm), and visualized by Imperial Protein Stain (Invitrogen). Individual samples were excised into equal fractions (n = 2), destained (50 mM ammonium bicarbonate/acetonitrile), reduced (10 mM DTT (Calbiochem) for 30 min), alkylated (50 mM iodoacetic acid (Fluka) for 30 min) and trypsinized (0.2 μg trypsin (Promega Sequencing Grade) for 16 hr at 37 °C). A nanoflow UPLC instrument (Ultimate 3000 RSLCnano, Thermo Fisher Scientific) was coupled on-line to an LTQ Orbitrap Elite mass spectrometer (Thermo Fisher Scientific) with a nanoelectrospray ion source (Thermo Fisher Scientific). Peptides were loaded (Acclaim PepMap100, 5 mm × 300 μm i.d., μ-Precolumn packed with 5 μm C18 beads, Thermo Fisher Scientific) and separated (Acquity UPLC M-Class Peptide BEH130, C18, 1.7 μm, 75 μm × 250 mm, Waters). Data was acquired using Xcalibur software v2.1 (Thermo Fisher Scientific). Details of the operation of the mass spectrometer are described previously^174^.

### Database searching and protein identification

Raw data were processed using Proteome Discoverer (v2.1, Thermo Fisher Scientific). MS2 spectra were searched with Mascot (v2.4, Matrix Science), and Sequest HT (v2.1, Thermo Fisher Scientific) against a database of 133,798 ORFs (UniProtHuman, Apr 2016). Peptide lists were generated from a tryptic digestion with up to two missed cleavages, carbamidomethylation of cysteines as fixed modifications, and oxidation of methionines and protein N-terminal acetylation as variable modifications. Precursor mass tolerance was 10 ppm, product ions were searched at 0.06 Da tolerances, minimum peptide length defined at 6, maximum peptide length 144, and max delta CN 0.05. Peptide spectral matches (PSM) were validated using Percolator based on q-values at a 1% false discovery rate (FDR)^175^,^176^. With Proteome Discoverer, peptide identifications were grouped into proteins according to the law of parsimony and filtered to 1% FDR^177^. Scaffold Q+S (v4.5.3, Proteome Software Inc) was employed to validate MS/MS-based peptide and protein identifications from database searching. Initial peptide identifications were accepted if they could be established at greater than 95% probability (PEP 5%) as specified by the Peptide Prophet algorithm^178^. Protein probabilities were assigned by the Protein Prophet algorithm^177^. Protein identifications were accepted, if they reached greater than 99% probability and contained at least 2 identified unique peptides. These identification criteria typically established <1% false discovery rate based on a decoy database search strategy at the protein level. Proteins that contained similar peptides and could not be differentiated based on MS/MS analysis alone, were grouped to satisfy the principles of parsimony. Contaminants, and reverse identification were excluded from further data analysis. UniProt was used for protein annotation. Raw mass spectrometry data is deposited in the PeptideAtlas and can be accessed at http://www.peptideatlas.org/PASS/PASS00812.

### Label-free spectral counting

Significant spectral count (SpC) and fold change ratios (Rsc) were determined as previously described^49^,^50^,^56^,^171^,^172^,^173^. The relative abundance of a protein within a sample was estimated using normalized SpC, where for each individual protein, significant peptide MS/MS spectra (i.e., ion score greater than identity score) were summated, and normalized by the total number of significant MS/MS spectra identified in the sample. For each protein the Fisher’s exact test was applied to significant assigned spectra. The resulting p-values were corrected for multiple testing using the Benjamini-Hochberg procedure^179^ and statistics performed as previously described^50^. For pathway analyses, KEGG (http://www.genome.jp/kegg/pathway.html) and DAVID (http://david.abcc.ncifcrf.gov/) resources were utilised. Clustering of samples was performed by principal component analysis (PCA) and visualised using ggplot2^180^ and ggfortify (https://cran.r-project.org/web/packages/ggfortify/index.html). The heat map of proteins was performed using gplots (https://cran.r-project.org/web/packages/gplots/index.html).

### Cell migration assay

Cell transwell migration assay was performed as described^181^. Briefly, MEF, neoHFF, and HUVEC cells (5 × 10^4^) in suspension were pelleted at 500× *g* for 5 min, and resuspended in 100 μL of DMEM. Cells were overlaid onto Transwell^®^ polycarbonate membrane cell culture inserts (8.0 μm pore size, Corning), and inserts placed into 24-well companion plates. The bottom chamber contained DMEM (0% FCS) and was supplemented with vehicle control (DMEM), and volume control (DMEM), or MM-derived exosomes (30 μg/μL). Cell migration through the transwell was performed at 37 °C for 24 hr. Inserts were removed, and cells fixed (4% (v/v) formaldehyde, 10 min) and nuclei stained with DAPI. Non-migrating cells were removed from the upper side of the inserts using cotton swabs. Migrating cells were imaged using an inverted Nikon Eclipse TE300 microscope equipped with an attached 12.6 mp digital camera (Nikon DXM1200C) (n = 3; average ± SEM, *p < 0.05, **p < 0.01).

### Endothelial cell tube formation assay

Endothelial cell tube formation assays were performed as previously described^174^. Briefly, HUVECs (7 × 10^4^ cells/well) were re-suspended in DMEM + 5% FBS, and seeded onto growth factor-reduced BD matrigel (1 mg/mL) for 1 hr. Cells were supplemented for 2 hr with vehicle control (DMEM), volume control (DMEM), or MM-derived exosomes (30 μg/μL). Tube formation was analysed (24 hr culture at 37 °C) and imaged using inverted Nikon Eclipse TE300 microscope equipped with an attached 12.6 mp digital camera (Nikon (n = 3; average ± SEM, *p < 0.05)).

### Experimental design and statistical rationale

All methods were carried out in accordance with the approved guidelines of La Trobe Institute for Molecular Science. Functional cell assays were conducted by a minimum of 3 independent biological experiments. For all assays, statistical analysis was performed using Student’s t-tests using GraphPad Prism (v5.0), with *p < 0.05 and **p < 0.01 considered statistically significant. Mass spectrometry analysis of exosome proteins was performed in biological replicates, and only proteins identified in both biological replicates used for label-free quantification. Statistical testing of proteomic data was performed using a Poisson distribution with EdgeR software (v3.2), with *p < 0.05 considered statistically significant. Furthermore, selected proteomic findings were validated using orthogonal approaches including western immuno-blotting performed in biological replicates.

## Additional Information

**How to cite this article**: Greening, D. W. *et al*. Secreted primary human malignant mesothelioma exosome signature reflects oncogenic cargo. *Sci. Rep.* **6**, 32643; doi: 10.1038/srep32643 (2016).

## Supplementary Material

Supplementary Information

Supplementary Dataset

## Figures and Tables

**Table 1:** Mesothelioma-associated protein cargo in exosomes.

Gene Name^a^	Protein Accession^a^	Protein Description^a^	SpC Combined (4 cell lines)^b^	Reported in VesiclePedia^c^
MSLN	Q13421	Mesothelin (CAK1 antigen)	13	Y
TNFAIP2	Q03169	Tumor necrosis factor alpha-induced protein 2	64	Y
CALB2	P22676	Calretinin	7	Y
CALR	P27797	Calreticulin (CRP55)	12	Y
TNFRSF11B	O00300	Tumor necrosis factor receptor superfamily member 11B	9	Y
GOLT1B	Q9Y3E0	Vesicle transport protein GOT1B	7	Y
Q59FU8_HUMAN	Q59FU8	Tumor necrosis factor receptor superfamily, member 6 isoform 1 variant	4	
LGALS1	P09382	Galectin-1	60	Y
SOD1	P00441	Superoxide dismutase 1	9	Y
SOD2	B3KUK2	Superoxide dismutase 2	7	Y
STAT1	P42224	Signal transducer and activator of transcription 1-alpha/beta	139	Y
STAT2	P52630	Signal transducer and activator of transcription 2 (p113)	6	Y
STAT3	K7ENL3	Signal transducer and activator of transcription	25	Y
TNFRSF10A	O00220	Tumor necrosis factor receptor superfamily member 10A	8	Y
TPT1	Q5W0H4	Translationally-controlled tumor protein	7	Y
VIM	P08670	Vimentin	180	Y

^a^Protein accession and description derived from Gene Ontology (UniProt).

^b^Combined spectral counts (SpC) observed for mesothelioma exosome datasets. Contribution of individual SpC for each cell lines reported in Supplementary Dataset.

^c^Comparison with extracellular vesicle database, Vesiclepedia^62^. Y indicates reported in the database.

**Table 2:** Tumour-associated antigens identified in mesothelioma-derived exosomes.

	Gene Name^a^	Protein Accession^a^	Protein Description^a^	SpC Combined (4 cell lines)^b^	Reported in VesiclePedia^c^
Tumour antigens	MCAM	A8K6A6	Melanoma cell adhesion molecule	31	Y
	CD70	P32970	CD70 antigen (CD27 ligand)	11	Y
	MAGED2	Q9UNF1	Melanoma-associated antigen D2	10	Y
	GPC1	H7C410	Glypican-1	44	Y
	CPSF1	Q10570	Cleavage and polyadenylation specificity factor subunit 1	9	Y
	CPSF3	Q05BZ5	CPSF3 protein	2	Y
	Q9NXW1_HUMAN	Q9NXW1	BJ-HCC-24 tumor antigen	6	Y
	ARMC9	Q7Z3E5	LisH domain-containing protein ARMC9 (Melanoma/melanocyte-specific tumor antigen)	10	Y
	MSLN	Q13421	Mesothelin (CAK1 antigen)	13	Y
	ANXA1	P04083	Annexin A1	268	Y
	ANXA2	P07355	Annexin A2	643	Y
	ANXA3	P12429	Annexin A3	95	Y
	ANXA4	P09525	Annexin A4	167	Y
	ANXA5	P08758	Annexin A5	351	Y
	ANXA6	A6NN80	Annexin A6	205	Y
	ANXA11	P50995	Annexin A11	102	Y
Overexpressed/accumulated in tumours	MFI2	P08582	Melanotransferrin (Melanoma-associated antigen p97)	87	Y
	CDK1	P06493	Cyclin-dependent kinase 1	48	Y
	MUC5AC	P98088	Mucin-5AC	176	Y
	MMP2	P08253	72 kDa type IV collagenase	12	Y
	FGFBP1	Q14512	Fibroblast growth factor-binding protein 1	11	Y
	PXDN	Q92626	Peroxidasin homolog (Melanoma-associated antigen MG50)	60	Y
	MUC5B	E9PBJ0	Mucin-5B	55	Y
	FAT1	Q14517	Protocadherin Fat 1	30	Y
	MUC13	Q9H3R2	Mucin-13	4	Y
	TGFBI	Q15582	Transforming growth factor-beta-induced protein ig-h3	112	Y
	MIF	P14174	Macrophage migration inhibitory factor	18	Y

^a^Protein accession and description derived from Gene Ontology (UniProt).

^b^Combined spectral counts (SpC) observed for mesothelioma exosome datasets. Contribution of individual SpC for each cell lines reported in Supplementary Dataset.

^c^Comparison with extracellular vesicle database, VesiclePedia^62^. Y indicates reported in the database.

**Table 3:** Metastatic-niche-associated proteins in mesothelioma exosomes.

Gene Name^a^	Protein Description^a^	SpC Combined (4 cell lines)^b^	Reported in VesiclePedia^c^
ADAM10	Disintegrin and metalloproteinase domain-containing protein 10	20	Y
ADAMTS1	A disintegrin and metalloproteinase with thrombospondin motifs 1	15	Y
ANXA1	Annexin A1 (Annexin I)	268	Y
ANXA11	Annexin A11 (56 kDa autoantigen)	102	Y
ARHGDIA	Rho GDP-dissociation inhibitor 1	29	Y
CD44	CD44 antigen	97	Y
GRB2	Growth factor receptor-bound protein 2	6	Y
ITGA2	Integrin alpha-2 (CD49 antigen-like family member B)	53	Y
ITGB4	Integrin beta	64	Y
MET	Hepatocyte growth factor receptor (HGF receptor)	21	Y
MIF	Macrophage migration inhibitory factor	18	Y
MMP2	72 kDa type IV collagenase	12	Y
RPLP2	60S acidic ribosomal protein P2 (Renal carcinoma antigen NY-REN-44)	39	Y
S100A10	S100 calcium binding protein A10	42	Y
S100A11	Protein S100-A11 (Calgizzarin)	41	Y
S100A6	Protein S100-A6	18	Y
SRC	Proto-oncogene tyrosine-protein kinase Src	52	Y
STUB1	E3 ubiquitin-protein ligase CHIP	9	Y
TNC	TNC variant protein	16	Y

^a^Protein accession and description derived from Gene Ontology (UniProt).

^b^Combined spectral counts (SpC) observed for mesothelioma exosome datasets. Contribution of individual SpC for each cell lines reported in Supplementary Dataset.
