# Supplementary material for: Secreted primary human malignant mesothelioma exosome signature reflects oncogenic cargo
Source: Sci Rep. 2016 Sep 8;6:32643. doi: 10.1038/srep32643 (PMC5015102; doi:10.1038/srep32643)
Supplement: Supplementary Information [file srep32643-s1.pdf]

Supplementary Information

Secreted primary human malignant mesothelioma exosome signature reflects oncogenic cargo

David W. Greening[1,*], Hong Ji[1], Maoshan Chen[1], Bruce W. S. Robinson[2,3], Ian M. Dick[2], Jenette Creaney[2,4], Richard J. Simpson[1]

[1] *Department of Biochemistry and Genetics, La Trobe Institute for Molecular Science, La Trobe University, Melbourne, Victoria 3086, Australia*

[2] *National Centre for Asbestos Related Diseases, School of Medicine and Pharmacology, University of Western Australia, 6009, Australia*

[3] *Department of Respiratory Medicine, Sir Charles Gairdner Hospital, Perth, Western Australia, 6009, Australia*

[4] *Australian Mesothelioma Tissue Bank, Sir Charles Gairdner Hospital, Perth, Western Australia, 6009, Australia*

*** To whom correspondence should be addressed:**

Dr. David W. Greening

La Trobe Institute for Molecular Science

La Trobe University

Bundoora, Victoria 3086, Australia

Tel: +61 03 9479 5031

Email: d.greening@latrobe.edu.au

Figure S1

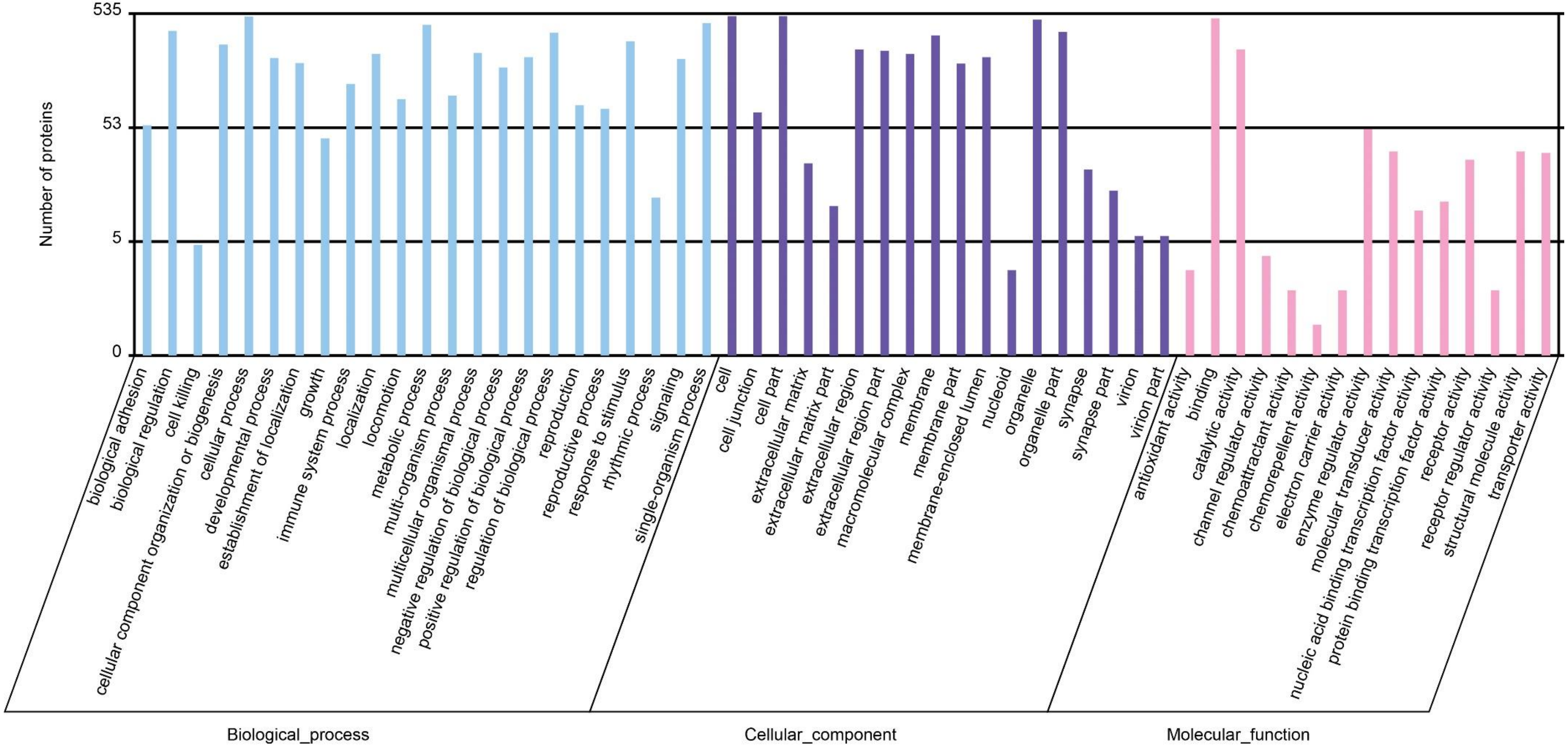

Figure S1. Gene Ontology assignment of the proteins identified in mEXOS, revealing enriched categories associated with biological process, cellular component and molecular function.

Table S1. Proteins commonly identified in all mesothelioma cell-derived exosomes.

Gene symbol	Gene Name	Protein Accession	Protein Description	Coverage (%)	SpC Combined (4 cell lines)	JO38 SpC	JU77 SpC	LO68 SpC	OLD1612 SpC	Vesiclepedia
KPYM_HUMAN	PKM	P14618	Pyruvate kinase PKM (EC 2.7.1.40) (Cytosolic thyroid hormone-binding p	61.2	824	114	120	192	414	Y
DYHC1_HUMAN	DYNC1H1	Q14204	Cytoplasmic dynein 1 heavy chain 1 (Cytoplasmic dynein heavy chain 1)	30.2	750	198	71	175	320	Y
ANXA2_HUMAN	ANXA2	P07355	Annexin A2 (Annexin II) (Annexin-2) (Calpactin I heavy chain) (Calpact	74.9	643	172	199	163	116	Y
ACTB_HUMAN	ACTB	P60709	Actin, cytoplasmic 1 (Beta-actin) [Cleaved into: Actin, cytoplasmic 1,	49.1	640	173	121	133	223	Y
TBB5_HUMAN	TUBB	P07437	Tubulin beta chain (Tubulin beta-5 chain)	48.9	637	150	88	136	276	Y
Q8IWP6_HUMAN	Q8IWP6_HUMAN	Q8IWP6	Class IVb beta tubulin	48.8	544	127	71	116	239	
TBB2A_HUMAN	TUBB2A	Q13885	Tubulin beta-2A chain (Tubulin beta class IIa)	48.8	540	124	70	125	238	Y
HSP7C_HUMAN	HSPA8	P11142	Heat shock cognate 71 kDa protein (Heat shock 70 kDa protein 8)	42.7	529	143	126	134	124	Y
FLNA_HUMAN	FLNA	P21333	Filamin-A (FLN-A) (Actin-binding protein 280) (ABP-280) (Alpha-filamin	33.1	509	158	80	170	109	Y
FAS_HUMAN	FASN	P49327	Fatty acid synthase (EC 2.3.1.85) [Includes: [Acyl-carrier-protein] S-	30.7	506	164	61	196	87	Y
HS90B_HUMAN	HSP90AB1	P08238	Heat shock protein HSP 90-beta (HSP 90) (Heat shock 84 kDa) (HSP 84) (	40.6	498	141	102	136	121	Y
HS90A_HUMAN	HSP90AA1	P07900	Heat shock protein HSP 90-alpha (Heat shock 86 kDa) (HSP 86) (HSP86) (	39.9	489	125	118	118	129	Y
PLEC_HUMAN	PLEC	Q15149	Plectin (PCN) (PLTN) (Hemidesmosomal protein 1) (HD1) (Plectin-1)	26.3	482	207	47	153	84	Y
CLH1_HUMAN	CLTC	Q00610	Clathrin heavy chain 1 (Clathrin heavy chain on chromosome 17) (CLH-17	30.7	473	166	70	141	100	Y
Q4LE36_HUMAN	ACLY	Q4LE36	ACLY variant protein (Fragment)	50.7	473	130	41	251	54	Y
TBB4A_HUMAN	TUBB4A	P04350	Tubulin beta-4A chain (Tubulin 5 beta) (Tubulin beta-4 chain)	44.4	470	110	62	99	205	Y
B4DVE1_HUMAN	B4DVE1_HUMAN	B4DVE1	cDNA FLJ53478, highly similar to Galectin-3-binding protein	36.6	465	178	94	135	60	
B3KPS3_HUMAN	B3KPS3_HUMAN	B3KPS3	cDNA FLJ32131 fis, clone PEBLM2000267, highly similar to Tubulin alpha	48.3	455	113	58	111	177	
IQGA1_HUMAN	IQGAP1	P46940	Ras GTPase-activating-like protein IQGAP1 (p195)	27.5	418	118	119	71	112	Y
ACTN4_HUMAN	ACTN4	O43707	Alpha-actinin-4 (F-actin cross-linking protein) (Non-muscle alpha-acti	48.2	406	108	96	124	83	Y
MOES_HUMAN	MSN	P26038	Moesin (Membrane-organizing extension spike protein)	53.6	405	149	94	39	128	Y
TBB3_HUMAN	TUBB3	Q13509	Tubulin beta-3 chain (Tubulin beta-4 chain) (Tubulin beta-III)	43.6	403	97	50	103	156	Y
MYH9_HUMAN	MYH9	P35579	Myosin-9 (Cellular myosin heavy chain, type A) (Myosin heavy chain 9)	35.3	397	194	63	28	118	Y

FLNB_HUMAN	FLNB	O75369	Filamin-B (FLN-B) (ABP-278) (ABP-280 homolog) (Actin-binding-like prot	28.5	372	146	33	129	72	Y
TBB6_HUMAN	TUBB6	Q9BUF5	Tubulin beta-6 chain (Tubulin beta class V)	37.2	364	93	45	73	166	Y
EF2_HUMAN	EEF2	P13639	Elongation factor 2 (EF-2)	38.9	358	81	44	97	140	Y
MYOF_HUMAN	MYOF	Q9NZM1	Myoferlin (Fer-1-like protein 3)	32.9	355	150	120	19	69	Y
ACTBL_HUMAN	ACTBL2	Q562R1	Beta-actin-like protein 2 (Kappa-actin)	24.5	354	94	74	74	112	Y
ANXA5_HUMAN	ANXA5	P08758	Annexin A5 (Anchorin CII) (Annexin V) (Annexin-5) (Calphobindin I) (CB	66.3	351	100	90	96	71	Y
PGBM_HUMAN	HSPG2	P98160	Basement membrane-specific heparan sulfate proteoglycan core protein (	17.8	342	150	34	159	2	Y
HSP71_HUMAN	HSPA1A	P08107	Heat shock 70 kDa protein 1A/1B (Heat shock 70 kDa protein 1/2) (HSP70	39.6	327	62	53	113	98	Y
AT1A1_HUMAN	ATP1A1	P05023	Sodium/potassium-transporting ATPase subunit alpha-1 (Na(+)/K(+) ATPas	37.5	317	149	63	20	89	Y
ENOA_HUMAN	ENO1	P06733	Alpha-enolase (EC 4.2.1.11) (2-phospho-D-glycerate hydro-lyase) (C-myc	46.8	307	87	60	75	94	Y
GRP78_HUMAN	HSPA5	P11021	78 kDa glucose-regulated protein (GRP-78) (Endoplasmic reticulum lumen	38.1	295	99	98	58	45	Y
COCA1_HUMAN	COL12A1	Q99715	Collagen alpha-1(XII) chain	21.7	291	160	68	21	44	Y
ACTN1_HUMAN	ACTN1	P12814	Alpha-actinin-1 (Alpha-actinin cytoskeletal isoform) (F-actin cross-li	44.7	288	114	72	44	64	Y
MVP_HUMAN	MVP	Q14764	Major vault protein (MVP) (Lung resistance-related protein)	42.8	274	48	36	163	31	Y
PDC6I_HUMAN	PDCD6IP	Q8WUM4	Programmed cell death 6-interacting protein (PDCD6-interacting protein	42.7	273	59	70	99	49	Y
AHNK_HUMAN	AHNAK	Q09666	Neuroblast differentiation-associated protein AHNAK (Desmoyokin)	14.9	270	84	74	10	110	Y
ANXA1_HUMAN	ANXA1	P04083	Annexin A1 (Annexin I) (Annexin-1) (Calpactin II) (Calpactin-2) (Chrom	61.6	268	104	35	77	56	Y
EF1A1_HUMAN	EEF1A1	P68104	Elongation factor 1-alpha 1 (EF-1-alpha-1) (Elongation factor Tu) (EF-	29.7	265	68	40	64	102	Y
G3P_HUMAN	GAPDH	P04406	Glyceraldehyde-3-phosphate dehydrogenase (GAPDH) (EC 1.2.1.12) (Peptid	43	262	95	25	79	69	Y
MYO1C_HUMAN	MYO1C	O00159	Unconventional myosin-Ic (Myosin I beta) (MMI-beta) (MMIb)	31.1	261	74	80	18	89	Y
RADI_HUMAN	RDX	P35241	Radixin	52.5	257	57	40	26	141	Y
AGRIN_HUMAN	AGRN	O00468	Agrin [Cleaved into: Agrin N-terminal 110 kDa subunit; Agrin C-termina	17.3	245	69	37	113	28	Y
TERA_HUMAN	VCP	P55072	Transitional endoplasmic reticulum ATPase (TER ATPase) (EC 3.6.4.6) (1	36.5	241	63	24	95	61	Y
B2R6J2_HUMAN	VIL2	B2R6J2	Villin 2 (ezrin) (VIL2)	39.6	238	78	52	34	78	
ITA3_HUMAN	ITGA3	P26006	Integrin alpha-3 (CD49 antigen-like family member C) (FRP-2) (Galactop	21.9	227	79	64	13	73	Y
K6PP_HUMAN	PFKP	Q01813	6-phosphofructokinase type C (EC 2.7.1.11) (6-phosphofructokinase, pla	29.2	219	70	21	60	72	Y
TLN1_HUMAN	TLN1	Q9Y490	Talin-1	17.3	214	93	26	41	56	Y

LAMC1_HUMAN	LAMC1	P11047	Laminin subunit gamma-1 (Laminin B2 chain) (Laminin-1 subunit gamma) (	21.5	210	78	29	100	3	Y
EHD1_HUMAN	EHD1	Q9H4M9	EH domain-containing protein 1 (PAST homolog 1) (hPAST1) (Testilin)	44	206	77	60	22	50	Y
A6NN80_HUMAN	ANXA6	A6NN80	Annexin A6	42.1	205	73	70	21	45	Y
A8K7F6_HUMAN	A8K7F6_HUMAN	A8K7F6	cDNA FLJ78244, highly similar to Homo sapiens eukaryotic translation i	45.3	202	50	28	46	79	
Q06AH7_HUMAN	TF	Q06AH7	Transferrin	38.4	202	58	26	104	17	Y
4F2_HUMAN	SLC3A2	P08195	4F2 cell-surface antigen heavy chain (4F2hc) (4F2 heavy chain antigen)	34.6	196	71	44	48	34	Y
A8K8D9_HUMAN	A8K8D9_HUMAN	A8K8D9	Glucose-6-phosphate 1-dehydrogenase (EC 1.1.1.49)	57.9	190	57	10	107	22	
UBA1_HUMAN	UBA1	P22314	Ubiquitin-like modifier-activating enzyme 1 (Protein A1S9) (Ubiquitin-	24.1	188	48	34	67	43	Y
F8VPD4_HUMAN	CAD	F8VPD4	CAD protein	16.6	183	52	5	61	65	Y
5NTD_HUMAN	NT5E	P21589	5'-nucleotidase (5'-NT) (EC 3.1.3.5) (Ecto-5'-nucleotidase) (CD antige	34.1	181	83	17	12	76	Y
A8K6Q8_HUMAN	A8K6Q8_HUMAN	A8K6Q8	cDNA FLJ75881, highly similar to Homo sapiens transferrin receptor (p9	35.9	181	73	45	20	45	
VIME_HUMAN	VIM	P08670	Vimentin	54.3	180	42	34	46	61	Y
PGK1_HUMAN	PGK1	P00558	Phosphoglycerate kinase 1 (EC 2.7.2.3) (Cell migration-inducing gene 1	55.9	180	64	22	58	38	Y
C1TC_HUMAN	MTHFD1	P11586	C-1-tetrahydrofolate synthase, cytoplasmic (C1-THF synthase) [Cleaved	35.5	172	34	16	38	89	Y
IMB1_HUMAN	KPNB1	Q14974	Importin subunit beta-1 (Importin-90) (Karyopherin subunit beta-1) (Nu	26.5	168	47	27	52	42	Y
ANXA4_HUMAN	ANXA4	P09525	Annexin A4 (35-beta calcimedin) (Annexin IV) (Annexin-4) (Carbohydrate	61.4	167	41	28	76	23	Y
XRCC6_HUMAN	XRCC6	P12956	X-ray repair cross-complementing protein 6 (EC 3.6.4.-) (EC 4.2.99.-)	31.7	166	37	6	74	50	Y
PROF1_HUMAN	PFN1	P07737	Profilin-1 (Epididymis tissue protein Li 184a) (Profilin I)	67.1	164	44	26	53	44	Y
Q59E99_HUMAN	Q59E99_HUMAN	Q59E99	Thrombospondin 1 variant (Fragment)	21.6	164	39	22	88	16	
H2B1D_HUMAN	HIST1H2BD	P58876	Histone H2B type 1-D (HIRA-interacting protein 2) (Histone H2B.1 B) (H	58.7	163	32	23	30	78	Y
SYAC_HUMAN	AARS	P49588	Alanine--tRNA ligase, cytoplasmic (EC 6.1.1.7) (Alanyl-tRNA synthetase	26.3	158	41	9	51	57	Y
H4_HUMAN	HIST1H4A	P62805	Histone H4	58.3	152	31	20	22	86	Y
GNAI2_HUMAN	GNAI2	P04899	Guanine nucleotide-binding protein G(i) subunit alpha-2 (Adenylate cyc	37.5	150	43	38	3	66	Y
PABP1_HUMAN	PABPC1	P11940	Polyadenylate-binding protein 1 (PABP-1) (Poly(A)-binding protein 1)	32.1	150	57	17	35	44	Y
ITB1_HUMAN	ITGB1	P05556	Integrin beta-1 (Fibronectin receptor subunit beta) (Glycoprotein IIa)	16.3	149	43	46	16	46	Y
XPO2_HUMAN	CSE1L	P55060	Exportin-2 (Exp2) (Cellular apoptosis susceptibility protein) (Chromos	23.9	144	38	18	44	44	Y
COPA_HUMAN	COPA	P53621	Coatomer subunit alpha (Alpha-coat protein) (Alpha-COP) (HEP-COP) (HEP	22.9	143	40	18	30	56	Y

HSP74_HUMAN	HSPA4	P34932	Heat shock 70 kDa protein 4 (HSP70RY) (Heat shock 70-related protein A	23.2	143	38	23	43	39	Y
XPO1_HUMAN	XPO1	O14980	Exportin-1 (Exp1) (Chromosome region maintenance 1 protein homolog)	24	142	39	10	52	42	Y
Q59GM9_HUMAN	Q59GM9_HUMAN	Q59GM9	Phosphorylase (EC 2.4.1.1) (Fragment)	28.7	139	20	4	53	66	
STAT1_HUMAN	STAT1	P42224	Signal transducer and activator of transcription 1-alpha/beta (Transcr	34.9	139	83	6	34	21	Y
ATPB_HUMAN	ATP5B	P06576	ATP synthase subunit beta, mitochondrial (EC 3.6.3.14)	38.4	136	10	8	63	60	Y
G3XAI2_HUMAN	LAMB1	G3XAI2	Laminin subunit beta-1 (Laminin, beta 1, isoform CRA_a)	18.4	136	42	7	90	1	Y
1433E_HUMAN	YWHAE	P62258	14-3-3 protein epsilon (14-3-3E)	59.6	135	34	30	14	58	Y
EIF3A_HUMAN	EIF3A	Q14152	Eukaryotic translation initiation factor 3 subunit A (eIF3a) (Eukaryot	17.2	134	42	15	39	39	Y
LDHA_HUMAN	LDHA	P00338	L-lactate dehydrogenase A chain (LDH-A) (EC 1.1.1.27) (Cell proliferat	45.5	134	50	24	25	37	Y
PPIA_HUMAN	PPIA	P62937	Peptidyl-prolyl cis-trans isomerase A (PPIase A) (EC 5.2.1.8) (Cycloph	58.2	134	42	29	30	35	Y
PUR9_HUMAN	ATIC	P31939	Bifunctional purine biosynthesis protein PURH [Includes: Phosphoribosy	39.5	133	43	10	46	37	Y
VINC_HUMAN	VCL	P18206	Vinculin (Metavinculin) (MV)	28.8	132	65	17	10	44	Y
Q8N9M2_HUMAN	Q8N9M2_HUMAN	Q8N9M2	cDNA FLJ36887 fis, clone BNGH42005504, highly similar to 26S PROTEASOM	36.1	131	41	20	34	36	
PSMD2_HUMAN	PSMD2	Q13200	26S proteasome non-ATPase regulatory subunit 2 (26S proteasome regulat	27.8	130	38	9	44	41	Y
STOM_HUMAN	STOM	P27105	Erythrocyte band 7 integral membrane protein (Protein 7.2b) (Stomatin)	45.8	128	55	40	21	13	Y
PRS6A_HUMAN	PSMC3	P17980	26S protease regulatory subunit 6A (26S proteasome AAA-ATPase subunit	36.2	127	41	13	31	44	Y
LDHB_HUMAN	LDHB	P07195	L-lactate dehydrogenase B chain (LDH-B) (EC 1.1.1.27) (LDH heart subun	48.5	127	50	13	23	44	Y
PSD11_HUMAN	PSMD11	O00231	26S proteasome non-ATPase regulatory subunit 11 (26S proteasome regula	35.3	126	39	11	43	36	Y
TCPB_HUMAN	CCT2	P78371	T-complex protein 1 subunit beta (TCP-1-beta) (CCT-beta)	38.9	126	42	17	41	26	Y
PLST_HUMAN	PLS3	P13797	Plastin-3 (T-plastin)	33.7	125	48	44	8	28	Y
SF3B3_HUMAN	SF3B3	Q15393	Splicing factor 3B subunit 3 (Pre-mRNA-splicing factor SF3b 130 kDa su	20.4	124	15	13	54	46	Y
EPHA2_HUMAN	EPHA2	P29317	Ephrin type-A receptor 2 (EC 2.7.10.1) (Epithelial cell kinase) (Tyros	23.1	123	53	26	7	41	Y
EF1G_HUMAN	EEF1G	P26641	Elongation factor 1-gamma (EF-1-gamma) (eEF-1B gamma)	24.5	122	31	19	36	38	Y
B4DE36_HUMAN	B4DE36_HUMAN	B4DE36	Glucose-6-phosphate isomerase (EC 5.3.1.9)	31.7	122	22	17	48	36	
A6NG51_HUMAN	SPTAN1	A6NG51	Spectrin alpha chain, non-erythrocytic 1	16.1	122	57	10	48	8	Y
DHX9_HUMAN	DHX9	Q08211	ATP-dependent RNA helicase A (RHA) (EC 3.6.4.13) (DEAH box protein 9)	22.9	121	33	5	27	59	Y
VPS35_HUMAN	VPS35	Q96QK1	Vacuolar protein sorting-associated protein 35 (hVPS35) (Maternal-embr	31.3	121	26	25	30	42	Y

RUVB2_HUMAN	RUVBL2	Q9Y230	RuvB-like 2 (EC 3.6.4.12) (48 kDa TATA box-binding protein-interacting	37.4	121	38	2	45	37	Y
E1NZA1_HUMAN	PRIC295	E1NZA1	Peroxisome proliferator activated receptor interacting complex protein	16.5	119	25	4	27	65	
CTNA1_HUMAN	CTNNA1	P35221	Catenin alpha-1 (Alpha E-catenin) (Cadherin-associated protein) (Renal	27.2	119	36	29	14	42	Y
B2RCM2_HUMAN	B2RCM2_HUMAN	B2RCM2	cDNA, FLJ96156, highly similar to Homo sapiens leucyl-tRNA synthetase	24.1	117	27	6	14	71	
C9J9K3_HUMAN	RPSA	C9J9K3	40S ribosomal protein SA (Fragment)	45.5	117	24	18	32	44	Y
GRP75_HUMAN	HSPA9	P38646	Stress-70 protein, mitochondrial (75 kDa glucose-regulated protein) (G	30.6	117	42	16	22	40	Y
B2R5U1_HUMAN	B2R5U1_HUMAN	B2R5U1	cDNA, FLJ92620, highly similar to Homo sapiens staphylococcal nuclease	22.7	116	37	2	47	32	
PYGL_HUMAN	PYGL	P06737	Glycogen phosphorylase, liver form (EC 2.4.1.1)	36.2	115	25	13	26	58	Y
EHD4_HUMAN	EHD4	Q9H223	EH domain-containing protein 4 (Hepatocellular carcinoma-associated pr	42.9	115	37	17	8	53	Y
CYFP1_HUMAN	CYFIP1	Q7L576	Cytoplasmic FMR1-interacting protein 1 (Specifically Rac1-associated p	20.1	115	34	25	23	36	Y
H0Y4R1_HUMAN	IMPDH2	H0Y4R1	Inosine-5'-monophosphate dehydrogenase 2 (Fragment)	31.1	113	33	12	20	48	Y
B4DJ30_HUMAN	B4DJ30_HUMAN	B4DJ30	cDNA FLJ61290, highly similar to Neutral alpha-glucosidase AB	21.9	113	41	19	30	28	
B4DMC0_HUMAN	B4DMC0_HUMAN	B4DMC0	cDNA FLJ55341, highly similar to 116 kDa U5 small nuclear ribonucleopr	22.1	112	15	10	37	50	
BGH3_HUMAN	TGFBI	Q15582	Transforming growth factor-beta-induced protein ig-h3 (Beta ig-h3) (Ke	23.7	112	27	20	28	41	Y
COPB_HUMAN	COPB1	P53618	Coatomer subunit beta (Beta-coat protein) (Beta-COP)	25	112	32	12	34	38	Y
A8K8U1_HUMAN	A8K8U1_HUMAN	A8K8U1	cDNA FLJ77762, highly similar to Homo sapiens cullin-associated and ne	17.4	112	39	27	36	11	
B0QY89_HUMAN	EIF3L	B0QY89	Eukaryotic translation initiation factor 3 subunit L (eIF3l) (Eukaryot	24.4	111	30	14	32	36	Y
E9PDF6_HUMAN	MYO1B	E9PDF6	Unconventional myosin-Ib	19.2	111	39	32	21	19	Y
QSOX1_HUMAN	QSOX1	O00391	Sulfhydryl oxidase 1 (hQSOX) (EC 1.8.3.2) (Quiescin Q6)	26.5	110	59	25	18	7	Y
LMNA_HUMAN	LMNA	P02545	Prelamin-A/C [Cleaved into: Lamin-A/C (70 kDa lamin) (Renal carcinoma	29.4	107	37	34	23	14	Y
CLUS_HUMAN	CLU	P10909	Clusterin (Aging-associated gene 4 protein) (Apolipoprotein J) (Apo-J)	36.7	107	20	11	66	10	Y
RUVB1_HUMAN	RUVBL1	Q9Y265	RuvB-like 1 (EC 3.6.4.12) (49 kDa TATA box-binding protein-interacting	34	106	24	13	35	34	Y
B3KTQ2_HUMAN	B3KTQ2_HUMAN	B3KTQ2	cDNA FLJ38589 fis, clone HCHON2010074, highly similar to LACTADHERIN	36.1	105	17	57	12	19	
SPTB2_HUMAN	SPTBN1	Q01082	Spectrin beta chain, non-erythrocytic 1 (Beta-II spectrin) (Fodrin bet	16.9	105	48	4	47	8	Y
CATD_HUMAN	CTSD	P07339	Cathepsin D (EC 3.4.23.5) [Cleaved into: Cathepsin D light chain; Cath	38.3	105	60	21	23	2	Y
RL4_HUMAN	RPL4	P36578	60S ribosomal protein L4 (60S ribosomal protein L1)	34.4	104	48	12	3	43	Y
PRS7_HUMAN	PSMC2	P35998	26S protease regulatory subunit 7 (26S proteasome AAA-ATPase subunit R	45.5	104	33	3	33	38	Y

B3KX11_HUMAN	B3KX11_HUMAN	B3KX11	cDNA FLJ44436 fis, clone UTERU2019706, highly similar to T-complex pro	29.7	104	29	11	34	31	
K6PL_HUMAN	PFKL	P17858	6-phosphofructokinase, liver type (EC 2.7.1.11) (Phosphofructo-1-kinas	22.4	103	45	10	18	30	Y
Q53HU0_HUMAN	Q53HU0_HUMAN	Q53HU0	Chaperonin containing TCP1, subunit 8 (Theta) variant (Fragment)	42.9	103	32	10	40	23	
ACSL4_HUMAN	ACSL4	O60488	Long-chain-fatty-acid--CoA ligase 4 (EC 6.2.1.3) (Long-chain acyl-CoA	27.4	102	46	26	3	27	Y
PRDX1_HUMAN	PRDX1	Q06830	Peroxiredoxin-1 (EC 1.11.1.15) (Natural killer cell-enhancing factor A	48.7	102	44	15	28	16	Y
HEMO_HUMAN	HPX	P02790	Hemopexin (Beta-1B-glycoprotein)	29	102	22	8	64	9	Y
A8K2T7_HUMAN	A8K2T7_HUMAN	A8K2T7	cDNA FLJ76780, highly similar to Homo sapiens epidermal growth factor	14.6	101	41	22	2	42	
ITA6_HUMAN	ITGA6	P23229	Integrin alpha-6 (CD49 antigen-like family member F) (VLA-6) (CD antig	18.4	101	33	26	11	33	Y
GNAI3_HUMAN	GNAI3	P08754	Guanine nucleotide-binding protein G(k) subunit alpha (G(i) alpha-3)	29.7	100	28	20	11	41	Y
RS3_HUMAN	RPS3	P23396	40S ribosomal protein S3	58	100	31	15	22	35	Y
GNAS1_HUMAN	GNAS	Q5JWF2	Guanine nucleotide-binding protein G(s) subunit alpha isoforms XLas (A	11.7	99	39	19	10	35	Y
RAP1B_HUMAN	RAP1B	P61224	Ras-related protein Rap-1b (GTP-binding protein smg p21B)	49.5	99	37	18	13	32	Y
A8K916_HUMAN	A8K916_HUMAN	A8K916	cDNA FLJ78481, highly similar to Homo sapiens adaptor-related protein	18	99	32	9	35	29	
CLIC1_HUMAN	CLIC1	O00299	Chloride intracellular channel protein 1 (Chloride channel ABP) (Nucle	41.5	98	39	15	10	35	Y
ALDOA_HUMAN	ALDOA	P04075	Fructose-bisphosphate aldolase A (EC 4.1.2.13) (Lung cancer antigen NY	40.9	98	48	15	22	19	Y
B3GQS7_HUMAN	HSPD1	B3GQS7	Mitochondrial heat shock 60kD protein 1 variant 1	25.3	98	30	20	31	18	Y
B3KX72_HUMAN	B3KX72_HUMAN	B3KX72	cDNA FLJ44920 fis, clone BRAMY3011501, highly similar to Heterogeneous	22	97	24	9	12	54	
E7EPC6_HUMAN	CD44	E7EPC6	CD44 antigen	12.6	97	23	19	13	41	Y
SYRC_HUMAN	RARS	P54136	Arginine--tRNA ligase, cytoplasmic (EC 6.1.1.19) (Arginyl-tRNA synthet	28.6	97	31	8	22	37	Y
SYDC_HUMAN	DARS	P14868	Aspartate--tRNA ligase, cytoplasmic (EC 6.1.1.12) (Aspartyl-tRNA synth	41.1	96	28	6	22	41	Y
TCPA_HUMAN	TCP1	P17987	T-complex protein 1 subunit alpha (TCP-1-alpha) (CCT-alpha)	27.7	96	23	12	35	29	Y
SYG_HUMAN	GARS	P41250	Glycine--tRNA ligase (EC 6.1.1.14) (Diadenosine tetraphosphate synthet	26	95	21	4	22	48	Y
PLAK_HUMAN	JUP	P14923	Junction plakoglobin (Catenin gamma) (Desmoplakin III) (Desmoplakin-3)	33.7	95	49	19	19	13	Y
ENPL_HUMAN	HSP90B1	P14625	Endoplasmin (94 kDa glucose-regulated protein) (GRP-94) (Heat shock pr	25	95	44	26	20	9	Y
B2R8R5_HUMAN	B2R8R5_HUMAN	B2R8R5	cDNA, FLJ94025, highly similar to Homo sapiens tripartite motif-contai	20.4	94	2	3	39	50	
AP2A1_HUMAN	AP2A1	O95782	AP-2 complex subunit alpha-1 (100 kDa coated vesicle protein A) (Adapt	17.7	94	20	11	26	37	Y
UGDH_HUMAN	UGDH	O60701	UDP-glucose 6-dehydrogenase (UDP-Glc dehydrogenase) (UDP-GlcDH) (UDPGD	38.7	94	8	1	54	32	Y

H0Y8C6_HUMAN	IPO5	H0Y8C6	Importin-5 (Fragment)	15.3	94	31	13	23	29	Y
NIBL1_HUMAN	FAM129B	Q96TA1	Niban-like protein 1 (Meg-3) (Melanoma invasion by ERK) (MINERVA) (Pro	21.8	94	36	16	14	28	Y
SYEP_HUMAN	EPRS	P07814	Bifunctional glutamate/proline--tRNA ligase (Bifunctional aminoacyl-tR	20.6	93	31	1	12	52	Y
XRCC5_HUMAN	XRCC5	P13010	X-ray repair cross-complementing protein 5 (EC 3.6.4.-) (86 kDa subuni	24.3	93	19	1	46	29	Y
B4DLV7_HUMAN	B4DLV7_HUMAN	B4DLV7	cDNA FLJ60299, highly similar to Rab GDP dissociation inhibitor beta	38.3	91	32	22	8	30	
NQO1_HUMAN	NQO1	P15559	NAD(P)H dehydrogenase [quinone] 1 (EC 1.6.5.2) (Azoreductase) (DT-diap	38.3	91	5	3	60	24	Y
Q96MH4_HUMAN	Q96MH4_HUMAN	Q96MH4	cDNA FLJ32377 fis, clone SKMUS1000014, highly similar to Polyubiquitin	15.2	91	20	23	28	20	
TGM2_HUMAN	TGM2	P21980	Protein-glutamine gamma-glutamyltransferase 2 (EC 2.3.2.13) (Tissue tr	27.2	91	64	3	17	11	Y
PSMD1_HUMAN	PSMD1	Q99460	26S proteasome non-ATPase regulatory subunit 1 (26S proteasome regulat	19.1	90	23	1	43	27	Y
PAI1_HUMAN	SERPINE1	P05121	Plasminogen activator inhibitor 1 (PAI) (PAI-1) (Endothelial plasminog	38.6	90	43	4	23	25	Y
GFPT1_HUMAN	GFPT1	Q06210	Glutamine--fructose-6-phosphate aminotransferase [isomerizing] 1 (EC 2	24.7	90	35	10	23	22	Y
C9JZR2_HUMAN	CTNND1	C9JZR2	Catenin delta-1	20.1	88	30	21	8	29	Y
Q3B7A7_HUMAN	GART	Q3B7A7	Phosphoribosylglycinamide formyltransferase, phosphoribosylglycinamide	19.8	88	31	1	30	27	Y
TRFM_HUMAN	MFI2	P08582	Melanotransferrin (Melanoma-associated antigen p97) (CD antigen CD228)	19.4	87	23	19	1	46	Y
PPIB_HUMAN	PPIB	P23284	Peptidyl-prolyl cis-trans isomerase B (PPIase B) (EC 5.2.1.8) (CYP-S1)	41.7	87	15	33	28	10	Y
UBR4_HUMAN	UBR4	Q5T4S7	E3 ubiquitin-protein ligase UBR4 (EC 6.3.2.-) (600 kDa retinoblastoma	4.82	85	25	2	34	28	Y
NUCL_HUMAN	NCL	P19338	Nucleolin (Protein C23)	15.6	85	26	27	18	14	Y
VAT1_HUMAN	VAT1	Q99536	Synaptic vesicle membrane protein VAT-1 homolog (EC 1.-.-.-)	27.7	85	33	28	11	14	Y
Q53HV1_HUMAN	Q53HV1_HUMAN	Q53HV1	Ribosomal protein S4, X-linked X isoform variant (Fragment)	51.7	84	36	6	6	38	
AAAT_HUMAN	SLC1A5	Q15758	Neutral amino acid transporter B(0) (ATB(0)) (Baboon M7 virus receptor	16.1	84	24	9	18	33	Y
Q59GB4_HUMAN	Q59GB4_HUMAN	Q59GB4	Dihydropyrimidinase-like 2 variant (Fragment)	25.5	84	22	13	23	24	
Q53EM5_HUMAN	Q53EM5_HUMAN	Q53EM5	Transketolase variant (Fragment)	29.7	84	10	13	56	8	
B2R5B3_HUMAN	B2R5B3_HUMAN	B2R5B3	Histone H2A	40.8	83	17	11	14	42	
B2R5M8_HUMAN	B2R5M8_HUMAN	B2R5M8	Isocitrate dehydrogenase [NADP] (EC 1.1.1.42)	34.1	83	27	10	39	8	
SYNC_HUMAN	NARS	O43776	Asparagine--tRNA ligase, cytoplasmic (EC 6.1.1.22) (Asparaginyl-tRNA s	26.5	82	14	8	9	50	Y
GNAI1_HUMAN	GNAI1	P63096	Guanine nucleotide-binding protein G(i) subunit alpha-1 (Adenylate cyc	29.7	82	24	16	7	36	Y

Q6NXR8_HUMAN	RPS3A	Q6NXR8	40S ribosomal protein S3a	57.6	82	30	10	7	36	Y
GBLP_HUMAN	GNB2L1	P63244	Guanine nucleotide-binding protein subunit beta-2-like 1 (Cell prolife	44.2	80	25	8	18	32	Y
B4DR87_HUMAN	PLOD1	B4DR87	Procollagen-lysine,2-oxoglutarate 5-dioxygenase 1 (cDNA FLJ59393, high	23.5	80	33	10	8	30	Y
NCKP1_HUMAN	NCKAP1	Q9Y2A7	Nck-associated protein 1 (NAP 1) (Membrane-associated protein HEM-2) (	16.7	80	31	19	10	22	Y
RAB7A_HUMAN	RAB7A	P51149	Ras-related protein Rab-7a	55.1	80	26	23	13	18	Y
RRAS_HUMAN	RRAS	P10301	Ras-related protein R-Ras (p23)	35.3	79	18	21	10	32	Y
Q5JR95_HUMAN	RPS8	Q5JR95	40S ribosomal protein S8	49.5	79	30	11	7	32	Y
IPO7_HUMAN	IPO7	O95373	Importin-7 (Imp7) (Ran-binding protein 7) (RanBP7)	13.8	79	26	14	25	16	Y
A8K492_HUMAN	A8K492_HUMAN	A8K492	cDNA FLJ76789, highly similar to Homo sapiens methionine-tRNA syntheta	14.3	78	25	5	21	29	
B7Z6Z4_HUMAN	MYL6	B7Z6Z4	Myosin light polypeptide 6 (cDNA FLJ56329, highly similar to Myosin li	32.4	78	33	13	10	21	Y
PRS10_HUMAN	PSMC6	P62333	26S protease regulatory subunit 10B (26S proteasome AAA-ATPase subunit	32.9	77	18	8	21	34	Y
CO6A1_HUMAN	COL6A1	P12109	Collagen alpha-1(VI) chain	13.5	77	40	12	2	23	Y
B2R9K8_HUMAN	B2R9K8_HUMAN	B2R9K8	cDNA, FLJ94440, highly similar to Homo sapiens chaperonin containing T	26	77	15	6	37	20	
E41L2_HUMAN	EPB41L2	O43491	Band 4.1-like protein 2 (Generally expressed protein 4.1) (4.1G)	19	76	36	4	1	37	Y
LPPRC_HUMAN	LRPPRC	P42704	Leucine-rich PPR motif-containing protein, mitochondrial (130 kDa leuc	19.9	76	19	2	26	30	Y
RAB8A_HUMAN	RAB8A	P61006	Ras-related protein Rab-8A (Oncogene c-mel)	35.3	75	24	15	9	29	Y
B4DUQ1_HUMAN	B4DUQ1_HUMAN	B4DUQ1	cDNA FLJ54552, highly similar to Heterogeneous nuclear ribonucleoprote	28.5	75	17	11	23	25	
A8K9V7_HUMAN	A8K9V7_HUMAN	A8K9V7	cDNA FLJ77207	16.1	75	32	11	9	25	
B2R657_HUMAN	B2R657_HUMAN	B2R657	Annexin	22.5	75	20	15	20	23	
AP2A2_HUMAN	AP2A2	O94973	AP-2 complex subunit alpha-2 (100 kDa coated vesicle protein C) (Adapt	20.7	75	24	18	18	17	Y
RAB1A_HUMAN	RAB1A	P62820	Ras-related protein Rab-1A (YPT1-related protein)	40.5	75	21	25	16	15	Y
Q53HW2_HUMAN	Q53HW2_HUMAN	Q53HW2	Ribosomal protein P0 variant (Fragment)	27.1	74	17	12	20	25	
SERPH_HUMAN	SERPINH1	P50454	Serpin H1 (47 kDa heat shock protein) (Arsenic-transactivated protein	31.1	74	30	33	2	7	Y
B4DGU4_HUMAN	CTNNB1	B4DGU4	Catenin beta-1 (cDNA FLJ57878, highly similar to Beta-catenin)	21.3	73	5	31	6	31	Y
B5MDF5_HUMAN	RAN	B5MDF5	GTP-binding nuclear protein Ran (RAN, member RAS oncogene family, isof	30	73	21	7	20	26	Y
RAB2A_HUMAN	RAB2A	P61019	Ras-related protein Rab-2A	59	73	27	16	12	18	Y
HSPB1_HUMAN	HSPB1	P04792	Heat shock protein beta-1 (HspB1) (28 kDa heat shock protein) (Estroge	52.7	73	22	14	23	17	Y

6PGD_HUMAN	PGD	P52209	6-phosphogluconate dehydrogenase, decarboxylating (EC 1.1.1.44)	29.4	73	10	9	44	11	Y
A8K9A4_HUMAN	A8K9A4_HUMAN	A8K9A4	cDNA FLJ75154, highly similar to Homo sapiens heterogeneous nuclear ri	26.5	72	20	10	17	28	
GBB2_HUMAN	GNB2	P62879	Guanine nucleotide-binding protein G(I)/G(S)/G(T) subunit beta-2 (G pr	27.6	72	23	18	9	24	Y
B3KSH1_HUMAN	EIF3F	B3KSH1	Eukaryotic translation initiation factor 3 subunit F (eIF3f) (Eukaryot	25.8	72	23	7	22	20	Y
TAGL2_HUMAN	TAGLN2	P37802	Transgelin-2 (Epididymis tissue protein Li 7e) (SM22-alpha homolog)	51.3	72	30	8	17	17	Y
COF1_HUMAN	CFL1	P23528	Cofilin-1 (18 kDa phosphoprotein) (p18) (Cofilin, non-muscle isoform)	49.4	72	24	19	15	14	Y
PP1A_HUMAN	PPP1CA	P62136	Serine/threonine-protein phosphatase PP1-alpha catalytic subunit (PP-1	30	71	18	13	9	32	Y
SAHH_HUMAN	AHCY	P23526	Adenosylhomocysteinase (AdoHcyase) (EC 3.3.1.1) (S-adenosyl-L-homocyst	30.6	71	18	5	25	24	Y
B1ANR0_HUMAN	PABPC4	B1ANR0	Poly(A) binding protein, cytoplasmic 4 (Inducible form), isoform CRA_e	17.2	71	26	8	15	23	Y
Q9BU08_HUMAN	Q9BU08_HUMAN	Q9BU08	Putative uncharacterized protein (Fragment)	26.5	71	18	5	32	18	
PSB3_HUMAN	PSMB3	P49720	Proteasome subunit beta type-3 (EC 3.4.25.1) (Proteasome chain 13) (Pr	37.6	71	19	7	34	11	Y
Q53HS0_HUMAN	Q53HS0_HUMAN	Q53HS0	Glutaminyl-tRNA synthetase variant (Fragment)	26.6	70	13	5	21	36	
RS16_HUMAN	RPS16	P62249	40S ribosomal protein S16	60.3	70	23	8	11	29	Y
B4E1D5_HUMAN	EIF3C	B4E1D5	Eukaryotic translation initiation factor 3 subunit C (eIF3c) (Eukaryot	11.3	70	25	12	14	21	Y
DHX15_HUMAN	DHX15	O43143	Putative pre-mRNA-splicing factor ATP-dependent RNA helicase DHX15 (EC	21.9	69	12	2	15	41	Y
GNA11_HUMAN	GNA11	P29992	Guanine nucleotide-binding protein subunit alpha-11 (G alpha-11) (G-pr	30.1	69	14	11	3	41	Y
Q8TBK5_HUMAN	RPL6	Q8TBK5	60S ribosomal protein L6	44.4	69	22	13	7	27	Y
COPG1_HUMAN	COPG1	Q9Y678	Coatomer subunit gamma-1 (Gamma-1-coat protein) (Gamma-1-COP)	19.3	69	24	9	21	15	Y
PSA_HUMAN	NPEPPS	P55786	Puromycin-sensitive aminopeptidase (PSA) (EC 3.4.11.14) (Cytosol alany	23.7	69	27	11	26	7	Y
Q53FT8_HUMAN	Q53FT8_HUMAN	Q53FT8	Proteasome subunit beta type (EC 3.4.25.1) (Fragment)	36.5	69	17	7	43	5	
ABCE1_HUMAN	ABCE1	P61221	ATP-binding cassette sub-family E member 1 (2'-5'-oligoadenylate-bindi	33.2	68	12	3	17	38	Y
PP1B_HUMAN	PPP1CB	P62140	Serine/threonine-protein phosphatase PP1-beta catalytic subunit (PP-1B	25.1	68	18	11	10	31	Y
RL7A_HUMAN	RPL7A	P62424	60S ribosomal protein L7a (PLA-X polypeptide) (Surfeit locus protein 3	25.6	68	24	5	11	29	Y
C9IZE4_HUMAN	PSMD6	C9IZE4	26S proteasome non-ATPase regulatory subunit 6 (Proteasome (Prosome, m	36	68	18	6	17	28	Y
CBR1_HUMAN	CBR1	P16152	Carbonyl reductase [NADPH] 1 (EC 1.1.1.184) (15-hydroxyprostaglandin d	47.3	68	22	6	19	22	Y
AMPL_HUMAN	LAP3	P28838	Cytosol aminopeptidase (EC 3.4.11.1) (Leucine aminopeptidase 3) (LAP-3	41.4	68	52	4	3	9	Y

EHD2_HUMAN	EHD2	Q9NZN4	EH domain-containing protein 2 (PAST homolog 2)	30.9	67	11	20	8	31	Y
H0YNE9_HUMAN	RAB8B	H0YNE9	Ras-related protein Rab-8B (Fragment)	38.8	67	23	17	4	25	Y
Q53GX7_HUMAN	Q53GX7_HUMAN	Q53GX7	Threonyl-tRNA synthetase variant (Fragment)	18.1	67	26	7	22	13	
GNA13_HUMAN	GNA13	Q14344	Guanine nucleotide-binding protein subunit alpha-13 (G alpha-13) (G-pr	30.2	66	31	12	4	20	Y
NAMPT_HUMAN	NAMPT	P43490	Nicotinamide phosphoribosyltransferase (NAmPRTase) (Nampt) (EC 2.4.2.1	29.5	66	25	4	35	5	Y
PRS4_HUMAN	PSMC1	P62191	26S protease regulatory subunit 4 (P26s4) (26S proteasome AAA-ATPase s	22.3	65	19	1	18	29	Y
RALA_HUMAN	RALA	P11233	Ras-related protein Ral-A	26.2	65	18	17	3	28	Y
B2RD27_HUMAN	B2RD27_HUMAN	B2RD27	cDNA, FLJ96428, highly similar to Homo sapiens proteasome (prosome, ma	24.1	65	23	3	17	21	
Q53HV2_HUMAN	Q53HV2_HUMAN	Q53HV2	Chaperonin containing TCP1, subunit 7 (Eta) variant (Fragment)	29.3	65	17	6	29	19	
Q5T5C7_HUMAN	SARS	Q5T5C7	Serine--tRNA ligase, cytoplasmic	24.3	64	28	1	4	34	Y
Q9Y6E3_HUMAN	Q9Y6E3_HUMAN	Q9Y6E3	HSPC027	33.2	64	21	2	21	23	
Q53HH4_HUMAN	Q53HH4_HUMAN	Q53HH4	Ras-GTPase-activating protein SH3-domain-binding protein variant (Frag	18.2	64	17	9	19	20	
AT2B4_HUMAN	ATP2B4	P23634	Plasma membrane calcium-transporting ATPase 4 (PMCA4) (EC 3.6.3.8) (Ma	11	64	17	23	4	20	Y
D3DPU2_HUMAN	CAP1	D3DPU2	Adenylyl cyclase-associated protein	33.3	64	24	12	14	18	Y
TPIS_HUMAN	TPI1	P60174	Triosephosphate isomerase (TIM) (EC 5.3.1.1) (Triose-phosphate isomera	49.3	64	30	6	12	18	Y
Q6FIG4_HUMAN	RAB1B	Q6FIG4	RAB1B protein	40.3	64	17	21	10	16	Y
EFTU_HUMAN	TUFM	P49411	Elongation factor Tu, mitochondrial (EF-Tu) (P43)	33.8	63	15	2	13	37	Y
RAB10_HUMAN	RAB10	P61026	Ras-related protein Rab-10	49.5	63	17	17	8	21	Y
B4DE59_HUMAN	B4DE59_HUMAN	B4DE59	cDNA FLJ60424, highly similar to Junction plakoglobin	29.7	63	20	26	13	7	
ILF2_HUMAN	ILF2	Q12905	Interleukin enhancer-binding factor 2 (Nuclear factor of activated T-c	23.1	62	13	4	20	29	Y
2AAA_HUMAN	PPP2R1A	P30153	Serine/threonine-protein phosphatase 2A 65 kDa regulatory subunit A al	22.6	62	17	7	19	20	Y
Q59ER5_HUMAN	Q59ER5_HUMAN	Q59ER5	WD repeat-containing protein 1 isoform 1 variant (Fragment)	13.5	62	24	10	13	15	
G3V5Z7_HUMAN	PSMA6	G3V5Z7	Proteasome subunit alpha type (EC 3.4.25.1)	42.5	62	15	11	36	1	Y
RS2_HUMAN	RPS2	P15880	40S ribosomal protein S2 (40S ribosomal protein S4) (Protein LLRep3)	39.9	61	20	6	6	34	Y
SERA_HUMAN	PHGDH	O43175	D-3-phosphoglycerate dehydrogenase (3-PGDH) (EC 1.1.1.95)	23.3	61	22	2	8	31	Y
B4DY23_HUMAN	B4DY23_HUMAN	B4DY23	cDNA FLJ61188, highly similar to Basigin	35.1	61	24	17	3	17	

B3KU62_HUMAN	B3KU62_HUMAN	B3KU62	cDNA FLJ39243 fis, clone OCBBF2008283, highly similar to Protein NDRG1	22.2	61	41	6	1	13	
A5YM53_HUMAN	ITGAV	A5YM53	ITGAV protein	13.8	60	11	10	12	27	Y
B3KTA3_HUMAN	B3KTA3_HUMAN	B3KTA3	Fascin	25.6	60	17	6	11	27	
A4D210_HUMAN	EIF3B	A4D210	Eukaryotic translation initiation factor 3 subunit B (eIF3b) (Eukaryot	16.3	60	17	6	17	21	Y
LEG1_HUMAN	LGALS1	P09382	Galectin-1 (Gal-1) (14 kDa laminin-binding protein) (HLBP14) (14 kDa l	54.1	60	21	12	7	21	Y
E9PCY7_HUMAN	HNRNPH1	E9PCY7	Heterogeneous nuclear ribonucleoprotein H	19.8	60	16	10	16	19	Y
1433Z_HUMAN	YWHAZ	P63104	14-3-3 protein zeta/delta (Protein kinase C inhibitor protein 1) (KCIP	46.5	60	25	15	9	13	Y
Q59GL1_HUMAN	Q59GL1_HUMAN	Q59GL1	Synaptotagmin binding, cytoplasmic RNA interacting protein variant (Fr	26	59	14	3	12	32	
B4E1G2_HUMAN	B4E1G2_HUMAN	B4E1G2	Serine hydroxymethyltransferase (EC 2.1.2.1)	31.4	59	13	3	13	30	
RL7_HUMAN	RPL7	P18124	60S ribosomal protein L7	34.3	59	21	10	4	27	Y
B2RDW0_HUMAN	B2RDW0_HUMAN	B2RDW0	cDNA, FLJ96792, highly similar to Homo sapiens calmodulin 2 (phosphory	40.9	59	11	14	11	24	
IF2G_HUMAN	EIF2S3	P41091	Eukaryotic translation initiation factor 2 subunit 3 (Eukaryotic trans	30.7	59	32	1	12	16	Y
ACADV_HUMAN	ACADVL	P49748	Very long-chain specific acyl-CoA dehydrogenase, mitochondrial (VLCAD)	30.5	58	8	8	10	35	Y
A8K4T6_HUMAN	A8K4T6_HUMAN	A8K4T6	cDNA FLJ76282, highly similar to Homo sapiens proteasome (prosome, mac	19.2	58	16	4	16	23	
ACTZ_HUMAN	ACTR1A	P61163	Alpha-centractin (Centractin) (ARP1) (Actin-RPV) (Centrosome-associate	16.8	58	15	10	19	15	Y
PUR6_HUMAN	PAICS	P22234	Multifunctional protein ADE2 [Includes: Phosphoribosylaminoimidazole-s	28.2	58	27	3	17	14	Y
B2R7W4_HUMAN	B2R7W4_HUMAN	B2R7W4	cDNA, FLJ93632, highly similar to Homo sapiens heterogeneous nuclear r	24.5	57	16	2	8	34	
A4D2P0_HUMAN	RAC1	A4D2P0	Ras-related C3 botulinum toxin substrate 1 (Rho family, small GTP bind	35.5	57	21	13	3	20	Y
D3DRX6_HUMAN	KIF5B	D3DRX6	Kinesin family member 5B, isoform CRA_a	21.5	57	8	5	25	19	Y
RAB14_HUMAN	RAB14	P61106	Ras-related protein Rab-14	38.6	57	18	13	10	17	Y
RL10A_HUMAN	RPL10A	P62906	60S ribosomal protein L10a (CSA-19) (Neural precursor cell expressed d	33.2	56	16	2	16	27	Y
Q6ZS74_HUMAN	RALB	Q6ZS74	Ras-related protein Ral-B (V-ral simian leukemia viral oncogene homolo	24.2	56	17	15	3	22	Y
AT2B1_HUMAN	ATP2B1	P20020	Plasma membrane calcium-transporting ATPase 1 (PMCA1) (EC 3.6.3.8) (Pl	11.4	56	22	13	4	17	Y
CD166_HUMAN	ALCAM	Q13740	CD166 antigen (Activated leukocyte cell adhesion molecule) (CD antigen	13.9	55	17	13	4	21	Y
1433B_HUMAN	YWHAB	P31946	14-3-3 protein beta/alpha (Protein 1054) (Protein kinase C inhibitor p	38.2	55	23	11	3	19	Y
MDHM_HUMAN	MDH2	P40926	Malate dehydrogenase, mitochondrial (EC 1.1.1.37)	39.1	55	21	5	13	17	Y

RAB5C_HUMAN	RAB5C	P51148	Ras-related protein Rab-5C (L1880) (RAB5L)	38.9	55	25	17	5	11	Y
PRDX6_HUMAN	PRDX6	P30041	Peroxiredoxin-6 (EC 1.11.1.15) (1-Cys peroxiredoxin) (1-Cys PRX) (24 k	46.4	55	36	12	6	8	Y
B3KY95_HUMA N	B3KY95_HUMA N	B3KY95	cDNA FLJ16143 fis, clone BRAMY2038516, highly similar to Protein disul	23.4	55	27	15	8	4	
B7Z5Z2_HUMAN	RRAS2	B7Z5Z2	Ras-related protein R-Ras2 (cDNA FLJ61162, highly similar to Ras-relat	41.4	54	18	20	2	16	Y
ITA2_HUMAN	ITGA2	P17301	Integrin alpha-2 (CD49 antigen-like family member B) (Collagen recepto	14.4	53	19	9	2	24	Y
IF4A3_HUMAN	EIF4A3	P38919	Eukaryotic initiation factor 4A-III (eIF-4A-III) (eIF4A-III) (EC 3.6.4	28.5	53	9	4	16	23	Y
HNRPF_HUMAN	HNRNPF	P52597	Heterogeneous nuclear ribonucleoprotein F (hnRNP F) (Nucleolin-like pr	24.6	53	14	11	12	18	Y
B7Z9C2_HUMAN	NAP1L1	B7Z9C2	Nucleosome assembly protein 1-like 1 (cDNA FLJ58569, highly similar to	18.3	53	16	14	10	13	Y
PGAM1_HUMAN	PGAM1	P18669	Phosphoglycerate mutase 1 (EC 3.1.3.13) (EC 5.4.2.11) (EC 5.4.2.4) (BP	25.2	52	18	5	10	19	Y
CTL1_HUMAN	SLC44A1	Q8WWI5	Choline transporter-like protein 1 (CDw92) (Solute carrier family 44 m	12.6	52	13	10	11	18	Y
Q32Q12_HUMAN	NME1-NME2	Q32Q12	Nucleoside diphosphate kinase (EC 2.7.4.6)	31.5	52	20	11	13	11	Y
PARP4_HUMAN	PARP4	Q9UKK3	Poly [ADP-ribose] polymerase 4 (PARP-4) (EC 2.4.2.30) (193 kDa vault p	11.4	52	6	4	40	1	Y
DDX17_HUMAN	DDX17	Q92841	Probable ATP-dependent RNA helicase DDX17 (EC 3.6.4.13) (DEAD box prot	16.9	51	12	2	15	22	Y
CN37_HUMAN	CNP	P09543	2',3'-cyclic-nucleotide 3'-phosphodiesterase (CNP) (CNPase) (EC 3.1.4.	22.8	51	28	7	1	15	Y
Q9BTQ7_HUMA N	Q9BTQ7_HUMA N	Q9BTQ7	Similar to ribosomal protein L23 (Fragment)	34.3	51	18	14	6	14	
UBP5_HUMAN	USP5	P45974	Ubiquitin carboxyl-terminal hydrolase 5 (EC 3.4.19.12) (Deubiquitinati	12.2	51	18	6	19	8	Y
MCM5_HUMAN	MCM5	P33992	DNA replication licensing factor MCM5 (EC 3.6.4.12) (CDC46 homolog) (P	26.3	50	4	5	6	35	Y
CD82_HUMAN	CD82	P27701	CD82 antigen (C33 antigen) (IA4) (Inducible membrane protein R2) (Meta	16.9	50	15	11	3	22	Y
A8K666_HUMAN	A8K666_HUMA N	A8K666	cDNA FLJ75460, highly similar to Homo sapiens phenylalanine-tRNA synth	20.2	50	10	4	15	21	
DHE3_HUMAN	GLUD1	P00367	Glutamate dehydrogenase 1, mitochondrial (GDH 1) (EC 1.4.1.3)	18.1	50	3	1	29	18	Y
RB11B_HUMAN	RAB11B	Q15907	Ras-related protein Rab-11B (GTP-binding protein YPT3)	44.5	50	13	16	5	18	Y
DX39B_HUMAN	DDX39B	Q13838	Spliceosome RNA helicase DDX39B (EC 3.6.4.13) (56 kDa U2AF65-associate	22.7	50	22	4	11	14	Y
GFPT2_HUMAN	GFPT2	O94808	Glutamine--fructose-6-phosphate aminotransferase [isomerizing] 2 (EC 2	16.3	50	32	2	6	11	Y
TS101_HUMAN	TSG101	Q99816	Tumor susceptibility gene 101 protein (ESCRT-I complex subunit TSG101)	25.1	50	6	12	26	8	Y
B7Z2N4_HUMA N	B7Z2N4_HUMA N	B7Z2N4	cDNA FLJ56074, highly similar to 150 kDa oxygen-regulated protein (Orp	19.1	50	41	1	2	6	
PLOD2_HUMAN	PLOD2	O00469	Procollagen-lysine,2-oxoglutarate 5-dioxygenase 2 (EC 1.14.11.4) (Lysy	19.7	50	25	17	7	2	Y
RTCB_HUMAN	RTCB	Q9Y3I0	tRNA-splicing ligase RtcB homolog (EC 6.5.1.3)	25	49	13	1	11	25	

RS24_HUMAN	RPS24	P62847	40S ribosomal protein S24	29.3	49	12	11	9	19	Y
E7EUU4_HUMAN	EIF4G1	E7EUU4	Eukaryotic translation initiation factor 4 gamma 1	9.81	49	27	1	8	15	Y
ARP2_HUMAN	ACTR2	P61160	Actin-related protein 2 (Actin-like protein 2)	21.8	49	11	14	11	14	Y
B3KVN0_HUMAN	B3KVN0_HUMAN	B3KVN0	cDNA FLJ16785 fis, clone NT2RI2015342, highly similar to Solute carrie	9.13	49	26	3	7	13	
Q59EF6_HUMAN	Q59EF6_HUMAN	Q59EF6	Calpain 2, large [catalytic] subunit variant (Fragment)	19.5	48	12	1	2	33	
J3KN16_HUMAN	KIAA0368	J3KN16	Proteasome-associated protein ECM29 homolog	7.78	48	12	2	15	21	Y
APT_HUMAN	APRT	P07741	Adenine phosphoribosyltransferase (APRT) (EC 2.4.2.7)	57.2	48	13	5	12	20	Y
FLII_HUMAN	FLII	Q13045	Protein flightless-1 homolog	11	48	12	5	12	19	Y
B2R6K4_HUMAN	B2R6K4_HUMAN	B2R6K4	cDNA, FLJ92996, highly similar to Homo sapiens guanine nucleotide bind	22.1	48	14	13	7	15	
A8K3C3_HUMAN	A8K3C3_HUMAN	A8K3C3	T-complex protein 1 subunit delta	20.4	48	12	10	14	12	
B4E175_HUMAN	B4E175_HUMAN	B4E175	cDNA FLJ54775, highly similar to Syntaxin-binding protein 2	21.5	48	25	16	1	5	
RS9_HUMAN	RPS9	P46781	40S ribosomal protein S9	41.2	47	17	4	2	26	Y
FERM2_HUMAN	FERMT2	Q96AC1	Fermitin family homolog 2 (Kindlin-2) (Mitogen-inducible gene 2 protei	19	47	14	9	1	23	Y
RL10_HUMAN	RPL10	P27635	60S ribosomal protein L10 (Laminin receptor homolog) (Protein QM) (Tum	15.4	47	19	6	2	22	Y
RS15A_HUMAN	RPS15A	P62244	40S ribosomal protein S15a	36.9	47	14	7	10	17	Y
E7ES43_HUMAN	HSPA4L	E7ES43	Heat shock 70 kDa protein 4L	8.28	47	12	6	17	13	Y
ARF1_HUMAN	ARF1	P84077	ADP-ribosylation factor 1	37	47	14	9	11	13	Y
SP16H_HUMAN	SUPT16H	Q9Y5B9	FACT complex subunit SPT16 (Chromatin-specific transcription elongatio	15	46	10	1	9	28	Y
B0QZ18_HUMAN	CPNE1	B0QZ18	Copine-1 (HCG38213, isoform CRA_b)	15.5	46	14	4	4	25	Y
A8K6Y1_HUMAN	A8K6Y1_HUMAN	A8K6Y1	cDNA FLJ75526, highly similar to Homo sapiens proliferation-associated	32.7	46	13	1	17	16	
C9JV86_HUMAN	CD63	C9JV86	CD63 antigen	5.08	46	10	12	12	12	Y
B2RWP9_HUMAN	MYH10	B2RWP9	MYH10 protein	4.79	45	21	11	2	15	Y
B5BTY4_HUMAN	DDX3X	B5BTY4	ATP-dependent RNA helicase DDX3X	19	45	17	6	10	13	Y
1433G_HUMAN	YWHAG	P61981	14-3-3 protein gamma (Protein kinase C inhibitor protein 1) (KCIP-1) [	32	45	19	11	4	12	Y
SEPT2_HUMAN	Sep-02	Q15019	Septin-2 (Neural precursor cell expressed developmentally down-regulat	26.3	45	10	15	10	10	Y
ARP3_HUMAN	ACTR3	P61158	Actin-related protein 3 (Actin-like protein 3)	16.5	45	21	8	5	10	Y
PSA5_HUMAN	PSMA5	P28066	Proteasome subunit alpha type-5 (EC 3.4.25.1) (Macropain zeta chain) (	29.9	45	12	1	25	7	Y

B7Z5J7_HUMAN	B7Z5J7_HUMAN	B7Z5J7	cDNA FLJ58682, highly similar to Vesicle-fusing ATPase (EC 3.6.4.6)	18.4	44	17	9	7	13	
RL12_HUMAN	RPL12	P30050	60S ribosomal protein L12	33.9	44	10	9	16	11	Y
H7C410_HUMAN	GPC1	H7C410	Glypican-1 (Fragment)	12.4	44	11	9	18	7	Y
ACACA_HUMAN	ACACA	Q13085	Acetyl-CoA carboxylase 1 (ACC1) (EC 6.4.1.2) (ACC-alpha) [Includes: Bi	9.42	44	8	1	31	4	Y
PSB5_HUMAN	PSMB5	P28074	Proteasome subunit beta type-5 (EC 3.4.25.1) (Macropain epsilon chain)	35.7	44	5	3	34	2	Y
SYYC_HUMAN	YARS	P54577	Tyrosine--tRNA ligase, cytoplasmic (EC 6.1.1.1) (Tyrosyl-tRNA syntheta	28.2	43	11	2	9	24	Y
ETFA_HUMAN	ETFA	P13804	Electron transfer flavoprotein subunit alpha, mitochondrial (Alpha-ETF	24.3	43	4	5	17	17	Y
PRS8_HUMAN	PSMC5	P62195	26S protease regulatory subunit 8 (26S proteasome AAA-ATPase subunit R	28.8	43	17	1	11	15	Y
RL5_HUMAN	RPL5	P46777	60S ribosomal protein L5	34	43	16	6	6	15	Y
PRS6B_HUMAN	PSMC4	P43686	26S protease regulatory subunit 6B (26S proteasome AAA-ATPase subunit	17.7	43	12	5	17	9	Y
PSA7_HUMAN	PSMA7	O14818	Proteasome subunit alpha type-7 (EC 3.4.25.1) (Proteasome subunit RC6-	29.8	43	8	6	22	7	Y
DSG2_HUMAN	DSG2	Q14126	Desmoglein-2 (Cadherin family member 5) (HDGC)	10.6	42	4	13	3	22	Y
D3DV26_HUMAN	S100A10	D3DV26	S100 calcium binding protein A10 (Annexin II ligand, calpactin I, ligh	15.6	42	12	9	9	13	Y
RS7_HUMAN	RPS7	P62081	40S ribosomal protein S7	22.7	42	12	8	9	13	Y
VIGLN_HUMAN	HDLBP	Q00341	Vigilin (High density lipoprotein-binding protein) (HDL-binding protei	11.4	42	8	4	18	12	Y
J3KQ32_HUMAN	OLA1	J3KQ32	Obg-like ATPase 1	16.1	42	13	7	12	10	Y
ACOC_HUMAN	ACO1	P21399	Cytoplasmic aconitate hydratase (Aconitase) (EC 4.2.1.3) (Citrate hydr	7.65	41	19	6	6	10	Y
S10AB_HUMAN	S100A11	P31949	Protein S100-A11 (Calgizzarin) (Metastatic lymph node gene 70 protein)	41	41	21	4	7	9	Y
B2RDE1_HUMAN	B2RDE1_HUMAN	B2RDE1	cDNA, FLJ96568, highly similar to Homo sapiens tropomyosin 3 (TPM3), m	28.6	41	28	4	3	6	
FKBP4_HUMAN	FKBP4	Q02790	Peptidyl-prolyl cis-trans isomerase FKBP4 (PPIase FKBP4) (EC 5.2.1.8)	15.7	41	10	6	20	5	Y
B7Z1R5_HUMAN	ATP6V1A	B7Z1R5	V-type proton ATPase catalytic subunit A (cDNA FLJ51804, highly simila	15.4	41	17	9	9	5	Y
PSA4_HUMAN	PSMA4	P25789	Proteasome subunit alpha type-4 (EC 3.4.25.1) (Macropain subunit C9) (	26.8	41	8	3	26	4	Y
PSA3_HUMAN	PSMA3	P25788	Proteasome subunit alpha type-3 (EC 3.4.25.1) (Macropain subunit C8) (	35.3	41	10	3	27	1	Y
A8K590_HUMAN	A8K590_HUMAN	A8K590	cDNA FLJ77456, highly similar to Homo sapiens interleukin enhancer bin	20.5	40	9	6	6	21	
DIP2B_HUMAN	DIP2B	Q9P265	Disco-interacting protein 2 homolog B (DIP2 homolog B)	9.2	40	10	8	6	16	Y
TNPO1_HUMAN	TNPO1	Q92973	Transportin-1 (Importin beta-2) (Karyopherin beta-2) (M9 region intera	15.8	40	21	4	7	13	Y
A8K9P0_HUMAN	A8K9P0_HUMAN	A8K9P0	cDNA FLJ78413, highly similar to Homo sapiens albumin, mRNA	8.88	40	9	9	11	11	

SEPT9_HUMAN	Sep-09	Q9UHD8	Septin-9 (MLL septin-like fusion protein MSF-A) (MLL septin-like fusio	14.8	40	12	4	15	8	Y
MAP1B_HUMAN	MAP1B	P46821	Microtubule-associated protein 1B (MAP-1B) [Cleaved into: MAP1B heavy	7.29	40	10	2	24	5	Y
RLA2_HUMAN	RPLP2	P05387	60S acidic ribosomal protein P2 (Renal carcinoma antigen NY-REN-44)	55.7	39	12	5	5	17	Y
E9PN11_HUMAN	RHOC	E9PN11	Rho-related GTP-binding protein RhoC (Fragment)	25.3	39	11	12	1	15	Y
ATPA_HUMAN	ATP5A1	P25705	ATP synthase subunit alpha, mitochondrial	22.1	39	2	7	16	14	Y
INF2_HUMAN	INF2	Q27J81	Inverted formin-2 (HBEBP2-binding protein C)	7.61	39	13	3	10	13	Y
B2R983_HUMAN	B2R983_HUMAN	B2R983	cDNA, FLJ94267, highly similar to Homo sapiens glutathione S-transfera	27	39	16	3	9	11	
FLOT1_HUMAN	FLOT1	O75955	Flotillin-1	23.4	39	6	14	11	8	Y
RINI_HUMAN	RNH1	P13489	Ribonuclease inhibitor (Placental ribonuclease inhibitor) (Placental R	12.8	39	10	13	8	8	Y
PSME3_HUMAN	PSME3	P61289	Proteasome activator complex subunit 3 (11S regulator complex subunit	26.4	39	16	2	14	7	Y
AL7A1_HUMAN	ALDH7A1	P49419	Alpha-aminoadipic semialdehyde dehydrogenase (Alpha-AASA dehydrogenase	25.2	38	4	3	7	25	Y
HCD2_HUMAN	HSD17B10	Q99714	3-hydroxyacyl-CoA dehydrogenase type-2 (EC 1.1.1.35) (17-beta-hydroxys	43.3	38	9	1	9	20	Y
J3KRX5_HUMAN	RPL17	J3KRX5	60S ribosomal protein L17 (Fragment)	34.5	38	13	7	4	16	Y
B2R5W3_HUMAN	B2R5W3_HUMAN	B2R5W3	cDNA, FLJ92658, highly similar to Homo sapiens poly (ADP-ribose) polym	10.8	38	10	4	10	15	
HPRT_HUMAN	HPRT1	P00492	Hypoxanthine-guanine phosphoribosyltransferase (HGPRT) (HGPRTase) (EC	38.1	38	19	5	3	12	Y
MOT4_HUMAN	SLC16A3	O15427	Monocarboxylate transporter 4 (MCT 4) (Solute carrier family 16 member	17	38	17	8	2	11	Y
HS105_HUMAN	HSPH1	Q92598	Heat shock protein 105 kDa (Antigen NY-CO-25) (Heat shock 110 kDa prot	16.7	38	17	11	6	5	Y
RS10_HUMAN	RPS10	P46783	40S ribosomal protein S10	23.6	37	15	2	6	16	Y
G5E948_HUMAN	MAP4K4	G5E948	Mitogen-activated protein kinase kinase kinase kinase 4 (Mitogen-activ	6.67	37	9	12	3	14	Y
CPNE3_HUMAN	CPNE3	O75131	Copine-3 (Copine III)	16.8	37	12	8	3	14	Y
PSD12_HUMAN	PSMD12	O00232	26S proteasome non-ATPase regulatory subunit 12 (26S proteasome regula	23	37	13	2	13	9	Y
B4DZI8_HUMAN	COPB2	B4DZI8	Coatomer protein complex, subunit beta 2 (Beta prime), isoform CRA_b (	11.1	37	7	5	18	8	Y
B2R6J4_HUMAN	B2R6J4_HUMAN	B2R6J4	cDNA, FLJ92975, highly similar to Homo sapiens nucleosome assembly pro	17.3	37	9	8	12	8	
COR1C_HUMAN	CORO1C	Q9ULV4	Coronin-1C (Coronin-3) (hCRNN4)	11	37	9	13	6	8	Y
A3KC71_HUMAN	okuribin	A3KC71	Nuclear envelope protein okuribin	11.7	37	5	21	4	7	
PSA1_HUMAN	PSMA1	P25786	Proteasome subunit alpha type-1 (EC 3.4.25.1) (30 kDa prosomal protein	38	37	9	2	24	4	Y
KIF23_HUMAN	KIF23	Q02241	Kinesin-like protein KIF23 (Kinesin-like protein 5) (Mitotic kinesin-l	13.5	37	16	9	8	3	Y

GALE_HUMAN	GALE	Q14376	UDP-glucose 4-epimerase (EC 5.1.3.2) (Galactowaldenase) (UDP-galactose	16.7	36	20	1	3	15	Y
J3KTA4_HUMAN	DDX5	J3KTA4	Probable ATP-dependent RNA helicase DDX5	12.5	36	12	2	8	14	Y
B4DJA5_HUMAN	RAB5A	B4DJA5	Ras-related protein Rab-5A (cDNA FLJ51867, highly similar to Ras-relat	35.8	36	16	6	4	9	Y
Q6DEN2_HUMAN	DPYSL3	Q6DEN2	DPYSL3 protein	23.1	35	2	6	1	28	Y
B4DNN4_HUMAN	B4DNN4_HUMAN	B4DNN4	Ribonucleoside-diphosphate reductase (EC 1.17.4.1)	13	35	3	3	13	17	
J3QQ67_HUMAN	RPL18	J3QQ67	60S ribosomal protein L18 (Fragment)	40.5	35	13	6	3	15	Y
B4DV28_HUMAN	B4DV28_HUMAN	B4DV28	cDNA FLJ54170, highly similar to Cytosolic nonspecific dipeptidase	16.8	35	13	7	5	10	
SPB6_HUMAN	SERPINB6	P35237	Serpin B6 (Cytoplasmic antiproteinase) (CAP) (Peptidase inhibitor 6) (	22.1	35	13	10	1	10	Y
Q6NVW7_HUMAN	KPNA2	Q6NVW7	Importin subunit alpha	19.8	35	9	3	14	9	Y
PDXK_HUMAN	PDXK	O00764	Pyridoxal kinase (EC 2.7.1.35) (Pyridoxine kinase)	24.7	35	3	4	20	8	Y
E7EUC7_HUMAN	UGP2	E7EUC7	UTP--glucose-1-phosphate uridylyltransferase	13	35	13	10	10	2	Y
J3KTL2_HUMAN	SRSF1	J3KTL2	Serine/arginine-rich-splicing factor 1 (Splicing factor, arginine/seri	30	34	10	2	1	21	Y
ROA2_HUMAN	HNRNPA2B1	P22626	Heterogeneous nuclear ribonucleoproteins A2/B1 (hnRNP A2/B1)	25.5	34	11	2	6	15	Y
B1AK87_HUMAN	CAPZB	B1AK87	Capping protein (Actin filament) muscle Z-line, beta, isoform CRA_a (F	23.5	34	17	5	5	7	Y
KAP0_HUMAN	PRKAR1A	P10644	cAMP-dependent protein kinase type I-alpha regulatory subunit (Tissue-	23.9	33	8	7	4	16	Y
PRPS2_HUMAN	PRPS2	P11908	Ribose-phosphate pyrophosphokinase 2 (EC 2.7.6.1) (PPRibP) (Phosphorib	39	33	13	5	4	10	Y
GBG12_HUMAN	GNG12	Q9UBI6	Guanine nucleotide-binding protein G(I)/G(S)/G(O) subunit gamma-12	50	33	10	10	2	10	Y
DYN2_HUMAN	DNM2	P50570	Dynamin-2 (EC 3.6.5.5)	18.7	32	4	3	11	15	Y
ARF4_HUMAN	ARF4	P18085	ADP-ribosylation factor 4	33.9	32	6	6	5	15	Y
DCTN1_HUMAN	DCTN1	Q14203	Dynactin subunit 1 (150 kDa dynein-associated polypeptide) (DAP-150) (	7.67	32	10	3	8	11	Y
RASN_HUMAN	NRAS	P01111	GTPase NRas (Transforming protein N-Ras)	40.2	32	11	10	2	9	Y
SYWC_HUMAN	WARS	P23381	Tryptophan--tRNA ligase, cytoplasmic (EC 6.1.1.2) (Interferon-induced	19.3	32	21	1	1	9	Y
RAGP1_HUMAN	RANGAP1	P46060	Ran GTPase-activating protein 1 (RanGAP1)	16	32	17	4	6	6	Y
H12_HUMAN	HIST1H1C	P16403	Histone H1.2 (Histone H1c) (Histone H1d) (Histone H1s-1)	36.2	31	7	2	3	20	Y
IF2A_HUMAN	EIF2S1	P05198	Eukaryotic translation initiation factor 2 subunit 1 (Eukaryotic trans	22.9	31	14	1	5	13	Y
MMS19_HUMAN	MMS19	Q96T76	MMS19 nucleotide excision repair protein homolog (hMMS19) (MET18 homol	11.7	31	9	2	8	12	Y
PDCD6_HUMAN	PDCD6	O75340	Programmed cell death protein 6 (Apoptosis-linked gene 2 protein) (Pro	36.6	31	15	3	5	10	Y

RHG01_HUMAN	ARHGAP1	Q07960	Rho GTPase-activating protein 1 (CDC42 GTPase-activating protein) (GTP	17.1	31	9	7	4	10	Y
BIEA_HUMAN	BLVRA	P53004	Biliverdin reductase A (BVR A) (EC 1.3.1.24) (Biliverdin-IX alpha-redu	22.3	31	11	3	10	6	Y
MDHC_HUMAN	MDH1	P40925	Malate dehydrogenase, cytoplasmic (EC 1.1.1.37) (Cytosolic malate dehy	24.3	31	17	4	6	6	Y
Q6NUK7_HUMAN	LYN	Q6NUK7	LYN protein (Fragment)	19.9	31	16	3	5	6	Y
BZW1_HUMAN	BZW1	Q7L1Q6	Basic leucine zipper and W2 domain-containing protein 1 (Protein Orf)	17.7	31	11	4	13	5	Y
LAT1_HUMAN	SLC7A5	Q01650	Large neutral amino acids transporter small subunit 1 (4F2 light chain	11.2	31	14	4	9	4	Y
B3KQT9_HUMAN	B3KQT9_HUMAN	B3KQT9	cDNA PSEC0175 fis, clone OVARC1000169, highly similar to Protein disul	24.6	31	16	11	2	2	
H2AY_HUMAN	H2AFY	O75367	Core histone macro-H2A.1 (Histone macroH2A1) (mH2A1) (Histone H2A.y) (	29.8	30	6	2	3	19	Y
B2R806_HUMAN	EIF3E	B2R806	Eukaryotic translation initiation factor 3 subunit E (eIF3e) (Eukaryot	21.6	30	7	1	8	16	Y
A6NJA2_HUMAN	USP14	A6NJA2	Ubiquitin carboxyl-terminal hydrolase (EC 3.4.19.12)	8.93	30	7	3	10	10	Y
XPP1_HUMAN	XPNPEP1	Q9NQW7	Xaa-Pro aminopeptidase 1 (EC 3.4.11.9) (Aminoacylproline aminopeptidas	11.7	30	11	4	6	9	Y
F8W914_HUMAN	RTN4	F8W914	Reticulon	12.8	30	7	8	9	7	Y
E7ES33_HUMAN	Sep-07	E7ES33	Septin-7	9.17	30	8	8	7	7	Y
AT1B1_HUMAN	ATP1B1	P05026	Sodium/potassium-transporting ATPase subunit beta-1 (Sodium/potassium-	18.5	30	15	6	4	5	Y
B4DZF2_HUMAN	B4DZF2_HUMAN	B4DZF2	cDNA FLJ59571, highly similar to Eukaryotic translation initiation fac	10	29	5	1	7	19	
Q9H9B7_HUMAN	Q9H9B7_HUMAN	Q9H9B7	Coatomer subunit gamma	11	29	3	4	7	16	
THIL_HUMAN	ACAT1	P24752	Acetyl-CoA acetyltransferase, mitochondrial (EC 2.3.1.9) (Acetoacetyl-	18.7	29	6	2	7	14	Y
Q5JQ44_HUMAN	DKFZp547A0616	Q5JQ44	Putative uncharacterized protein DKFZp547A0616 (Fragment)	35.4	29	10	6	2	11	
E9PAV3_HUMAN	NACA	E9PAV3	Nascent polypeptide-associated complex subunit alpha	3.46	29	10	3	7	9	Y
AP1G1_HUMAN	AP1G1	O43747	AP-1 complex subunit gamma-1 (Adapter-related protein complex 1 subuni	9.25	29	7	7	7	8	Y
J3QQX2_HUMAN	ARHGDIA	J3QQX2	Rho GDP-dissociation inhibitor 1	19.1	29	12	6	4	7	Y
EF1D_HUMAN	EEF1D	P29692	Elongation factor 1-delta (EF-1-delta) (Antigen NY-CO-4)	22.8	29	12	3	8	6	Y
RAB5B_HUMAN	RAB5B	P61020	Ras-related protein Rab-5B	21.4	29	13	5	5	6	Y
ARPC2_HUMAN	ARPC2	O15144	Actin-related protein 2/3 complex subunit 2 (Arp2/3 complex 34 kDa sub	29	29	13	8	7	3	Y
OXSR1_HUMAN	OXSR1	O95747	Serine/threonine-protein kinase OSR1 (EC 2.7.11.1) (Oxidative stress-r	14.6	28	9	2	3	14	Y
PTPRJ_HUMAN	PTPRJ	Q12913	Receptor-type tyrosine-protein phosphatase eta (Protein-tyrosine phosp	8.3	28	7	4	5	12	Y

A8KA19_HUMAN	A8KA19_HUMAN	A8KA19	cDNA FLJ75831, highly similar to Homo sapiens exportin, tRNA (nuclear	9.15	28	6	4	8	10	
Q6IBU0_HUMAN	EIF5	Q6IBU0	EIF5 protein	10.7	28	8	5	8	9	Y
DRG1_HUMAN	DRG1	Q9Y295	Developmentally-regulated GTP-binding protein 1 (DRG-1) (Neural precur	18.8	28	10	2	7	9	Y
B4DTC3_HUMAN	HNRNPD	B4DTC3	Heterogeneous nuclear ribonucleoprotein D0 (cDNA FLJ54150, highly simi	16.8	28	8	6	8	5	Y
A8K0T9_HUMAN	A8K0T9_HUMAN	A8K0T9	cDNA FLJ75422, highly similar to Homo sapiens capping protein (actin f	24.5	28	11	5	7	5	
H0YGW7_HUMAN	ABCF1	H0YGW7	ATP-binding cassette sub-family F member 1 (Fragment)	17.1	27	5	4	2	16	Y
DC1L1_HUMAN	DYNC1LI1	Q9Y6G9	Cytoplasmic dynein 1 light intermediate chain 1 (LIC1) (Dynein light c	16.6	27	6	1	9	11	Y
Q6NZ55_HUMAN	RPL13	Q6NZ55	60S ribosomal protein L13	22.7	27	10	4	5	8	Y
VTNC_HUMAN	VTN	P04004	Vitronectin (VN) (S-protein) (Serum-spreading factor) (V75) [Cleaved i	15.1	27	3	3	15	6	Y
B4DRT4_HUMAN	B4DRT4_HUMAN	B4DRT4	cDNA FLJ51535, highly similar to Phosphatidylethanolamine-binding prot	31.6	27	10	8	4	5	
FLOT2_HUMAN	FLOT2	Q14254	Flotillin-2 (Epidermal surface antigen) (ESA) (Membrane component chro	34.6	27	5	12	8	1	Y
A8K690_HUMAN	A8K690_HUMAN	A8K690	cDNA FLJ76863, highly similar to Homo sapiens stress-induced-phosphopr	24.5	27	20	3	4	1	
Q05D43_HUMAN	YBX1	Q05D43	YBX1 protein (Fragment)	19.5	26	5	2	5	14	Y
PCH2_HUMAN	TRIP13	Q15645	Pachytene checkpoint protein 2 homolog (Human papillomavirus type 16 E	21.3	26	10	1	4	11	Y
IPYR_HUMAN	PPA1	Q15181	Inorganic pyrophosphatase (EC 3.6.1.1) (Pyrophosphate phospho-hydrolas	20.4	26	10	3	5	9	Y
THIO_HUMAN	TXN	P10599	Thioredoxin (Trx) (ATL-derived factor) (ADF) (Surface-associated sulph	42.9	26	11	4	4	9	Y
B2RE34_HUMAN	B2RE34_HUMAN	B2RE34	cDNA, FLJ96901, highly similar to Homo sapiens Rac GTPase activating p	11.7	26	11	12	1	4	
PRDX5_HUMAN	PRDX5	P30044	Peroxiredoxin-5, mitochondrial (EC 1.11.1.15) (Alu corepressor 1) (Ant	36.9	26	17	5	2	3	Y
B2R9S4_HUMAN	B2R9S4_HUMAN	B2R9S4	cDNA, FLJ94534, highly similar to Homo sapiens capping protein (actin	17.2	25	7	1	1	17	
LRC47_HUMAN	LRRC47	Q8N1G4	Leucine-rich repeat-containing protein 47	15.4	25	3	2	4	16	Y
H13_HUMAN	HIST1H1D	P16402	Histone H1.3 (Histone H1c) (Histone H1s-2)	21.3	25	6	2	3	15	Y
AAKG1_HUMAN	PRKAG1	P54619	5'-AMP-activated protein kinase subunit gamma-1 (AMPK gamma1) (AMPK su	15.1	25	10	1	3	11	Y
E5RI99_HUMAN	RPL30	E5RI99	60S ribosomal protein L30 (Fragment)	35.1	25	8	2	6	10	Y
RL21_HUMAN	RPL21	P46778	60S ribosomal protein L21	16.2	25	7	6	4	8	Y
M0R0F0_HUMAN	RPS5	M0R0F0	40S ribosomal protein S5 (Fragment)	27	25	7	4	7	7	Y
SCRIB_HUMAN	SCRIB	Q14160	Protein scribble homolog (Scribble) (hScrib) (Protein LAP4)	6.13	25	15	2	2	5	Y

FRIL_HUMAN	FTL	P02792	Ferritin light chain (Ferritin L subunit)	21.7	25	10	4	7	3	Y
B3KU28_HUMAN	LONP1	B3KU28	Lon protease homolog, mitochondrial (EC 3.4.21.-) (Lon protease-like p	11.7	24	1	1	4	18	Y
RL14_HUMAN	RPL14	P50914	60S ribosomal protein L14 (CAG-ISL 7)	30.7	24	7	3	2	13	Y
TNIK_HUMAN	TNIK	Q9UKE5	TRAF2 and NCK-interacting protein kinase (EC 2.7.11.1)	3.31	24	7	7	1	10	Y
B4DQ93_HUMAN	B4DQ93_HUMAN	B4DQ93	cDNA FLJ52996, highly similar to Syntenin-1	16.9	24	1	7	10	7	
DLG1_HUMAN	DLG1	Q12959	Disks large homolog 1 (Synapse-associated protein 97) (SAP-97) (SAP97)	5.97	24	10	6	1	7	Y
RS13_HUMAN	RPS13	P62277	40S ribosomal protein S13	28.5	24	13	2	1	7	Y
I3L397_HUMAN	EIF5A	I3L397	Eukaryotic translation initiation factor 5A-1 (Fragment)	16.3	24	7	7	7	6	Y
A6NFQ9_HUMAN	Sep-08	A6NFQ9	Septin-8	9.09	24	9	4	5	6	Y
A8K607_HUMAN	A8K607_HUMAN	A8K607	cDNA FLJ76855, highly similar to Homo sapiens exportin 7 (XPO7), mRNA	12.2	24	3	1	17	4	
NAGK_HUMAN	NAGK	Q9UJ70	N-acetyl-D-glucosamine kinase (N-acetylglucosamine kinase) (EC 2.7.1.5	18.6	23	3	1	4	15	Y
ARF6_HUMAN	ARF6	P62330	ADP-ribosylation factor 6	18.3	23	6	3	3	12	Y
DNJB1_HUMAN	DNAJB1	P25685	DnaJ homolog subfamily B member 1 (DnaJ protein homolog 1) (Heat shock	18.5	23	6	3	6	10	Y
A6NNI4_HUMAN	CD9	A6NNI4	CD9 antigen	20.8	23	6	6	5	6	Y
CAZA2_HUMAN	CAPZA2	P47755	F-actin-capping protein subunit alpha-2 (CapZ alpha-2)	19.2	23	5	5	9	4	Y
LASP1_HUMAN	LASP1	Q14847	LIM and SH3 domain protein 1 (LASP-1) (Metastatic lymph node gene 50 p	20.7	23	12	3	6	2	Y
SPEE_HUMAN	SRM	P19623	Spermidine synthase (SPDSY) (EC 2.5.1.16) (Putrescine aminopropyltrans	15.2	22	3	2	2	16	Y
DNM1L_HUMAN	DNM1L	O00429	Dynamin-1-like protein (EC 3.6.5.5) (Dnm1p/Vps1p-like protein) (DVLP)	10.3	22	4	2	4	13	Y
CISY_HUMAN	CS	O75390	Citrate synthase, mitochondrial (EC 2.3.3.1)	10.3	22	7	2	5	9	Y
H31T_HUMAN	HIST3H3	Q16695	Histone H3.1t (H3/t) (H3t) (H3/g)	19.9	22	4	3	6	8	Y
IGSF8_HUMAN	IGSF8	Q969P0	Immunoglobulin superfamily member 8 (IgSF8) (CD81 partner 3) (Glu-Trp-	8.16	22	7	2	5	8	Y
B4DN67_HUMAN	KIRREL	B4DN67	Kin of IRRE-like protein 1 (cDNA FLJ56360, highly similar to Kin of IR	8.52	22	10	1	4	7	Y
PURA2_HUMAN	ADSS	P30520	Adenylosuccinate synthetase isozyme 2 (AMPSase 2) (AdSS 2) (EC 6.3.4.4	8.33	22	9	3	5	5	Y
B4DSN3_HUMAN	B4DSN3_HUMAN	B4DSN3	cDNA FLJ60345, highly similar to Protein transport protein Sec24C	6.36	22	8	4	7	4	
Q6FHJ5_HUMAN	SCAMP3	Q6FHJ5	SCAMP3 protein (Fragment)	9.8	22	12	4	3	3	Y
Q9BQQ5_HUMAN	L27a	Q9BQQ5	Ribosomal protein L27a	51.9	21	7	2	2	11	

H3BLU7_HUMAN	AKR7A2	H3BLU7	Aflatoxin B1 aldehyde reductase member 2 (Fragment)	14.6	21	4	3	4	10	Y
B4E1U9_HUMAN	B4E1U9_HUMAN	B4E1U9	cDNA FLJ54776, highly similar to Cell division control protein 42 homo	17.8	21	8	4	3	6	
TRI25_HUMAN	TRIM25	Q14258	E3 ubiquitin/ISG15 ligase TRIM25 (EC 6.3.2.19) (EC 6.3.2.n3) (Estrogen	10.2	21	13	1	1	6	Y
Q59F71_HUMAN	Q59F71_HUMAN	Q59F71	Bone morphogenetic protein 1 isoform 1, variant (Fragment)	3.99	21	8	2	6	5	
F2Z393_HUMAN	TALDO1	F2Z393	Transaldolase (EC 2.2.1.2)	22.3	21	5	3	12	2	Y
MET_HUMAN	MET	P08581	Hepatocyte growth factor receptor (HGF receptor) (EC 2.7.10.1) (HGF/SF	7.48	21	10	4	5	2	Y
PSB2_HUMAN	PSMB2	P49721	Proteasome subunit beta type-2 (EC 3.4.25.1) (Macropain subunit C7-I)	29.9	21	6	4	10	1	Y
2ABA_HUMAN	PPP2R2A	P63151	Serine/threonine-protein phosphatase 2A 55 kDa regulatory subunit B al	11.9	20	6	3	3	9	Y
RUXE_HUMAN	SNRPE	P62304	Small nuclear ribonucleoprotein E (snRNP-E) (Sm protein E) (Sm-E) (SmE	29.3	20	6	2	4	8	Y
ARPC3_HUMAN	ARPC3	O15145	Actin-related protein 2/3 complex subunit 3 (Arp2/3 complex 21 kDa sub	19.1	20	5	6	4	7	Y
F8W6I7_HUMAN	HNRNPA1	F8W6I7	Heterogeneous nuclear ribonucleoprotein A1	18.1	20	11	2	2	7	Y
B0YIW6_HUMAN	ARCN1	B0YIW6	Archain 1, isoform CRA_a (Coatomer subunit delta) (Coatomer subunit de	13	20	10	1	4	5	Y
C9J6P4_HUMAN	ZC3HAV1	C9J6P4	Zinc finger CCCH-type antiviral protein 1	9.57	20	12	1	4	4	Y
ADA10_HUMAN	ADAM10	O14672	Disintegrin and metalloproteinase domain-containing protein 10 (ADAM 1	9.49	20	2	4	13	2	Y
A8K4W5_HUMAN	A8K4W5_HUMAN	A8K4W5	cDNA FLJ76813, highly similar to Homo sapiens acetyl-Coenzyme A acetyl	12.6	20	14	1	4	1	
ITCH_HUMAN	ITCH	Q96J02	E3 ubiquitin-protein ligase Itchy homolog (Itch) (EC 6.3.2.-) (Atrophi	11.6	19	10	1	1	9	Y
B2RD51_HUMAN	B2RD51_HUMAN	B2RD51	cDNA, FLJ96455, highly similar to Homo sapiens Down syndrome critical	13.9	19	5	2	5	7	
K6PF_HUMAN	PFKM	P08237	6-phosphofructokinase, muscle type (EC 2.7.1.11) (Phosphofructo-1-kina	8.97	19	8	2	2	7	Y
RL11_HUMAN	RPL11	P62913	60S ribosomal protein L11 (CLL-associated antigen KW-12)	23.6	19	6	6	3	5	Y
ARRD1_HUMAN	ARRDC1	Q8N5I2	Arrestin domain-containing protein 1	10.4	19	2	5	8	4	Y
METK2_HUMAN	MAT2A	P31153	S-adenosylmethionine synthase isoform type-2 (AdoMet synthase 2) (EC 2	13.9	19	5	1	10	3	Y
EXOC4_HUMAN	EXOC4	Q96A65	Exocyst complex component 4 (Exocyst complex component Sec8)	7.7	19	7	6	4	3	Y
AP2M1_HUMAN	AP2M1	Q96CW1	AP-2 complex subunit mu (AP-2 mu chain) (Adapter-related protein compl	15.6	18	3	3	2	11	Y
PPAC_HUMAN	ACP1	P24666	Low molecular weight phosphotyrosine protein phosphatase (LMW-PTP) (LM	32.9	18	3	2	6	10	Y
F8W1F5_HUMAN	FMNL3	F8W1F5	Formin-like protein 3	7.21	18	5	4	1	10	Y
PRP19_HUMAN	PRPF19	Q9UMS4	Pre-mRNA-processing factor 19 (Nuclear matrix protein 200) (PRP19/PSO4	9.92	18	3	2	5	9	Y

CN166_HUMAN	C14orf166	Q9Y224	UPF0568 protein C14orf166 (CLE7 homolog) (CLE)	22.5	18	4	1	5	8	Y
B5ME97_HUMAN	Sep-10	B5ME97	Septin 10, isoform CRA_c (Septin-10)	9.01	18	3	4	3	8	Y
RS25_HUMAN	RPS25	P62851	40S ribosomal protein S25	24	18	8	2	3	7	Y
UAP1_HUMAN	UAP1	Q16222	UDP-N-acetylhexosamine pyrophosphorylase (Antigen X) (AGX) (Sperm-asso	10.7	18	4	6	1	7	Y
A8K4G7_HUMAN	A8K4G7_HUMAN	A8K4G7	cDNA FLJ78528, highly similar to Homo sapiens vacuolar protein sorting	16.7	18	5	2	5	6	
MIF_HUMAN	MIF	P14174	Macrophage migration inhibitory factor (MIF) (EC 5.3.2.1) (Glycosylati	17.4	18	6	3	3	6	Y
B4E0U6_HUMAN	B4E0U6_HUMAN	B4E0U6	cDNA FLJ53687, highly similar to Hsc70-interacting protein	12.3	18	7	4	1	6	
E9PIF1_HUMAN	CD81	E9PIF1	CD81 antigen	9.92	18	7	4	5	5	Y
R4GN98_HUMAN	S100A6	R4GN98	Protein S100-A6 (Fragment)	37.6	18	5	3	5	5	Y
B7Z992_HUMAN	B7Z992_HUMAN	B7Z992	cDNA FLJ53698, highly similar to Gelsolin	6.43	18	2	8	3	5	
B5BUB5_HUMAN	SSB	B5BUB5	Autoantigen La (Fragment)	6.86	18	5	4	6	3	Y
A8K5U9_HUMAN	A8K5U9_HUMAN	A8K5U9	cDNA FLJ75056, highly similar to Homo sapiens phosphatidylinositol bin	8.44	18	6	7	2	3	
DEK_HUMAN	DEK	P35659	Protein DEK	16.3	17	1	2	3	11	Y
J3KMZ9_HUMAN	LDLR	J3KMZ9	Low-density lipoprotein receptor (Fragment)	4.23	17	1	3	4	10	Y
B4DVS0_HUMAN	SNRPB	B4DVS0	Small nuclear ribonucleoprotein-associated protein	12.8	17	3	2	5	8	Y
ECHA_HUMAN	HADHA	P40939	Trifunctional enzyme subunit alpha, mitochondrial (78 kDa gastrin-bind	6.95	17	5	1	4	8	Y
Q53HL1_HUMAN	Q53HL1_HUMAN	Q53HL1	Myosin regulatory light chain MRCL3 variant (Fragment)	18.7	17	4	3	3	8	
RS20_HUMAN	RPS20	P60866	40S ribosomal protein S20	19.3	17	4	2	6	7	Y
RS26_HUMAN	RPS26	P62854	40S ribosomal protein S26	40	17	5	3	2	7	Y
HNRDL_HUMAN	HNRNPDL	O14979	Heterogeneous nuclear ribonucleoprotein D-like (hnRNP D-like) (hnRNP D	10	17	7	1	3	6	
NICA_HUMAN	NCSTN	Q92542	Nicastrin	7.05	17	7	4	1	6	Y
SMD1_HUMAN	SNRPD1	P62314	Small nuclear ribonucleoprotein Sm D1 (Sm-D1) (Sm-D autoantigen) (snRN	20.2	17	7	2	4	5	Y
B4DLC0_HUMAN	PCBP2	B4DLC0	Poly(rC)-binding protein 2 (cDNA FLJ58476, highly similar to Poly(rC)-	14.3	17	5	3	5	4	Y
B7Z7F0_HUMAN	B7Z7F0_HUMAN	B7Z7F0	cDNA FLJ56420, highly similar to Aspartyl aminopeptidase (EC 3.4.11.21	7.71	17	5	2	7	3	
B2RAH7_HUMAN	B2RAH7_HUMAN	B2RAH7	cDNA, FLJ94921, highly similar to Homo sapiens prolyl endopeptidase (P	6.48	17	7	1	6	3	
SUCB2_HUMAN	SUCLG2	Q96I99	Succinyl-CoA ligase [GDP-forming] subunit beta, mitochondrial (EC 6.2.	12.5	17	10	1	3	3	Y

CYBP_HUMAN	CACYBP	Q9HB71	Calcyclin-binding protein (CacyBP) (hCacyBP) (S100A6-binding protein)	18.9	17	9	2	4	1	Y
B4DF70_HUMAN	B4DF70_HUMAN	B4DF70	cDNA FLJ60461, highly similar to Peroxiredoxin-2 (EC 1.11.1.15)	27.3	16	2	8	1	6	
F8VXU5_HUMAN	VPS29	F8VXU5	Vacuolar protein sorting-associated protein 29	10.7	16	5	2	3	5	Y
B5LY71_HUMAN	UMPS	B5LY71	Uridine monophosphate synthetase isoform I	10	16	5	2	5	4	Y
A8K8A4_HUMAN	A8K8A4_HUMAN	A8K8A4	cDNA FLJ77640, highly similar to Homo sapiens copine II (CPNE2), mRNA	7.3	16	6	4	2	4	
B4DM33_HUMAN	B4DM33_HUMAN	B4DM33	cDNA FLJ52068, highly similar to Microtubule-associated protein RP/EB	13.9	16	8	4	4	3	
Q6NZ61_HUMAN	RHEB	Q6NZ61	Ras homolog enriched in brain	33.2	16	9	2	2	3	Y
VATE1_HUMAN	ATP6V1E1	P36543	V-type proton ATPase subunit E 1 (V-ATPase subunit E 1) (V-ATPase 31 k	11.1	16	6	6	1	3	Y
PCBP1_HUMAN	PCBP1	Q15365	Poly(rC)-binding protein 1 (Alpha-CP1) (Heterogeneous nuclear ribonucl	15.2	16	4	3	7	2	Y
GNPI1_HUMAN	GNPDA1	P46926	Glucosamine-6-phosphate isomerase 1 (EC 3.5.99.6) (Glucosamine-6-phosp	18.7	16	6	7	2	1	Y
B7ZKT9_HUMAN	KIAA1033	B7ZKT9	KIAA1033 protein	4.51	16	10	4	1	1	Y
F5GX77_HUMAN	TRMT112	F5GX77	tRNA methyltransferase 112 homolog	26.4	15	4	1	2	8	Y
AP1M1_HUMAN	AP1M1	Q9BXS5	AP-1 complex subunit mu-1 (AP-mu chain family member mu1A) (Adapter-re	5.67	15	4	1	5	6	Y
J3K000_HUMAN	PEPD	J3K000	PEPD protein	5.27	15	4	3	4	4	Y
CPNS1_HUMAN	CAPNS1	P04632	Calpain small subunit 1 (CSS1) (Calcium-activated neutral proteinase s	10.8	15	7	3	2	4	Y
PUR8_HUMAN	ADSL	P30566	Adenylosuccinate lyase (ASL) (EC 4.3.2.2) (Adenylosuccinase) (ASase)	15.7	15	9	1	2	4	Y
D3DNI3_HUMAN	PFN2	D3DNI3	Profilin (Fragment)	10.6	15	4	2	6	3	Y
MCA3_HUMAN	EEF1E1	O43324	Eukaryotic translation elongation factor 1 epsilon-1 (Aminoacyl tRNA s	12.1	15	4	3	5	3	Y
ACPH_HUMAN	APEH	P13798	Acylamino-acid-releasing enzyme (AARE) (EC 3.4.19.1) (Acyl-peptide hyd	7.79	15	2	3	9	2	Y
VP26A_HUMAN	VPS26A	O75436	Vacuolar protein sorting-associated protein 26A (Vesicle protein sorti	18.7	15	7	3	2	2	Y
Q59GU6_HUMAN	Q59GU6_HUMAN	Q59GU6	Sorting nexin 1 isoform a variant (Fragment)	12.7	15	1	6	6	1	
A8K556_HUMAN	A8K556_HUMAN	A8K556	cDNA FLJ78217	5.6	15	2	8	4	1	
EXT2_HUMAN	EXT2	Q93063	Exostosin-2 (EC 2.4.1.224) (EC 2.4.1.225) (Glucuronosyl-N-acetylglucos	10.3	15	10	3	1	1	Y
VAC14_HUMAN	VAC14	Q08AM6	Protein VAC14 homolog (Tax1-binding protein 2)	8.7	14	2	3	1	9	Y
RL35A_HUMAN	RPL35A	P18077	60S ribosomal protein L35a (Cell growth-inhibiting gene 33 protein)	26.4	14	2	2	4	6	Y
DHSO_HUMAN	SORD	Q00796	Sorbitol dehydrogenase (EC 1.1.1.14) (L-iditol 2-dehydrogenase)	9.24	14	3	2	3	6	Y

A8K7A4_HUMAN	A8K7A4_HUMAN	A8K7A4	cDNA FLJ76904, highly similar to Homo sapiens methionine adenosyltrans	7.74	14	1	2	6	5	
B2R5I8_HUMAN	B2R5I8_HUMAN	B2R5I8	cDNA, FLJ92490, highly similar to Homo sapiens RAB32, member RAS oncog	9.78	14	2	3	4	5	
B4DEK8_HUMAN	B4DEK8_HUMAN	B4DEK8	cDNA FLJ53721, highly similar to Tetraspanin-3	7.44	14	3	3	3	5	
UBP7_HUMAN	USP7	Q93009	Ubiquitin carboxyl-terminal hydrolase 7 (EC 3.4.19.12) (Deubiquitinati	4.54	14	5	1	4	4	Y
BZW2_HUMAN	BZW2	Q9Y6E2	Basic leucine zipper and W2 domain-containing protein 2	9.79	14	8	1	4	1	Y
VASP_HUMAN	VASP	P50552	Vasodilator-stimulated phosphoprotein (VASP)	10	13	2	2	3	8	Y
DNJA1_HUMAN	DNAJA1	P31689	DnaJ homolog subfamily A member 1 (DnaJ protein homolog 2) (HSDJ) (Hea	11.8	13	2	1	6	4	Y
ARC1B_HUMAN	ARPC1B	O15143	Actin-related protein 2/3 complex subunit 1B (Arp2/3 complex 41 kDa su	12.4	13	5	3	3	3	Y
EF1B_HUMAN	EEF1B2	P24534	Elongation factor 1-beta (EF-1-beta)	12.4	13	4	4	2	3	Y
D6RER5_HUMAN	Sep-11	D6RER5	Septin-11	5.09	13	4	4	2	3	Y
B4E2J3_HUMAN	PRSS23	B4E2J3	Serine protease 23 (cDNA FLJ51190, highly similar to Serine protease 2	9.4	13	4	2	4	2	Y
NPM_HUMAN	NPM1	P06748	Nucleophosmin (NPM) (Nucleolar phosphoprotein B23) (Nucleolar protein	14.6	12	1	1	5	7	Y
F2Z2E2_HUMAN	IQGAP3	F2Z2E2	Ras GTPase-activating-like protein IQGAP3	3.78	12	3	1	2	6	Y
CKAP5_HUMAN	CKAP5	Q14008	Cytoskeleton-associated protein 5 (Colonic and hepatic tumor overexpre	3.54	12	3	5	1	4	Y
CD151_HUMAN	CD151	P48509	CD151 antigen (GP27) (Membrane glycoprotein SFA-1) (Platelet-endotheli	7.11	12	3	5	1	3	Y
AL3B1_HUMAN	ALDH3B1	P43353	Aldehyde dehydrogenase family 3 member B1 (EC 1.2.1.5) (Aldehyde dehyd	11.1	12	2	2	6	2	Y
D6RAX7_HUMAN	COPS4	D6RAX7	COP9 signalosome complex subunit 4	11.2	12	3	2	6	1	Y
PA1B3_HUMAN	PAFAH1B3	Q15102	Platelet-activating factor acetylhydrolase IB subunit gamma (EC 3.1.1.	11.3	11	3	2	2	4	Y
IMA5_HUMAN	KPNA1	P52294	Importin subunit alpha-5 (Karyopherin subunit alpha-1) (Nucleoprotein	5.95	11	4	2	1	4	Y
ERF1_HUMAN	ETF1	P62495	Eukaryotic peptide chain release factor subunit 1 (Eukaryotic release	5.26	11	4	1	3	3	Y
B4E1G6_HUMAN	GALK1	B4E1G6	Galactokinase (Galactokinase 1) (cDNA FLJ56840, highly similar to Gala	9.95	11	2	1	6	2	Y
E9PFN5_HUMAN	GSTK1	E9PFN5	Glutathione S-transferase kappa 1	15.8	11	5	1	4	2	Y
B4DLB8_HUMAN	B4DLB8_HUMAN	B4DLB8	cDNA FLJ52205, highly similar to Beta-1,4-galactosyltransferase 1 (EC	9.3	11	6	1	3	2	
LEG3_HUMAN	LGALS3	P17931	Galectin-3 (Gal-3) (35 kDa lectin) (Carbohydrate-binding protein 35) (	16	11	2	5	2	2	Y
SMD3_HUMAN	SNRPD3	P62318	Small nuclear ribonucleoprotein Sm D3 (Sm-D3) (snRNP core protein D3)	15.1	10	3	1	1	5	Y
B2RDT8_HUMAN	B2RDT8_HUMAN	B2RDT8	cDNA, FLJ96764, highly similar to Homo sapiens sorting nexin 8 (SNX8),	11.8	10	4	2	1	3	

B2R679_HUMAN	B2R679_HUMAN	B2R679	cDNA, FLJ92825, highly similar to Homo sapiens SAR1a gene homolog 1 (S	11.6	10	3	3	2	2	
J3QK90_HUMAN	NSFL1C	J3QK90	NSFL1 cofactor p47	10.2	10	5	2	1	2	Y
CHIP_HUMAN	STUB1	Q9UNE7	E3 ubiquitin-protein ligase CHIP (EC 6.3.2.-) (Antigen NY-CO-7) (CLL-a	7.59	9	3	1	1	4	Y
ETUD1_HUMAN	EFTUD1	Q7Z2Z2	Elongation factor Tu GTP-binding domain-containing protein 1 (Elongati	1.43	9	2	1	3	3	Y
ASNA_HUMAN	ASNA1	O43681	ATPase ASNA1 (EC 3.6.-.-) (Arsenical pump-driving ATPase) (Arsenite-st	2.87	9	2	3	2	2	Y
B4DG42_HUMAN	B4DG42_HUMAN	B4DG42	cDNA FLJ53753, highly similar to Myeloid-associated differentiation ma	9.82	9	2	4	1	2	
DEST_HUMAN	DSTN	P60981	Destrin (Actin-depolymerizing factor) (ADF)	16.4	9	4	2	1	2	Y
6PGL_HUMAN	PGLS	O95336	6-phosphogluconolactonase (6PGL) (EC 3.1.1.31)	17.1	9	4	2	1	2	Y
NEDD4_HUMAN	NEDD4	P46934	E3 ubiquitin-protein ligase NEDD4 (EC 6.3.2.-) (Cell proliferation-ind	2.73	9	2	1	5	1	Y
B7Z2B0_HUMAN	B7Z2B0_HUMAN	B7Z2B0	cDNA FLJ53470, highly similar to Calcium/calmodulin-dependent protein	6.75	9	3	3	2	1	
M0QXB4_HUMAN	COPE	M0QXB4	Coatomer protein complex, subunit epsilon, isoform CRA_g (Coatomer sub	7.55	9	6	1	1	1	Y
C8C504_HUMAN	HBB	C8C504	Beta-globin	26.5	8	1	1	5	1	Y
PSB8_HUMAN	PSMB8	P28062	Proteasome subunit beta type-8 (EC 3.4.25.1) (Low molecular mass prote	13.4	8	2	2	3	1	Y
LRC59_HUMAN	LRRC59	Q96AG4	Leucine-rich repeat-containing protein 59 (Ribosome-binding protein p3	8.14	8	3	1	3	1	Y
CD47_HUMAN	CD47	Q08722	Leukocyte surface antigen CD47 (Antigenic surface determinant protein	5.26	8	4	2	1	1	Y
B2R6H7_HUMAN	B2R6H7_HUMAN	B2R6H7	cDNA, FLJ92955, highly similar to Homo sapiens transportin-SR (TRN-SR)	3.47	7	2	1	1	3	
HEXB_HUMAN	HEXB	P07686	Beta-hexosaminidase subunit beta (EC 3.2.1.52) (Beta-N-acetylhexosamin	4.5	7	2	2	2	1	Y
B4E1E2_HUMAN	B4E1E2_HUMAN	B4E1E2	cDNA FLJ61530, highly similar to Hepatocyte growth factor-regulated ty	3.33	7	2	2	2	1	
B3KM36_HUMAN	B3KM36_HUMAN	B3KM36	cDNA FLJ10153 fis, clone HEMBA1003417, highly similar to BAG family mo	15.6	7	2	3	1	1	
Q59FB9_HUMAN	Q59FB9_HUMAN	Q59FB9	Toll interacting protein variant (Fragment)	7.67	7	4	2	1	1	
B7Z561_HUMAN	B7Z561_HUMAN	B7Z561	cDNA FLJ53154, highly similar to Neutral amino acid transporter A	5.31	6	1	3	2	3	
A8K923_HUMAN	A8K923_HUMAN	A8K923	cDNA FLJ78690, highly similar to Homo sapiens protein phosphatase 4, r	2.36	6	1	1	2	2	
A4D0R2_HUMAN	SYPL	A4D0R2	Synaptophysin-like 1, isoform CRA_b (Synaptophysin-like protein)	4.56	6	2	3	2	1	
ARL3_HUMAN	ARL3	P36405	ADP-ribosylation factor-like protein 3	6.04	5	2	1	2	1	Y
C9JG87_HUMAN	MRPL39	C9JG87	39S ribosomal protein L39, mitochondrial (Fragment)	8.75	4	1	1	1	2	Y

Table S2. Relative quantification of selected exosome proteins involved in biogenesis, sorting/trafficking/release, and recognition/uptake by label-free spectral counting.

Biogenesis	Gene Name	Protein Accession	Protein Description	SpC Combined (4 cell lines)	Vesiclepedia
**Exosome Biogenesis**					
ESCRT-associated	CHMP4B	Q9H444	Charged multivesicular body protein 4b (Chromatin-modifying protein 4b	4	Y
	CHMP6	Q96FZ7	Charged multivesicular body protein 6 (Chromatin-modifying protein 6)	6	Y
	FAM129A	Q9BZQ8	Protein Niban (Cell growth-inhibiting gene 39 protein) (Protein FAM129	29	Y
	FAM129B	Q96TA1	Niban-like protein 1 (Meg-3) (Melanoma invasion by ERK) (MINERVA) (Pro	94	Y
	FAM49B	Q9NUQ9	Protein FAM49B (L1)	52	Y
	FAM98B	Q52LJ0	Protein FAM98B	2	
	PDCD6IP	Q8WUM4	Programmed cell death 6-interacting protein (PDCD6-interacting protein	273	Y
	TSG101	Q99816	Tumor susceptibility gene 101 protein (ESCRT-I complex subunit TSG101)	50	Y
	VPS16	Q9H269	Vacuolar protein sorting-associated protein 16 homolog (hVPS16)	3	
	VPS26A	O75436	Vacuolar protein sorting-associated protein 26A (Vesicle protein sorti	15	Y
	VPS29	F8VXU5	Vacuolar protein sorting-associated protein 29	16	Y
	VPS35	Q96QK1	Vacuolar protein sorting-associated protein 35 (hVPS35) (Maternal-embr	121	Y
	VPS37B	F5H4M0	Vacuolar protein sorting-associated protein 37B (Fragment)	7	Y
	VPS45	Q9NRW7	Vacuolar protein sorting-associated protein 45 (h-VPS45) (hlVps45)	4	Y
	VPS4A	Q9UN37	Vacuolar protein sorting-associated protein 4A (EC 3.6.4.6) (Protein S	16	Y
	VPS51	Q9UID3	Vacuolar protein sorting-associated protein 51 homolog (Another new ge	4	
	VTA1	Q9NP79	Vacuolar protein sorting-associated protein VTA1 homolog (Dopamine-res	3	Y
Tetraspanin	CD151	P48509	CD151 antigen (GP27) (Membrane glycoprotein SFA-1) (Platelet-endotheli	12	Y
	CD63	C9JV86	CD63 antigen	46	Y
	CD81	E9PIF1	CD81 antigen	18	Y
	CD82	P27701	CD82 antigen (C33 antigen) (IA4) (Inducible membrane protein R2) (Meta	50	Y
	CD9	A6NNI4	CD9 antigen	23	Y
	CLDN2	P57739	Claudin-2 (SP82)	12	
	TMEM132A	Q24JP5	Transmembrane protein 132A (HSPA5-binding protein 1)	9	
	TMEM189	G3V2F7	HCG2044781 (Protein TMEM189-UBE2V1) (Transmembrane protein 189)	7	
	TMEM2	Q9UHN6	Transmembrane protein 2	6	Y
	TMEM43	Q9BTV4	Transmembrane protein 43 (Protein LUMA)	2	
	TSPAN15	H7C285	Tetraspanin-15 (Fragment)	3	Y
	TSPAN4	J3KQ42	Tetraspanin-4	5	Y

	TSPAN9	B5MD23	Tetraspanin-9	6	Y
**Sorting/Trafficking & Release**					
GTPase	RAB10	P61026	Ras-related protein Rab-10	63	Y
	RAB11B	Q15907	Ras-related protein Rab-11B (GTP-binding protein YPT3)	50	Y
	RAB11FIP1	Q6WKZ4	Rab11 family-interacting protein 1 (Rab11-FIP1) (Rab-coupling protein)	3	
	RAB11FIP5	Q9BXF6	Rab11 family-interacting protein 5 (Rab11-FIP5) (Gamma-SNAP-associated	7	
	RAB13	Q504R6	RAB13 protein (Fragment)	31	Y
	RAB14	P61106	Ras-related protein Rab-14	57	Y
	RAB18	Q9NP72	Ras-related protein Rab-18	6	Y
	RAB1A	P62820	Ras-related protein Rab-1A (YPT1-related protein)	75	Y
	RAB1B	Q6FIG4	RAB1B protein	64	Y
	RAB21	Q9UL25	Ras-related protein Rab-21	20	Y
	RAB23	Q9ULC3	Ras-related protein Rab-23	16	
	RAB27A	H3BN55	Ras-related protein Rab-27A (Fragment)	10	Y
	RAB27B	O00194	Ras-related protein Rab-27B (C25KG)	10	Y
	RAB2A	P61019	Ras-related protein Rab-2A	73	Y
	RAB31	Q13636	Ras-related protein Rab-31 (Ras-related protein Rab-22B)	4	Y
	RAB34	A8MYQ9	Ras-related protein Rab-34, isoform NARR	16	Y
	RAB35	F5H157	Ras-related protein Rab-35 (Fragment)	45	Y
	RAB3B	P20337	Ras-related protein Rab-3B	17	Y
	RAB5A	B4DJA5	Ras-related protein Rab-5A (cDNA FLJ51867, highly similar to Ras-relat	36	Y
	RAB5B	P61020	Ras-related protein Rab-5B	29	Y
	RAB5C	P51148	Ras-related protein Rab-5C (L1880) (RAB5L)	55	Y
	RAB6A	Q6FGX3	RAB6A protein	22	Y
	RAB7A	P51149	Ras-related protein Rab-7a	80	Y
	RAB8A	P61006	Ras-related protein Rab-8A (Oncogene c-mel)	75	Y
	RAB8B	H0YNE9	Ras-related protein Rab-8B (Fragment)	67	Y
Sorting	ARCN1	B0YIW6	Archain 1, isoform CRA_a (Coatomer subunit delta) (Coatomer subunit de	20	Y
	ARF1	P84077	ADP-ribosylation factor 1	47	Y
	ARF4	P18085	ADP-ribosylation factor 4	32	Y
	ARF6	P62330	ADP-ribosylation factor 6	23	Y
	ARFIP1	P53367	Arfaptin-1 (ADP-ribosylation factor-interacting protein 1)	3	Y
	B2RAU5_HUMAN	B2RAU5	Sorting nexin	5	
	B4DDM5_HUMAN	B4DDM5	cDNA FLJ53298, highly similar to Peroxisomal multifunctional enzyme ty	13	
	B4DJW8_HUMAN	B4DJW8	cDNA FLJ55697, highly similar to Protein transport protein Sec23B	10	
	B4DSN3_HUMAN	B4DSN3	cDNA FLJ60345, highly similar to Protein transport protein Sec24C	22	
	CAV1	Q03135	Caveolin-1	13	Y

	CLTC	Q00610	Clathrin heavy chain 1 (Clathrin heavy chain on chromosome 17) (CLH-17	473	Y
	COPA	P53621	Coatomer subunit alpha (Alpha-coat protein) (Alpha-COP) (HEP-COP) (HEP	143	Y
	COPB1	P53618	Coatomer subunit beta (Beta-coat protein) (Beta-COP)	112	Y
	COPB2	B4DZI8	Coatomer protein complex, subunit beta 2 (Beta prime), isoform CRA_b (	37	Y
	COPE	M0QXB4	Coatomer protein complex, subunit epsilon, isoform CRA_g (Coatomer sub	9	Y
	COPG1	Q9Y678	Coatomer subunit gamma-1 (Gamma-1-coat protein) (Gamma-1-COP)	69	
	COPS4	D6RAX7	COP9 signalosome complex subunit 4	12	Y
	COPS7A	Q9UBW8	COP9 signalosome complex subunit 7a (SGN7a) (Signalosome subunit 7a) (	4	
	EEA1	Q15075	Early endosome antigen 1 (Endosome-associated protein p162) (Zinc fing	6	Y
	FLOT1	O75955	Flotillin-1	39	Y
	FLOT2	Q14254	Flotillin-2 (Epidermal surface antigen) (ESA) (Membrane component chro	27	Y
	IGFBP2	P18065	Insulin-like growth factor-binding protein 2 (IBP-2) (IGF-binding prot	6	
	LAP3	P28838	Cytosol aminopeptidase (EC 3.4.11.1) (Leucine aminopeptidase 3) (LAP-3	68	Y
	PARVA	J3KNQ4	Alpha-parvin (Parvin, alpha, isoform CRA_c)	10	
	PSMD8	K7EJC1	26S proteasome non-ATPase regulatory subunit 8	9	Y
	Q53GW1_HUMAN	Q53GW1	Vesicle transport-related protein isoform a variant (Fragment)	19	
	Q59GU6_HUMAN	Q59GU6	Sorting nexin 1 isoform a variant (Fragment)	15	
	RAC1	A4D2P0	Ras-related C3 botulinum toxin substrate 1 (Rho family, small GTP bind	57	Y
	RAC2	P15153	Ras-related C3 botulinum toxin substrate 2 (GX) (Small G protein) (p21	16	Y
	SEC23A	F5H365	Protein transport protein Sec23A	15	Y
	SNRNP200	O75643	U5 small nuclear ribonucleoprotein 200 kDa helicase (EC 3.6.4.13) (Act	81	
	SNX12	Q3SYF1	Sorting nexin 12 (Sorting nexin 12, isoform CRA_a) (cDNA, FLJ93438, Ho	2	Y
	SNX18	Q96RF0	Sorting nexin-18 (SH3 and PX domain-containing protein 3B) (Sorting ne	2	Y
	SNX5	Q9Y5X3	Sorting nexin-5	12	
	STX3	F8W9Y0	Syntaxin-3	10	Y
	TF	Q06AH7	Transferrin	202	Y
	YKT6	B5BU81	YKT6 v-SNARE protein	10	
Trafficking	DYNC1H1	Q14204	Cytoplasmic dynein 1 heavy chain 1 (Cytoplasmic dynein heavy chain 1)	750	Y
	DYNC1LI1	Q9Y6G9	Cytoplasmic dynein 1 light intermediate chain 1 (LIC1) (Dynein light c	27	Y
	DYNC1LI2	B4DZP4	Cytoplasmic dynein 1 light intermediate chain 2 (cDNA FLJ52924, highly	18	
	DYNLL2	Q96FJ2	Dynein light chain 2, cytoplasmic (8 kDa dynein light chain b) (DLC8b)	5	
	DYNLRB1	B4DFR2	Dynein light chain roadblock-type 1 (cDNA FLJ59194, moderately similar	3	
	HIST1H1B	P16401	Histone H1.5 (Histone H1a) (Histone H1b) (Histone H1s-3)	22	Y
	ITPA	Q9BY32	Inosine triphosphate pyrophosphatase (ITPase) (Inosine triphosphatase)	14	Y
	KIF11	P52732	Kinesin-like protein KIF11 (Kinesin-like protein 1) (Kinesin-like spin	2	
	KIF13B	Q9NQT8	Kinesin-like protein KIF13B (Kinesin-like protein GAKIN)	4	

	KIF23	Q02241	Kinesin-like protein KIF23 (Kinesin-like protein 5) (Mitotic kinesin-l	37	
	KIF5B	D3DRX6	Kinesin family member 5B, isoform CRA_a	57	Y
	KLC1	G3V5R9	Kinesin light chain 1	11	
	LDHA	P00338	L-lactate dehydrogenase A chain (LDH-A) (EC 1.1.1.27) (Cell proliferat	134	Y
	RAC1	A4D2P0	Ras-related C3 botulinum toxin substrate 1 (Rho family, small GTP bind	57	Y
	SKI2W	O76046	Putative RNA helicase Ski2w	12	
Release	DNM1	Q05193	Dynamin-1 (EC 3.6.5.5)	27	Y
	DNM2	P50570	Dynamin-2 (EC 3.6.5.5)	32	Y
	ERP29	P30040	Endoplasmic reticulum resident protein 29 (ERp29) (Endoplasmic reticul	11	
	ESYT1	Q9BSJ8	Extended synaptotagmin-1 (E-Syt1) (Membrane-bound C2 domain-containing	22	
	ESYT2	H7BXI1	Extended synaptotagmin-2 (Fragment)	4	
	IMPA1	P29218	Inositol monophosphatase 1 (IMP 1) (IMPase 1) (EC 3.1.3.25) (Inositol-	11	
	LAMP2	B4E2S7	Lysosome-associated membrane glycoprotein 2 (cDNA FLJ58780, highly sim	10	Y
	RNPS1	H3BV80	RNA-binding protein with serine-rich domain 1	6	
	SEC22B	O75396	Vesicle-trafficking protein SEC22b (ER-Golgi SNARE of 24 kDa) (ERS-24)	23	Y
	SNAP23	O00161	Synaptosomal-associated protein 23 (SNAP-23) (Vesicle-membrane fusion	23	Y
	STX3	F8W9Y0	Syntaxin-3	10	Y
	STX4	Q12846	Syntaxin-4 (Renal carcinoma antigen NY-REN-31)	28	Y
	STX7	O15400	Syntaxin-7	3	Y
	SYNGR2	Q3KQZ2	Synaptogyrin	2	Y
	SYPL	A4D0R2	Synaptophysin-like 1, isoform CRA_b (Synaptophysin-like protein)	6	Y
	UCHL1	P09936	Ubiquitin carboxyl-terminal hydrolase isozyme L1 (UCH-L1) (EC 3.4.19.1	36	
	VAMP3	Q9BRV4	Vesicle-associated membrane protein 3 (Cellubrevin)	16	Y
	VAT1	Q99536	Synaptic vesicle membrane protein VAT-1 homolog (EC 1.-.-.-)	85	Y
**Recognition & Uptake**					
Antigen-Presentation	HLA-A	E9ABI2	MHC class I antigen (Fragment)	104	Y
	HLA-B	Q0ZAX5	MHC class I antigen (Fragment)	87	Y
Other	CD44	E7EPC6	CD44 antigen	97	Y
	CD55	B1AP13	Complement decay-accelerating factor	2	Y
	CD58	B1AMW1	CD58 antigen, (Lymphocyte function-associated antigen 3), isoform CRA_	2	Y
	CD59	P13987	CD59 glycoprotein (1F5 antigen) (20 kDa homologous restriction factor)	3	Y
	PTGFRN	Q9P2B2	Prostaglandin F2 receptor negative regulator (CD9 partner 1) (CD9P-1)	54	Y
Adaptor protein complex	AP1B1	Q10567	AP-1 complex subunit beta-1 (Adapter-related protein complex 1 subunit	65	Y
	AP1G1	O43747	AP-1 complex subunit gamma-1 (Adapter-related protein complex 1 subuni	29	Y
	AP1M1	Q9BXS5	AP-1 complex subunit mu-1 (AP-mu chain family member mu1A) (Adapter-re	15	Y

	AP1S1	P61966	AP-1 complex subunit sigma-1A (Adapter-related protein complex 1 subun	2	Y
	AP2A1	O95782	AP-2 complex subunit alpha-1 (100 kDa coated vesicle protein A) (Adapt	94	Y
	AP2A2	O94973	AP-2 complex subunit alpha-2 (100 kDa coated vesicle protein C) (Adapt	75	Y
	AP2M1	Q96CW1	AP-2 complex subunit mu (AP-2 mu chain) (Adapter-related protein compl	18	Y
	AP3D1	O14617	AP-3 complex subunit delta-1 (AP-3 complex subunit delta) (Adapter-rel	17	Y
	AP3M1	Q9Y2T2	AP-3 complex subunit mu-1 (AP-3 adapter complex mu3A subunit) (Adapter	2	
	AP3S1	Q92572	AP-3 complex subunit sigma-1 (AP-3 complex subunit sigma-3A) (Adapter-	5	
Endocytosis	A8K9V7_HUMAN	A8K9V7	cDNA FLJ77207	75	
	AK1	P00568	Adenylate kinase isoenzyme 1 (AK 1) (EC 2.7.4.3) (EC 2.7.4.6) (ATP-AMP	9	Y
	ERLIN2	E5RHW4	Erlin-2 (Fragment)	3	
	EXT1	Q16394	Exostosin-1 (EC 2.4.1.224) (EC 2.4.1.225) (Glucuronosyl-N-acetylglucos	10	
	GLB1	P16278	Beta-galactosidase (EC 3.2.1.23) (Acid beta-galactosidase) (Lactase) (	17	Y
	GNB2L1	P63244	Guanine nucleotide-binding protein subunit beta-2-like 1 (Cell prolife	80	Y
	PLIN3	O60664	Perilipin-3 (47 kDa mannose 6-phosphate receptor-binding protein) (47	19	
	PSMD8	K7EJC1	26S proteasome non-ATPase regulatory subunit 8	9	Y
	Q59FU8_HUMAN	Q59FU8	Tumor necrosis factor receptor superfamily, member 6 isoform 1 variant	4	
	SCRN1	Q12765	Secernin-1	17	
	SF3A1	Q15459	Splicing factor 3A subunit 1 (SF3a120) (Spliceosome-associated protein	6	
	SNRNP200	O75643	U5 small nuclear ribonucleoprotein 200 kDa helicase (EC 3.6.4.13) (Act	81	

Table S3. Comparison of proteins identified in mesothelioma-derived exosomes from Hegmans et al 2004.

Gene Name	Protein Accession	Protein Description	Identified in current study (Supp Dataset)	Identified in current study - 631 common exosome list (Table S1)
ACTN4	O43707	Alpha-actinin-4	Y	Y
ANXA1	P04083	Annexin A1	Y	Y
ANXA2	P07355	Annexin A2	Y	Y
ANXA5	P08758	Annexin A5	Y	Y
ANXA6	A6NN80	Annexin A6	Y	Y
B2R6J2_HUMAN	B2R6J2	Villin 2	Y	Y
B3KTA3_HUMAN	B3KTA3	Fascin	Y	Y
CNP	P09543	2',3'-cyclic-nucleotide 3'-phosphodiesterase	Y	Y
DSP	P15924	Desmoplakin	Y	
ENO1	P06733	Alpha-enolase	Y	Y
FN1	P02751	Fibronectin	Y	
GAPDH	P04406	Glyceraldehyde-3-phosphate dehydrogenase	Y	Y
HLA-A	E9ABI2	MHC class I antigen (Fragment)	Y	
HLA-B	Q0ZAX5	MHC class I antigen (Fragment)	Y	
HSP90AA1	P07900	Heat shock protein HSP 90-alpha	Y	Y
ITGA3	P26006	Integrin alpha-3	Y	Y
MSN	P26038	Moesin	Y	Y
PKM	P14618	Pyruvate kinase PKM	Y	Y
TUBB	P07437	Tubulin beta chain	Y	Y

Table S4. Mesothelioma-specific exosome protein cancer signature.

Gene symbol	Gene Name	Protein Accession	Protein Description	Coverage (%)	SpC Combined (4 cell lines)	JO38 SpC	JU77 SpC	LO68 SpC	OLD1612 SpC	Vesiclepedia	Known_cancer-Exos*
KPYM_HUMAN	PKM	P14618	Pyruvate kinase PKM (EC 2.7.1.40) (Cytosolic thyroid hormone-binding p	61.2	824	114	120	192	414	Y	Y
ANXA2_HUMAN	ANXA2	P07355	Annexin A2 (Annexin II) (Annexin-2) (Calpactin I heavy chain) (Calpact	74.9	643	172	199	163	116	Y	Y
ACTB_HUMAN	ACTB	P60709	Actin, cytoplasmic 1 (Beta-actin) [Cleaved into: Actin, cytoplasmic 1,	49.1	640	173	121	133	223	Y	Y
B7ZLE5_HUMAN	FN1	P02751	Fibronectin	27.8	580		71	212	270	Y	Y
HSP7C_HUMAN	HSPA8	P11142	Heat shock cognate 71 kDa protein (Heat shock 70 kDa protein 8)	42.7	529	143	126	134	124	Y	Y
HS90B_HUMAN	HSP90AB1	P08238	Heat shock protein HSP 90-beta (HSP 90) (Heat shock 84 kDa) (HSP 84) (	40.6	498	141	102	136	121	Y	Y
HS90A_HUMAN	HSP90AA1	P07900	Heat shock protein HSP 90-alpha (Heat shock 86 kDa) (HSP 86) (HSP86) (	39.9	489	125	118	118	129	Y	Y
TBB4A_HUMAN	TUBB4A	P04350	Tubulin beta-4A chain (Tubulin 5 beta) (Tubulin beta-4 chain)	44.4	470	110	62	99	205	Y	Y
ACTN4_HUMAN	ACTN4	O43707	Alpha-actinin-4 (F-actin cross-linking protein) (Non-muscle alpha-acti	48.2	406	108	96	124	83	Y	Y
MOES_HUMAN	MSN	P26038	Moesin (Membrane-organizing extension spike protein)	53.6	405	149	94	39	128	Y	Y
MYH9_HUMAN	MYH9	P35579	Myosin-9 (Cellular myosin heavy chain, type A) (Myosin heavy chain 9)	35.3	397	194	63	28	118	Y	Y
ANXA5_HUMAN	ANXA5	P08758	Annexin A5 (Anchorin CII) (Annexin V) (Annexin-5) (Calphobindin I) (CB	66.3	351	100	90	96	71	Y	Y
HSP71_HUMAN	HSPA1A	P08107	Heat shock 70 kDa protein 1A/1B (Heat shock 70 kDa protein 1/2) (HSP70	39.6	327	62	53	113	98	Y	Y

ENOA_HUMAN	ENO1	P06733	Alpha-enolase (EC 4.2.1.11) (2-phospho-D-glycerate hydro-lyase) (C-myc	46.8	307	87	60	75	94	Y	Y
ANXA1_HUMAN	ANXA1	P04083	Annexin A1 (Annexin I) (Annexin-1) (Calpactin II) (Calpactin-2) (Chrom	61.6	268	104	35	77	56	Y	Y
G3P_HUMAN	GAPDH	P04406	Glyceraldehyde-3-phosphate dehydrogenase (GAPDH) (EC 1.2.1.12) (Peptid	43	262	95	25	79	69	Y	Y
ITA3_HUMAN	ITGA3	P26006	Integrin alpha-3 (CD49 antigen-like family member C) (FRP-2) (Galactop	21.9	227	79	64	13	73	Y	Y
A6NN80_HUMAN	ANXA6	A6NN80	Annexin A6	42.1	205	73	70	21	45	Y	Y
EDIL3_HUMAN	EDIL3	O43854	EGF-like repeat and discoidin I-like domain-containing protein 3 (Deve	36.5	146	9	61		77	Y	Y
CN37_HUMAN	CNP	P09543	2',3'-cyclic-nucleotide 3'-phosphodiesterase (CNP) (CNPase) (EC 3.1.4.	22.8	51	28	7	1	15	Y	Y
C9JV86_HUMAN	CD63	C9JV86	CD63 antigen	5.08	46	10	12	12	12	Y	Y
DESP_HUMAN	DSP	P15924	Desmoplakin (DP) (250/210 kDa paraneoplastic pemphigus antigen)	0.906	3		1	2		Y	Y
CPSM_HUMAN	CPS1	P31327	Carbamoyl-phosphate synthase [ammonia], mitochondrial (EC 6.3.4.16) (C	23.6	84			84		Y	
PAPP1_HUMAN	PAPPA	Q13219	Pappalysin-1 (EC 3.4.24.79) (Insulin-like growth factor-dependent IGF-	10.6	38			39		Y	
IFIT1_HUMAN	IFIT1	P09914	Interferon-induced protein with tetratricopeptide repeats 1 (IFIT-1) (	28.2	36	37				Y	
MX2_HUMAN	MX2	P20592	Interferon-induced GTP-binding protein Mx2 (Interferon-regulated resis	21.4	30	27	3			Y	
LAMA1_HUMAN	LAMA1	P25391	Laminin subunit alpha-1 (Laminin A chain) (Laminin-1 subunit alpha) (L	4.62	27	16	11			Y	
TYPH_HUMAN	TYMP	P19971	Thymidine phosphorylase (TP) (EC 2.4.2.4) (Gliostatin) (Platelet-deriv	26.8	26	26			1	Y	

ITLN1_HUMAN	ITLN1	Q8WWA0	Intelectin-1 (ITLN-1) (Endothelial lectin HL-1) (Galactofuranose-bindi	24	25		24			Y	
SAMD9_HUMAN	SAMD9	Q5K651	Sterile alpha motif domain-containing protein 9 (SAM domain-containing	4.41	23	17			6	Y	
NEK9_HUMAN	NEK9	Q8TD19	Serine/threonine-protein kinase Nek9 (EC 2.7.11.1) (Nercc1 kinase) (Ne	7.46	21	7		5	9	Y	
BCAT1_HUMAN	BCAT1	P54687	Branched-chain-amino-acid aminotransferase, cytosolic (BCAT(c)) (EC 2.	9.84	16	5	1		10	Y	
PROS_HUMAN	PROS1	P07225	Vitamin K-dependent protein S	8.14	13		12	1		Y	
CMPK2_HUMAN	CMPK2	Q5EBM0	UMP-CMP kinase 2, mitochondrial (EC 2.7.4.14) (Nucleoside-diphosphate	12.9	13	13				Y	
B4E1Z4_HUMAN	CFB	B4E1Z4	Complement factor B (Uncharacterized protein) (cDNA FLJ55673, highly s	3.24	12	6	3	4		Y	
MAMC2_HUMAN	MAMDC2	Q7Z304	MAM domain-containing protein 2 (MAM domain-containing proteoglycan) (	9.04	11	10		1		Y	
DOC10_HUMAN	DOCK10	Q96BY6	Dedicator of cytokinesis protein 10 (Zizimin-3)	3.44	10	6	3		1	Y	
HYCCI_HUMAN	FAM126A	Q9BYI3	Hyccin (Down-regulated by CTNNB1 protein A) (Protein FAM126A)	3.26	9	2	3		4	Y	
PDGFD_HUMAN	PDGFD	Q9GZP0	Platelet-derived growth factor D (PDGF-D) (Iris-expressed growth facto	8.92	9		3	6		Y	
AMGO2_HUMAN	AMIGO2	Q86SJ2	Amphoterin-induced protein 2 (AMIGO-2) (Alivin-1) (Differentially expr	8.62	8				8	Y	
SPAT5_HUMAN	SPATA5	Q8NB90	Spermatogenesis-associated protein 5 (ATPase family protein 2 homolog)	2.91	8				4	Y	
PIP_HUMAN	PIP	P12273	Prolactin-inducible protein (Gross cystic disease fluid protein 15) (G	26	8		1	7		Y	
CALB2_HUMAN	CALB2	P22676	Calretinin (CR) (29 kDa calbindin)	15.1	7				7	Y	

AP3S1_HUMAN	AP3S1	Q92572	AP-3 complex subunit sigma-1 (AP-3 complex subunit sigma-3A) (Adapter-	7.77	5	2			3	Y	
Q6NUJ9_HUMAN	SULF1	Q6NUJ9	SULF1 protein	4.5	5	1	4			Y	
ATS12_HUMAN	ADAMTS12	P58397	A disintegrin and metalloproteinase with thrombospondin motifs 12 (ADA	3.45	5	6				Y	
GBP1_HUMAN	GBP1	P32455	Interferon-induced guanylate-binding protein 1 (GTP-binding protein 1)	2.36	5	5				Y	
C9JYD7_HUMAN	GAL3ST1	C9JYD7	Galactosylceramide sulfotransferase (Fragment)	11.6	4			4		Y	
CBPE_HUMAN	CPE	P16870	Carboxypeptidase E (CPE) (EC 3.4.17.10) (Carboxypeptidase H) (CPH) (En	5.88	4		4			Y	
I6N6Q6_HUMAN	COX2	I6N6Q6	Cytochrome c oxidase subunit 2	7.05	4		4			Y	
ITA4_HUMAN	ITGA4	P13612	Integrin alpha-4 (CD49 antigen-like family member D) (Integrin alpha-I	3.78	4		4			Y	
B7Z4A7_HUMAN	EVI2B	B7Z4A7	Protein EVI2B (cDNA FLJ55224, highly similar to EVI2B protein)	6.05	4	4				Y	
PGDH_HUMAN	HPGD	P15428	15-hydroxyprostaglandin dehydrogenase [NAD(+)] (15-PGDH) (EC 1.1.1.141	10.5	3			3		Y	
RN215_HUMAN	RNF215	Q9Y6U7	RING finger protein 215	4.51	2			1	1	Y	
NID1_HUMAN	NID1	P14543	Nidogen-1 (NID-1) (Entactin)	2.17	2		1		1	Y	
NUB1_HUMAN	NUB1	Q9Y5A7	NEDD8 ultimate buster 1 (Negative regulator of ubiquitin-like proteins	2.28	2	1			1	Y	
A6NC48_HUMAN	BST1	A6NC48	ADP-ribosyl cyclase 2	3.9	2		2			Y	
OSMR_HUMAN	OSMR	Q99650	Oncostatin-M-specific receptor subunit beta (Interleukin-31 receptor s	1.63	2	2				Y	
SAM9L_HUMAN	SAMD9L	Q8IVG5	Sterile alpha motif domain-containing protein 9-like (SAM domain-conta	1.83	2	2				Y	

MTAP2_HUMAN	MAP2	P11137	Microtubule-associated protein 2 (MAP-2)	0.547	2				2	Y	
LPIN3_HUMAN	LPIN3	Q9BQK8	Phosphatidate phosphatase LPIN3 (EC 3.1.3.4) (Lipin-3) (Lipin-3-like)	1.65	2	2			1	Y	
MICA3_HUMAN	MICAL3	Q7RTP6	Protein-methionine sulfoxide oxidase MICAL3 (EC 1.14.13.-) (Molecule i	0.749	1				1	Y	
SPTN5_HUMAN	SPTBN5	Q9NRC6	Spectrin beta chain, non-erythrocytic 5 (Beta-V spectrin)	0.245	1		1			Y	
FBN2_HUMAN	FBN2	P35556	Fibrillin-2	0.412	1		1			Y	
Q8IWP6_HUMAN	Q8IWP6_HUMAN	Q8IWP6	Class IVb beta tubulin	48.8	544	127	71	116	239		
B4DVE1_HUMAN	B4DVE1_HUMAN	B4DVE1	cDNA FLJ53478, highly similar to Galectin-3-binding protein	36.6	465	178	94	135	60		
B3KPS3_HUMAN	B3KPS3_HUMAN	B3KPS3	cDNA FLJ32131 fis, clone PEBLM2000267, highly similar to Tubulin alpha	48.3	455	113	58	111	177		
B2R6J2_HUMAN	VIL2	B2R6J2	Villin 2 (ezrin) (VIL2)	39.6	238	78	52	34	78		
A8K7F6_HUMAN	A8K7F6_HUMAN	A8K7F6	cDNA FLJ78244, highly similar to Homo sapiens eukaryotic translation i	45.3	202	50	28	46	79		
A8K8D9_HUMAN	A8K8D9_HUMAN	A8K8D9	Glucose-6-phosphate 1-dehydrogenase (EC 1.1.1.49)	57.9	190	57	10	107	22		
A8K6Q8_HUMAN	A8K6Q8_HUMAN	A8K6Q8	cDNA FLJ75881, highly similar to Homo sapiens transferrin receptor (p9	35.9	181	73	45	20	45		
Q59E99_HUMAN	Q59E99_HUMAN	Q59E99	Thrombospondin 1 variant (Fragment)	21.6	164	39	22	88	16		
Q59GM9_HUMAN	Q59GM9_HUMAN	Q59GM9	Phosphorylase (EC 2.4.1.1) (Fragment)	28.7	139	20	4	53	66		
Q8N9M2_HUMAN	Q8N9M2_HUMAN	Q8N9M2	cDNA FLJ36887 fis, clone BNGH42005504, highly similar to 26S PROTEASOM	36.1	131	41	20	34	36		

B4DE36_HUMAN	B4DE36_HUMAN	B4DE36	Glucose-6-phosphate isomerase (EC 5.3.1.9)	31.7	122	22	17	48	36		
E1NZA1_HUMAN	PRIC295	E1NZA1	Peroxisome proliferator activated receptor interacting complex protein	16.5	119	25	4	27	65		
B2RCM2_HUMAN	B2RCM2_HUMAN	B2RCM2	cDNA, FLJ96156, highly similar to Homo sapiens leucyl-tRNA synthetase	24.1	117	27	6	14	71		
B2R5U1_HUMAN	B2R5U1_HUMAN	B2R5U1	cDNA, FLJ92620, highly similar to Homo sapiens staphylococcal nuclease	22.7	116	37	2	47	32		
B4DJ30_HUMAN	B4DJ30_HUMAN	B4DJ30	cDNA FLJ61290, highly similar to Neutral alpha-glucosidase AB	21.9	113	41	19	30	28		
B4DMC0_HUMAN	B4DMC0_HUMAN	B4DMC0	cDNA FLJ55341, highly similar to 116 kDa U5 small nuclear ribonucleopr	22.1	112	15	10	37	50		
A8K8U1_HUMAN	A8K8U1_HUMAN	A8K8U1	cDNA FLJ77762, highly similar to Homo sapiens cullin-associated and ne	17.4	112	39	27	36	11		
B3KTQ2_HUMAN	B3KTQ2_HUMAN	B3KTQ2	cDNA FLJ38589 fis, clone HCHON2010074, highly similar to LACTADHERIN	36.1	105	17	57	12	19		
B3KX11_HUMAN	B3KX11_HUMAN	B3KX11	cDNA FLJ44436 fis, clone UTERU2019706, highly similar to T-complex pro	29.7	104	29	11	34	31		
Q53HU0_HUMAN	Q53HU0_HUMAN	Q53HU0	Chaperonin containing TCP1, subunit 8 (Theta) variant (Fragment)	42.9	103	32	10	40	23		
A8K2T7_HUMAN	A8K2T7_HUMAN	A8K2T7	cDNA FLJ76780, highly similar to Homo sapiens epidermal growth factor	14.6	101	41	22	2	42		
A8K916_HUMAN	A8K916_HUMAN	A8K916	cDNA FLJ78481, highly similar to Homo sapiens adaptor-related protein	18	99	32	9	35	29		
B3KX72_HUMAN	B3KX72_HUMAN	B3KX72	cDNA FLJ44920 fis, clone BRAMY3011501, highly similar to Heterogeneous	22	97	24	9	12	54		
Q59G75_HUMAN	Q59G75_HUMAN	Q59G75	Isoleucyl-tRNA synthetase, cytoplasmic variant (Fragment)	16.3	96	27		25	46		

B2R8R5_HUMAN	B2R8R5_HUMAN	B2R8R5	cDNA, FLJ94025, highly similar to Homo sapiens tripartite motif-contai	20.4	94	2	3	39	50		
A8K6A5_HUMAN	A8K6A5_HUMAN	A8K6A5	cDNA FLJ77742, highly similar to Homo sapiens integrin, alpha 5 (fibro	13.9	92	41	19		34		
B4DLV7_HUMAN	B4DLV7_HUMAN	B4DLV7	cDNA FLJ60299, highly similar to Rab GDP dissociation inhibitor beta	38.3	91	32	22	8	30		
Q96MH4_HUMAN	Q96MH4_HUMAN	Q96MH4	cDNA FLJ32377 fis, clone SKMUS1000014, highly similar to Polyubiquitin	15.2	91	20	23	28	20		
Q53HV1_HUMAN	Q53HV1_HUMAN	Q53HV1	Ribosomal protein S4, X-linked X isoform variant (Fragment)	51.7	84	36	6	6	38		
Q59GB4_HUMAN	Q59GB4_HUMAN	Q59GB4	Dihydropyrimidinase-like 2 variant (Fragment)	25.5	84	22	13	23	24		
Q53EM5_HUMAN	Q53EM5_HUMAN	Q53EM5	Transketolase variant (Fragment)	29.7	84	10	13	56	8		
B2R5B3_HUMAN	B2R5B3_HUMAN	B2R5B3	Histone H2A	40.8	83	17	11	14	42		
B2R5M8_HUMAN	B2R5M8_HUMAN	B2R5M8	Isocitrate dehydrogenase [NADP] (EC 1.1.1.42)	34.1	83	27	10	39	8		
A8K492_HUMAN	A8K492_HUMAN	A8K492	cDNA FLJ76789, highly similar to Homo sapiens methionine-tRNA syntheta	14.3	78	25	5	21	29		
Q5U0C3_HUMAN	Q5U0C3_HUMAN	Q5U0C3	RAP1A, member of RAS oncogene family	42.4	78	29	15		22		
B2R9K8_HUMAN	B2R9K8_HUMAN	B2R9K8	cDNA, FLJ94440, highly similar to Homo sapiens chaperonin containing T	26	77	15	6	37	20		
B4DUQ1_HUMAN	B4DUQ1_HUMAN	B4DUQ1	cDNA FLJ54552, highly similar to Heterogeneous nuclear ribonucleoprote	28.5	75	17	11	23	25		
A8K9V7_HUMAN	A8K9V7_HUMAN	A8K9V7	cDNA FLJ77207	16.1	75	32	11	9	25		

B2R657_HUMAN	B2R657_HUMAN	B2R657	Annexin	22.5	75	20	15	20	23		
Q53HW2_HUMAN	Q53HW2_HUMAN	Q53HW2	Ribosomal protein P0 variant (Fragment)	27.1	74	17	12	20	25		
A8K9A4_HUMAN	A8K9A4_HUMAN	A8K9A4	cDNA FLJ75154, highly similar to Homo sapiens heterogeneous nuclear ri	26.5	72	20	10	17	28		
Q9BU08_HUMAN	Q9BU08_HUMAN	Q9BU08	Putative uncharacterized protein (Fragment)	26.5	71	18	5	32	18		
Q53HS0_HUMAN	Q53HS0_HUMAN	Q53HS0	Glutaminyl-tRNA synthetase variant (Fragment)	26.6	70	13	5	21	36		
Q53FT8_HUMAN	Q53FT8_HUMAN	Q53FT8	Proteasome subunit beta type (EC 3.4.25.1) (Fragment)	36.5	69	17	7	43	5		
B3KMX0_HUMAN	B3KMX0_HUMAN	B3KMX0	cDNA FLJ12837 fis, clone NT2RP2003228, highly similar to DNA replicati	22.6	67	1		34	33		
Q53GX7_HUMAN	Q53GX7_HUMAN	Q53GX7	Threonyl-tRNA synthetase variant (Fragment)	18.1	67	26	7	22	13		
B2RD27_HUMAN	B2RD27_HUMAN	B2RD27	cDNA, FLJ96428, highly similar to Homo sapiens proteasome (prosome, ma	24.1	65	23	3	17	21		
Q53HV2_HUMAN	Q53HV2_HUMAN	Q53HV2	Chaperonin containing TCP1, subunit 7 (Eta) variant (Fragment)	29.3	65	17	6	29	19		
Q9Y6E3_HUMAN	Q9Y6E3_HUMAN	Q9Y6E3	HSPC027	33.2	64	21	2	21	23		
Q53HH4_HUMAN	Q53HH4_HUMAN	Q53HH4	Ras-GTPase-activating protein SH3-domain-binding protein variant (Frag	18.2	64	17	9	19	20		
B4DE59_HUMAN	B4DE59_HUMAN	B4DE59	cDNA FLJ60424, highly similar to Junction plakoglobin	29.7	63	20	26	13	7		
Q59ER5_HUMAN	Q59ER5_HUMAN	Q59ER5	WD repeat-containing protein 1 isoform 1 variant (Fragment)	13.5	62	24	10	13	15		

B4DY23_HUMAN	B4DY23_HUMAN	B4DY23	cDNA FLJ61188, highly similar to Basigin	35.1	61	24	17	3	17		
B3KU62_HUMAN	B3KU62_HUMAN	B3KU62	cDNA FLJ39243 fis, clone OCBBF2008283, highly similar to Protein NDRG1	22.2	61	41	6	1	13		
B2R7C5_HUMAN	B2R7C5_HUMAN	B2R7C5	cDNA, FLJ93378, highly similar to Homo sapiens MCM3 minichromosome mai	19.9	60	2		28	31		
B3KTA3_HUMAN	B3KTA3_HUMAN	B3KTA3	Fascin	25.6	60	17	6	11	27		
Q59GL1_HUMAN	Q59GL1_HUMAN	Q59GL1	Synaptotagmin binding, cytoplasmic RNA interacting protein variant (Fr	26	59	14	3	12	32		
B4E1G2_HUMAN	B4E1G2_HUMAN	B4E1G2	Serine hydroxymethyltransferase (EC 2.1.2.1)	31.4	59	13	3	13	30		
B2RDW0_HUMAN	B2RDW0_HUMAN	B2RDW0	cDNA, FLJ96792, highly similar to Homo sapiens calmodulin 2 (phosphory	40.9	59	11	14	11	24		
A8K4T6_HUMAN	A8K4T6_HUMAN	A8K4T6	cDNA FLJ76282, highly similar to Homo sapiens proteasome (prosome, mac	19.2	58	16	4	16	23		
B2R7W4_HUMAN	B2R7W4_HUMAN	B2R7W4	cDNA, FLJ93632, highly similar to Homo sapiens heterogeneous nuclear r	24.5	57	16	2	8	34		
B3KY95_HUMAN	B3KY95_HUMAN	B3KY95	cDNA FLJ16143 fis, clone BRAMY2038516, highly similar to Protein disul	23.4	55	27	15	8	4		
B2RBA6_HUMAN	B2RBA6_HUMAN	B2RBA6	cDNA, FLJ95407, highly similar to Homo sapiens MCM7 minichromosome mai	33.1	53		1	16	37		
B4E2S3_HUMAN	B4E2S3_HUMAN	B4E2S3	cDNA FLJ56561	15.4	53	29	9		15		
B4DTS6_HUMAN	B4DTS6_HUMAN	B4DTS6	cDNA FLJ54117, highly similar to CD97 antigen	9.21	52	21	9		22		
Q9BTQ7_HUMAN	Q9BTQ7_HUMAN	Q9BTQ7	Similar to ribosomal protein L23 (Fragment)	34.3	51	18	14	6	14		

B3KQ33_HUMAN	B3KQ33_HUMAN	B3KQ33	cDNA FLJ32715 fis, clone TESTI2000784, highly similar to Importin-4	11.9	50	14		14	23		
A8K666_HUMAN	A8K666_HUMAN	A8K666	cDNA FLJ75460, highly similar to Homo sapiens phenylalanine-tRNA synth	20.2	50	10	4	15	21		
B7Z2N4_HUMAN	B7Z2N4_HUMAN	B7Z2N4	cDNA FLJ56074, highly similar to 150 kDa oxygen-regulated protein (Orp	19.1	50	41	1	2	6		
RTCB_HUMAN	RTCB	Q9Y3I0	tRNA-splicing ligase RtcB homolog (EC 6.5.1.3)	25	49	13	1	11	25		
B3KVN0_HUMAN	B3KVN0_HUMAN	B3KVN0	cDNA FLJ16785 fis, clone NT2RI2015342, highly similar to Solute carrie	9.13	49	26	3	7	13		
Q59EF6_HUMAN	Q59EF6_HUMAN	Q59EF6	Calpain 2, large [catalytic] subunit variant (Fragment)	19.5	48	12	1	2	33		
B2R6K4_HUMAN	B2R6K4_HUMAN	B2R6K4	cDNA, FLJ92996, highly similar to Homo sapiens guanine nucleotide bind	22.1	48	14	13	7	15		
A8K3C3_HUMAN	A8K3C3_HUMAN	A8K3C3	T-complex protein 1 subunit delta	20.4	48	12	10	14	12		
B4E175_HUMAN	B4E175_HUMAN	B4E175	cDNA FLJ54775, highly similar to Syntaxin-binding protein 2	21.5	48	25	16	1	5		
A8K6Y1_HUMAN	A8K6Y1_HUMAN	A8K6Y1	cDNA FLJ75526, highly similar to Homo sapiens proliferation-associated	32.7	46	13	1	17	16		
B2R6D7_HUMAN	B2R6D7_HUMAN	B2R6D7	cDNA, FLJ92904, highly similar to Homo sapiens casein kinase 2, alpha	22.5	46	12		22	14		
A8K525_HUMAN	A8K525_HUMAN	A8K525	cDNA FLJ76817, highly similar to Homo sapiens non-POU domain containin	22.3	45	9		15	24		
B4DUF1_HUMAN	B4DUF1_HUMAN	B4DUF1	cDNA FLJ59760, highly similar to 1,4-alpha-glucan branching enzyme (EC	18.3	44	19		1	28		
B7Z5J7_HUMAN	B7Z5J7_HUMAN	B7Z5J7	cDNA FLJ58682, highly similar to Vesicle-fusing ATPase (EC 3.6.4.6)	18.4	44	17	9	7	13		

A8K5Y7_HUMAN	A8K5Y7_HUMAN	A8K5Y7	cDNA FLJ78655, highly similar to Homo sapiens exportin 5 (XPO5), mRNA	11.9	41	3		12	26		
A8K529_HUMAN	A8K529_HUMAN	A8K529	cDNA FLJ76184, highly similar to Homo sapiens NOL1/NOP2/Sun domain fam	13.8	41	14		6	22		
B2RDE1_HUMAN	B2RDE1_HUMAN	B2RDE1	cDNA, FLJ96568, highly similar to Homo sapiens tropomyosin 3 (TPM3), m	28.6	41	28	4	3	6		
A8K590_HUMAN	A8K590_HUMAN	A8K590	cDNA FLJ77456, highly similar to Homo sapiens interleukin enhancer bin	20.5	40	9	6	6	21		
B3KXZ4_HUMAN	B3KXZ4_HUMAN	B3KXZ4	cDNA FLJ46429 fis, clone THYMU3014372, highly similar to DNA replicati	22.2	40			23	18		
A8K9P0_HUMAN	A8K9P0_HUMAN	A8K9P0	cDNA FLJ78413, highly similar to Homo sapiens albumin, mRNA	8.88	40	9	9	11	11		
B2R983_HUMAN	B2R983_HUMAN	B2R983	cDNA, FLJ94267, highly similar to Homo sapiens glutathione S-transfera	27	39	16	3	9	11		
B2R5W3_HUMAN	B2R5W3_HUMAN	B2R5W3	cDNA, FLJ92658, highly similar to Homo sapiens poly (ADP-ribose) polym	10.8	38	10	4	10	15		
B3KMB1_HUMAN	B3KMB1_HUMAN	B3KMB1	Structural maintenance of chromosomes protein	11.1	37	10		12	15		
B4DKN9_HUMAN	B4DKN9_HUMAN	B4DKN9	cDNA FLJ57740, highly similar to Transforming protein RhoA	27.7	37	10	12		14		
B2R858_HUMAN	B2R858_HUMAN	B2R858	cDNA, FLJ93750, Homo sapiens DEAD (Asp-Glu-Ala-Asp) box polypeptide 6	17.2	37	12		17	8		
B2R6J4_HUMAN	B2R6J4_HUMAN	B2R6J4	cDNA, FLJ92975, highly similar to Homo sapiens nucleosome assembly pro	17.3	37	9	8	12	8		
A3KC71_HUMAN	okuribin	A3KC71	Nuclear envelope protein okuribin	11.7	37	5	21	4	7		
Q6MZK8_HUMAN	DKFZp686K06110	Q6MZK8	Putative uncharacterized protein DKFZp686K06110	7.77	37			35			

B4DNN4_HUMAN	B4DNN4_HUMAN	B4DNN4	Ribonucleoside-diphosphate reductase (EC 1.17.4.1)	13	35	3	3	13	17		
B4DV28_HUMAN	B4DV28_HUMAN	B4DV28	cDNA FLJ54170, highly similar to Cytosolic nonspecific dipeptidase	16.8	35	13	7	5	10		
A8K6I4_HUMAN	A8K6I4_HUMAN	A8K6I4	cDNA FLJ76877, highly similar to Homo sapiens superkiller viralicidic	10.8	34	2		21	13		
B4DMB5_HUMAN	B4DMB5_HUMAN	B4DMB5	CTP synthase (EC 6.3.4.2) (UTP--ammonia ligase)	19.3	34	11		14	11		
B2RAK1_HUMAN	B2RAK1_HUMAN	B2RAK1	cDNA, FLJ94965, highly similar to Homo sapiens leucyl/cystinyl aminope	13.6	33	27	2		5		
Q53EP4_HUMAN	Q53EP4_HUMAN	Q53EP4	Ribophorin I variant (Fragment)	16.5	33	17	16				
A8K6Q6_HUMAN	A8K6Q6_HUMAN	A8K6Q6	cDNA FLJ78639	10.9	32	12	11		9		
A8K2N0_HUMAN	A8K2N0_HUMAN	A8K2N0	cDNA FLJ77835, highly similar to Homo sapiens complement component 1,	16	32	24	9	3			
B4DXV7_HUMAN	B4DXV7_HUMAN	B4DXV7	cDNA FLJ57661, highly similar to GMP synthase (glutamine-hydrolyzing)	16.3	31	4		11	18		
B2R6P3_HUMAN	B2R6P3_HUMAN	B2R6P3	cDNA, FLJ93047, highly similar to Homo sapiens matrix metallopeptidase	12.9	31		12		18		
A8K6A6_HUMAN	A8K6A6_HUMAN	A8K6A6	cDNA FLJ78619, highly similar to Homo sapiens melanoma cell adhesion m	15.8	31	17			13		
B3KQT9_HUMAN	B3KQT9_HUMAN	B3KQT9	cDNA PSEC0175 fis, clone OVARC1000169, highly similar to Protein disul	24.6	31	16	11	2	2		
B4DZF2_HUMAN	B4DZF2_HUMAN	B4DZF2	cDNA FLJ59571, highly similar to Eukaryotic translation initiation fac	10	29	5	1	7	19		
Q9H9B7_HUMAN	Q9H9B7_HUMAN	Q9H9B7	Coatomer subunit gamma	11	29	3	4	7	16		

B2RA70_HUMAN	B2RA70_HUMAN	B2RA70	cDNA, FLJ94729, highly similar to Homo sapiens v-yes-1 Yamaguchi sarco	14.2	29	7	6		14		
Q5JQ44_HUMAN	DKFZp547A0616	Q5JQ44	Putative uncharacterized protein DKFZp547A0616 (Fragment)	35.4	29	10	6	2	11		
B4DS24_HUMAN	B4DS24_HUMAN	B4DS24	cDNA FLJ50635, highly similar to ATP-dependent RNA helicase DDX19A (EC	20.8	28			8	21		
B4DI61_HUMAN	B4DI61_HUMAN	B4DI61	cDNA FLJ58182, highly similar to Protein CYR61	15.7	28	10	3		16		
A8K766_HUMAN	A8K766_HUMAN	A8K766	cDNA FLJ77343, highly similar to Homo sapiens electron-transfer-flavop	35.7	28	3		15	10		
A8KA19_HUMAN	A8KA19_HUMAN	A8KA19	cDNA FLJ75831, highly similar to Homo sapiens exportin, tRNA (nuclear	9.15	28	6	4	8	10		
A8K0T9_HUMAN	A8K0T9_HUMAN	A8K0T9	cDNA FLJ75422, highly similar to Homo sapiens capping protein (actin f	24.5	28	11	5	7	5		
A8K139_HUMAN	A8K139_HUMAN	A8K139	cDNA FLJ76744, highly similar to Homo sapiens L1 cell adhesion molecul	10.3	28	28					
A8K6H9_HUMAN	A8K6H9_HUMAN	A8K6H9	cDNA FLJ75876, highly similar to Homo sapiens solute carrier family 38	6.32	27	7	10		10		
B4DRT4_HUMAN	B4DRT4_HUMAN	B4DRT4	cDNA FLJ51535, highly similar to Phosphatidylethanolamine-binding prot	31.6	27	10	8	4	5		
A8KAJ3_HUMAN	A8KAJ3_HUMAN	A8KAJ3	cDNA FLJ77823, highly similar to Homo sapiens EGF-containing fibulin-l	12	27	14	10		2		
A8K690_HUMAN	A8K690_HUMAN	A8K690	cDNA FLJ76863, highly similar to Homo sapiens stress-induced-phosphopr	24.5	27	20	3	4	1		
B3KT14_HUMAN	B3KT14_HUMAN	B3KT14	cDNA FLJ37450 fis, clone BRAWH2010354, highly similar to Monoamine-sul	16.9	27	17	2	8			
B2RDF5_HUMAN	B2RDF5_HUMAN	B2RDF5	cDNA, FLJ96587, highly similar to Homo sapiens SUMO-1 activating enzym	12.8	26	2		12	13		
I1SRC5_HUMAN	I1SRC5_HUMAN	I1SRC5	UBE2L3/KRAS fusion protein	26	26		7		13		

B2RE34_HUMAN	B2RE34_HUMAN	B2RE34	cDNA, FLJ96901, highly similar to Homo sapiens Rac GTPase activating p	11.7	26	11	12	1	4		
Q59EK6_HUMAN	Q59EK6_HUMAN	Q59EK6	TNF receptor-associated protein 1 variant (Fragment)	15.6	26	13		9	3		
B3KRF9_HUMAN	B3KRF9_HUMAN	B3KRF9	cDNA FLJ34156 fis, clone FCBBF3013266, highly similar to Tsukushi (Leu	21.5	26	11		15			
B4DPM0_HUMAN	B4DPM0_HUMAN	B4DPM0	Pyruvate kinase (EC 2.7.1.40)	4.51	26	5	5				
B2R9S4_HUMAN	B2R9S4_HUMAN	B2R9S4	cDNA, FLJ94534, highly similar to Homo sapiens capping protein (actin	17.2	25	7	1	1	17		
A8K4H1_HUMAN	A8K4H1_HUMAN	A8K4H1	cDNA FLJ78268, highly similar to Homo sapiens fusion (involved in t(12	9.33	25	4		8	14		
Q4LE69_HUMAN	PIK4CA	Q4LE69	PIK4CA variant protein (Fragment)	5.61	25	15	3		8		
A8KA84_HUMAN	A8KA84_HUMAN	A8KA84	cDNA FLJ78682, highly similar to Homo sapiens 2'-5'-oligoadenylate syn	13	25	26					
A8K6X3_HUMAN	A8K6X3_HUMAN	A8K6X3	cDNA FLJ78679, highly similar to Homo sapiens DEAD (Asp-Glu-Ala-Asp) b	10.9	24			3	25		
B4DQ93_HUMAN	B4DQ93_HUMAN	B4DQ93	cDNA FLJ52996, highly similar to Syntenin-1	16.9	24	1	7	10	7		
A8K607_HUMAN	A8K607_HUMAN	A8K607	cDNA FLJ76855, highly similar to Homo sapiens exportin 7 (XPO7), mRNA	12.2	24	3	1	17	4		
H3BQZ7_HUMAN	hCG_2044799	H3BQZ7	HCG2044799 (Uncharacterized protein)	12.6	23	4		2	17		
Q53F48_HUMAN	Q53F48_HUMAN	Q53F48	Heterogeneous nuclear ribonucleoprotein H3 isoform a variant (Fragment	8.38	23	4		9	11		
A8K5W7_HUMAN	A8K5W7_HUMAN	A8K5W7	cDNA FLJ75180, highly similar to Homo sapiens mitochondrial isoleucine	10.9	23	7		11	6		

B7Z2A1_HUMAN	B7Z2A1_HUMAN	B7Z2A1	cDNA FLJ56068, highly similar to DNA damage-binding protein 1	12.7	23		1	21	1		
B4DJB4_HUMAN	B4DJB4_HUMAN	B4DJB4	cDNA FLJ55931, highly similar to Isocitrate dehydrogenase	19.9	22		1	4	17		
B4DRW3_HUMAN	B4DRW3_HUMAN	B4DRW3	cDNA FLJ57180, highly similar to TAR DNA-binding protein 43	12.7	22	6		9	7		
B4DSN3_HUMAN	B4DSN3_HUMAN	B4DSN3	cDNA FLJ60345, highly similar to Protein transport protein Sec24C	6.36	22	8	4	7	4		
Q9BQQ5_HUMAN	L27a	Q9BQQ5	Ribosomal protein L27a	51.9	21	7	2	2	11		
B2R4D8_HUMAN	B2R4D8_HUMAN	B2R4D8	60S ribosomal protein L27	30.9	21	8		4	9		
A8K5M4_HUMAN	A8K5M4_HUMAN	A8K5M4	cDNA FLJ75088, highly similar to Homo sapiens p21 (CDKN1A)-activated k	17.6	21	9	3		9		
B4E1U9_HUMAN	B4E1U9_HUMAN	B4E1U9	cDNA FLJ54776, highly similar to Cell division control protein 42 homo	17.8	21	8	4	3	6		
Q59F71_HUMAN	Q59F71_HUMAN	Q59F71	Bone morphogenetic protein 1 isoform 1, variant (Fragment)	3.99	21	8	2	6	5		
A8K883_HUMAN	A8K883_HUMAN	A8K883	cDNA FLJ77590, highly similar to Homo sapiens leucine rich repeat cont	9.54	21	7	7		4		
Q8NBH6_HUMAN	Q8NBH6_HUMAN	Q8NBH6	cDNA PSEC0266 fis, clone NT2RP3003649, highly similar to Homo sapiens	10.3	21	6		15			
B4E3A8_HUMAN	B4E3A8_HUMAN	B4E3A8	cDNA FLJ53963, highly similar to Leukocyte elastase inhibitor	22	21	16	3	2			
B4DLC3_HUMAN	B4DLC3_HUMAN	B4DLC3	Claudin	18.7	21	10	10	1			
B2R769_HUMAN	B2R769_HUMAN	B2R769	cDNA, FLJ93308, highly similar to Homo sapiens leukocyte-derived argin	10.6	21	24					

B2R4C0_HUMAN	B2R4C0_HUMAN	B2R4C0	60S ribosomal protein L18a	37.5	20	10			12		
A8K4W5_HUMAN	A8K4W5_HUMAN	A8K4W5	cDNA FLJ76813, highly similar to Homo sapiens acetyl-Coenzyme A acetyl	12.6	20	14	1	4	1		
IFIT2_HUMAN	IFIT2	P09913	Interferon-induced protein with tetratricopeptide repeats 2 (IFIT-2) (	22.9	20	22					
B2RBE5_HUMAN	B2RBE5_HUMAN	B2RBE5	cDNA, FLJ95468, highly similar to Homo sapiens transcriptional coactiv	10.3	19	3		1	16		
Q8N1H4_HUMAN	Q8N1H4_HUMAN	Q8N1H4	cDNA FLJ40872 fis, clone TUTER2000283, highly similar to Homo sapiens	15.5	19	7	2		10		
Q53H34_HUMAN	Q53H34_HUMAN	Q53H34	Ribosomal protein L13a variant (Fragment)	22.7	19	9	1		10		
B2RD51_HUMAN	B2RD51_HUMAN	B2RD51	cDNA, FLJ96455, highly similar to Homo sapiens Down syndrome critical	13.9	19	5	2	5	7		
Q53GW1_HUMAN	Q53GW1_HUMAN	Q53GW1	Vesicle transport-related protein isoform a variant (Fragment)	18.1	19	17		1	1		
B3KUB6_HUMAN	B3KUB6_HUMAN	B3KUB6	cDNA FLJ39529 fis, clone PUAEN2004067, highly similar to Band 4.1-like	10.9	18	6		4	9		
A8K4G7_HUMAN	A8K4G7_HUMAN	A8K4G7	cDNA FLJ78528, highly similar to Homo sapiens vacuolar protein sorting	16.7	18	5	2	5	6		
B4E0U6_HUMAN	B4E0U6_HUMAN	B4E0U6	cDNA FLJ53687, highly similar to Hsc70-interacting protein	12.3	18	7	4	1	6		
GT251_HUMAN	COLGALT1	Q8NBJ5	Procollagen galactosyltransferase 1 (EC 2.4.1.50) (Collagen beta(1-O)g	7.72	18	11	1		6		
Q68E05_HUMAN	DKFZp686A1765	Q68E05	Putative uncharacterized protein DKFZp686A1765 (Tumor protein D52-like	23.1	18	11	1		6		
B7Z992_HUMAN	B7Z992_HUMAN	B7Z992	cDNA FLJ53698, highly similar to Gelsolin	6.43	18	2	8	3	5		

B3KRN4_HUMAN	B3KRN4_HUMAN	B3KRN4	cDNA FLJ34625 fis, clone KIDNE2015244, highly similar to Serine protea	17.2	18	4		12	3		
B4DPD5_HUMAN	B4DPD5_HUMAN	B4DPD5	cDNA FLJ56307, highly similar to Ubiquitin thioesterase protein OTUB1	21.8	18	9		6	3		
A8K5U9_HUMAN	A8K5U9_HUMAN	A8K5U9	cDNA FLJ75056, highly similar to Homo sapiens phosphatidylinositol bin	8.44	18	6	7	2	3		
B4DE48_HUMAN	B4DE48_HUMAN	B4DE48	cDNA FLJ53824, highly similar to Sorting nexin-17	10.9	18	7	9	1			
Q53G21_HUMAN	Q53G21_HUMAN	Q53G21	Small nuclear ribonucleoprotein polypeptide A' variant (Fragment)	18.8	17			4	13		
B3KM86_HUMAN	B3KM86_HUMAN	B3KM86	cDNA FLJ10524 fis, clone NT2RP2000880, highly similar to Eukaryotic tr	10.3	17	3		2	13		
Q53HL1_HUMAN	Q53HL1_HUMAN	Q53HL1	Myosin regulatory light chain MRCL3 variant (Fragment)	18.7	17	4	3	3	8		
A8K2I7_HUMAN	A8K2I7_HUMAN	A8K2I7	cDNA FLJ76072, highly similar to Homo sapiens GIPC PDZ domain containi	23.1	17	7	2		8		
B4DSD7_HUMAN	B4DSD7_HUMAN	B4DSD7	cDNA FLJ55863, highly similar to Serine/threonine-protein phosphatase	8.77	17		5	3	7		
HNRDL_HUMAN	HNRNPDL	O14979	Heterogeneous nuclear ribonucleoprotein D-like (hnRNP D-like) (hnRNP D	10	17	7	1	3	6		
B7Z7F0_HUMAN	B7Z7F0_HUMAN	B7Z7F0	cDNA FLJ56420, highly similar to Aspartyl aminopeptidase (EC 3.4.11.21	7.71	17	5	2	7	3		
B2RAH7_HUMAN	B2RAH7_HUMAN	B2RAH7	cDNA, FLJ94921, highly similar to Homo sapiens prolyl endopeptidase (P	6.48	17	7	1	6	3		
B3KPN7_HUMAN	B3KPN7_HUMAN	B3KPN7	cDNA FLJ32002 fis, clone NT2RP7009394, highly similar to Exocyst compl	6.17	17	7	8		2		
B4DHJ3_HUMAN	B4DHJ3_HUMAN	B4DHJ3	DNA-directed RNA polymerase (EC 2.7.7.6)	10	16	1		2	13		

Q53GF0_HUMAN	Q53GF0_HUMAN	Q53GF0	Cytidine 5'-monophosphate N-acetylneuraminic acid synthetase variant (	14.1	16	2		2	12		
Q59F99_HUMAN	Q59F99_HUMAN	Q59F99	Staufen isoform b variant (Fragment)	8.46	16	6			10		
Q59EH7_HUMAN	Q59EH7_HUMAN	Q59EH7	DnaJ (Hsp40) homolog, subfamily C, member 7 variant (Fragment)	9.32	16	5		5	6		
B4DF70_HUMAN	B4DF70_HUMAN	B4DF70	cDNA FLJ60461, highly similar to Peroxiredoxin-2 (EC 1.11.1.15)	27.3	16	2	8	1	6		
A8K8A4_HUMAN	A8K8A4_HUMAN	A8K8A4	cDNA FLJ77640, highly similar to Homo sapiens copine II (CPNE2), mRNA	7.3	16	6	4	2	4		
B2RBH6_HUMAN	B2RBH6_HUMAN	B2RBH6	cDNA, FLJ95513, highly similar to Homo sapiens cyclin fold protein 1 (	12.6	16	9	4		4		
B4DM33_HUMAN	B4DM33_HUMAN	B4DM33	cDNA FLJ52068, highly similar to Microtubule-associated protein RP/EB	13.9	16	8	4	4	3		
B4DKZ2_HUMAN	B4DKZ2_HUMAN	B4DKZ2	cDNA FLJ56653, highly similar to Ribosomal protein S6 kinase alpha-3 (	11	16	4		10	2		
B2R514_HUMAN	B2R514_HUMAN	B2R514	cDNA, FLJ92300, Homo sapiens COP9 subunit 6 (MOV34 homolog, 34 kD) (CO	10.4	16	4		12			
Q53HF3_HUMAN	Q53HF3_HUMAN	Q53HF3	Galactosidase, alpha variant (Fragment)	9.56	16	7		10			
Q59EJ5_HUMAN	Q59EJ5_HUMAN	Q59EJ5	Glutathione S-transferase M3 variant (Fragment)	26.4	16	16					
A8K6K7_HUMAN	A8K6K7_HUMAN	A8K6K7	cDNA FLJ76881, highly similar to Homo sapiens glycogen synthase 1 (mus	5.83	15	1		2	12		
B3KRM2_HUMAN	B3KRM2_HUMAN	B3KRM2	Serine/threonine-protein phosphatase (EC 3.1.3.16)	12.9	15	3		1	11		
B4DZJ7_HUMAN	B4DZJ7_HUMAN	B4DZJ7	Transcription elongation factor SPT5	7.14	15			6	9		

A8K3W4_HUMAN	A8K3W4_HUMAN	A8K3W4	cDNA FLJ75163, highly similar to Homo sapiens heterogeneous nuclear ri	7.28	15			8	8		
B4DN89_HUMAN	SFRS2	B4DN89	Serine/arginine-rich-splicing factor 2 (Splicing factor, arginine/seri	19.6	15	6		1	8		
B3KRY3_HUMAN	B3KRY3_HUMAN	B3KRY3	cDNA FLJ35079 fis, clone PLACE6005283, highly similar to Lysosome-asso	9.87	15	6	5		4		
Q53GF9_HUMAN	Q53GF9_HUMAN	Q53GF9	Full-length cDNA 5-PRIME end of clone CS0DF013YM24 of Fetal brain of H	16	15	9	3		3		
Q59GU6_HUMAN	Q59GU6_HUMAN	Q59GU6	Sorting nexin 1 isoform a variant (Fragment)	12.7	15	1	6	6	1		
A8K556_HUMAN	A8K556_HUMAN	A8K556	cDNA FLJ78217	5.6	15	2	8	4	1		
B3KUZ8_HUMAN	B3KUZ8_HUMAN	B3KUZ8	Aspartate aminotransferase (EC 2.6.1.1)	11.3	15	5	3	7			
B4DND4_HUMAN	B4DND4_HUMAN	B4DND4	cDNA FLJ50588, highly similar to Gamma-glutamyltransferase 5 (EC 2.3.2	18.4	15	16					
B2R888_HUMAN	B2R888_HUMAN	B2R888	Monocyte differentiation antigen CD14 (Myeloid cell-specific leucine-r	14.1	15	15					
B4DYK6_HUMAN	B4DYK6_HUMAN	B4DYK6	cDNA FLJ56887, highly similar to Homo sapiens guanine nucleotide bindi	10.1	14	1			13		
A8K7A4_HUMAN	A8K7A4_HUMAN	A8K7A4	cDNA FLJ76904, highly similar to Homo sapiens methionine adenosyltrans	7.74	14	1	2	6	5		
B2R5I8_HUMAN	B2R5I8_HUMAN	B2R5I8	cDNA, FLJ92490, highly similar to Homo sapiens RAB32, member RAS oncog	9.78	14	2	3	4	5		
B4DEK8_HUMAN	B4DEK8_HUMAN	B4DEK8	cDNA FLJ53721, highly similar to Tetraspanin-3	7.44	14	3	3	3	5		
B2RCJ6_HUMAN	B2RCJ6_HUMAN	B2RCJ6	cDNA, FLJ96114, highly similar to Homo sapiens bromodomain and WD repe	3.1	14	6		4	4		

A8K0J3_HUMAN	A8K0J3_HUMAN	A8K0J3	cDNA FLJ76732, highly similar to Homo sapiens TAO kinase 3 (TAOK3), mR	5.57	14	7	1		4		
H0Y626_HUMAN	H0Y626_HUMAN	H0Y626	Uncharacterized protein	5.67	14			12	2		
PSG4_HUMAN	PSG4	Q00888	Pregnancy-specific beta-1-glycoprotein 4 (PS-beta-G-4) (PSBG-4) (Pregn	11.9	14		15				
Q5HYG7_HUMAN	DKFZp686M24262	Q5HYG7	Putative uncharacterized protein DKFZp686M24262	20	13				13		
B2R7C2_HUMAN	B2R7C2_HUMAN	B2R7C2	cDNA, FLJ93375, highly similar to Homo sapiens ZW10, kinetochore assoc	6.8	13		1	2	11		
B7Z6F7_HUMAN	B7Z6F7_HUMAN	B7Z6F7	cDNA FLJ61705, highly similar to Symplekin	4.4	13			3	10		
B3KTJ9_HUMAN	B3KTJ9_HUMAN	B3KTJ9	cDNA FLJ38393 fis, clone FEBRA2007212	9.53	13			5	9		
B2R7M3_HUMAN	B2R7M3_HUMAN	B2R7M3	cDNA, FLJ93510, highly similar to Homo sapiens JTV1 gene (JTV1), mRNA	12.2	13	8	1		4		
B2R6F5_HUMAN	B2R6F5_HUMAN	B2R6F5	cDNA, FLJ92928, highly similar to Homo sapiens retinitis pigmentosa 2	9.71	13	9	1		3		
B7Z670_HUMAN	B7Z670_HUMAN	B7Z670	cDNA FLJ61703, highly similar to Neuronal cell adhesion molecule (Frag	5.98	13			13			
B4DDM5_HUMAN	B4DDM5_HUMAN	B4DDM5	cDNA FLJ53298, highly similar to Peroxisomal multifunctional enzyme ty	6	13	3	5	4			
B3KQQ3_HUMAN	B3KQQ3_HUMAN	B3KQQ3	cDNA PSEC0016 fis, clone NT2RM1001076, highly similar to Procollagen-l	12.6	13	9		4			
B1PL87_HUMAN	B1PL87_HUMAN	B1PL87	Bid (Fragment)	22.1	13	13					
B4DZC0_HUMAN	B4DZC0_HUMAN	B4DZC0	cDNA FLJ51771, highly similar to SWI/SNF-related matrix-associatedacti	4.72	12				13		

O76046_HUMAN	SKI2W	O76046	Putative RNA helicase Ski2w	7.87	12	1			12		
A8K548_HUMAN	A8K548_HUMAN	A8K548	cDNA FLJ75008, highly similar to Homo sapiens proline-, glutamic acid-	4.6	12				12		
A8K586_HUMAN	A8K586_HUMAN	A8K586	cDNA FLJ76822, highly similar to Homo sapiens adaptor-related protein	6.03	12		2	3	7		
A8K8K1_HUMAN	A8K8K1_HUMAN	A8K8K1	cDNA FLJ76936, highly similar to Homo sapiens RNA terminal phosphate c	10.1	12	5	1		7		
B4DP09_HUMAN	B4DP09_HUMAN	B4DP09	cDNA FLJ53072, highly similar to Calponin-3	18.4	12	4	1		7		
B4DFP1_HUMAN	B4DFP1_HUMAN	B4DFP1	cDNA FLJ51818, highly similar to Phosphoglucomutase-1 (EC 5.4.2.2)	11.7	12	10	1		2		
A8K3E4_HUMAN	A8K3E4_HUMAN	A8K3E4	cDNA FLJ78367, highly similar to Homo sapiens fibrinogen, A alpha poly	11.8	12			12			
B2RCX0_HUMAN	B2RCX0_HUMAN	B2RCX0	cDNA, FLJ96345, Homo sapiens SET translocation (myeloid leukemia-assoc	13.4	12	1	5	6			
B3KTX4_HUMAN	B3KTX4_HUMAN	B3KTX4	cDNA FLJ38923 fis, clone NT2NE2011823, highly similar to Dynactin subu	16.7	12	9		3			
Q53HG7_HUMAN	Q53HG7_HUMAN	Q53HG7	Cortactin isoform a variant (Fragment)	8	12	3	7	2			
A8K5J8_HUMAN	A8K5J8_HUMAN	A8K5J8	cDNA FLJ75066, highly similar to Homo sapiens complement component 1,	7.38	12	10	2	1			
A8KAP9_HUMAN	A8KAP9_HUMAN	A8KAP9	cDNA FLJ78448, highly similar to Homo sapiens argininosuccinate synthe	9.22	12	15					
A8KAH7_HUMAN	A8KAH7_HUMAN	A8KAH7	cDNA FLJ75444, highly similar to Homo sapiens protein kinase, cAMP-dep	17.6	11				11		
B4DUI2_HUMAN	B4DUI2_HUMAN	B4DUI2	cDNA FLJ61415, highly similar to Protein kinase C and casein kinase su	9.26	11	1	1		10		

A8K4I1_HUMAN	A8K4I1_HUMAN	A8K4I1	cDNA FLJ75404	10.1	11		1		10		
B2R7B5_HUMAN	B2R7B5_HUMAN	B2R7B5	cDNA, FLJ93365, highly similar to Homo sapiens KH domain containing, R	5.42	11	1		1	9		
B3KMR5_HUMAN	B3KMR5_HUMAN	B3KMR5	cDNA FLJ12434 fis, clone NT2RM1000037, highly similar to Homo sapiens	3.47	11	2	1		8		
B4DF00_HUMAN	B4DF00_HUMAN	B4DF00	cDNA FLJ53308, highly similar to 2-oxoglutarate dehydrogenase E1 compo	2.67	11	3		6	2		
B4DLB8_HUMAN	B4DLB8_HUMAN	B4DLB8	cDNA FLJ52205, highly similar to Beta-1,4-galactosyltransferase 1 (EC	9.3	11	6	1	3	2		
B4DSX6_HUMAN	B4DSX6_HUMAN	B4DSX6	cDNA FLJ57427, highly similar to Glycogenin-1 (EC 2.4.1.186)	15.5	11	9	1		2		
B2R6U8_HUMAN	B2R6U8_HUMAN	B2R6U8	cDNA, FLJ93125, highly similar to Homo sapiens cleavage and polyadenyl	23.8	11	2		9			
A8K2L6_HUMAN	A8K2L6_HUMAN	A8K2L6	Annexin	17.1	11	2		9			
B2RAQ9_HUMAN	B2RAQ9_HUMAN	B2RAQ9	Proteasome subunit beta type (EC 3.4.25.1)	9.03	11	2		9			
B7Z4S4_HUMAN	B7Z4S4_HUMAN	B7Z4S4	cDNA FLJ52567, highly similar to Renin receptor	13.5	11	7		4			
B4DN31_HUMAN	B4DN31_HUMAN	B4DN31	cDNA FLJ55809	11.5	11		11				
B3KQJ0_HUMAN	B3KQJ0_HUMAN	B3KQJ0	cDNA FLJ90530 fis, clone NT2RP4002187, highly similar to Homo sapiens	9.94	11	6	5				
B2RE46_HUMAN	B2RE46_HUMAN	B2RE46	cDNA, FLJ96923, highly similar to Homo sapiens ribophorin II (RPN2), m	6.81	11	7	4				
B2R5J8_HUMAN	B2R5J8_HUMAN	B2R5J8	cDNA, FLJ92501, highly similar to Homo sapiens chemokine (C-C motif) l	35.2	11	11					

H0UI95_HUMAN	hCG_2044836	H0UI95	HCG2044836, isoform CRA_a	12.6	11	11					
B3KY63_HUMAN	B3KY63_HUMAN	B3KY63	cDNA FLJ16830 fis, clone UTERU3022536, highly similar to Chromodomain	3.39	10				10		
A8K2X4_HUMAN	A8K2X4_HUMAN	A8K2X4	cDNA FLJ75401, highly similar to Homo sapiens endoglin (Osler-Rendu-We	11.2	10				10		
B3KMC9_HUMAN	B3KMC9_HUMAN	B3KMC9	cDNA FLJ10711 fis, clone NT2RP3000917, highly similar to 5'-3' exoribo	8.32	10			3	8		
B7Z7Y3_HUMAN	B7Z7Y3_HUMAN	B7Z7Y3	cDNA FLJ61618, highly similar to Discoidin, CUB and LCCL domain-contai	6.13	10	1	2		8		
A8K4T9_HUMAN	A8K4T9_HUMAN	A8K4T9	cDNA FLJ77421, highly similar to Homo sapiens autoantigen p542 mRNA	18.2	10	1	1		8		
B4DJW8_HUMAN	B4DJW8_HUMAN	B4DJW8	cDNA FLJ55697, highly similar to Protein transport protein Sec23B	8.01	10	3			7		
A8K897_HUMAN	A8K897_HUMAN	A8K897	cDNA FLJ78686, highly similar to Homo sapiens nucleoporin 93kDa (NUP93	5.86	10	1		3	6		
A8K0C2_HUMAN	A8K0C2_HUMAN	A8K0C2	cDNA FLJ75950	11	10		1	4	5		
B2RDD7_HUMAN	B2RDD7_HUMAN	B2RDD7	cDNA, FLJ96564, highly similar to Homo sapiens SKB1 homolog (S. pombe)	4.4	10	3		3	5		
B2R9L6_HUMAN	B2R9L6_HUMAN	B2R9L6	cDNA, FLJ94450, highly similar to Homo sapiens cyclin-dependent kinase	6.45	10				5		
CLU_HUMAN	CLUH	O75153	Clustered mitochondria protein homolog	2.22	10	2		4	4		
B4DW31_HUMAN	B4DW31_HUMAN	B4DW31	cDNA FLJ54186, highly similar to Nuclear receptor-binding protein	8.16	10	3		3	4		
PIPSL_HUMAN	PIPSL	A2A3N6	Putative PIP5K1A and PSMD4-like protein (PIP5K1A-PSMD4)	3.13	10	3		3	4		

B3KSP9_HUMAN	B3KSP9_HUMAN	B3KSP9	cDNA FLJ36765 fis, clone 3NB691000191	6.45	10	4		3	3		
B2RDT8_HUMAN	B2RDT8_HUMAN	B2RDT8	cDNA, FLJ96764, highly similar to Homo sapiens sorting nexin 8 (SNX8),	11.8	10	4	2	1	3		
B2R6N0_HUMAN	B2R6N0_HUMAN	B2R6N0	cDNA, FLJ93029, highly similar to Homo sapiens COP9 constitutive photo	10.4	10	5		3	2		
B2R679_HUMAN	B2R679_HUMAN	B2R679	cDNA, FLJ92825, highly similar to Homo sapiens SAR1a gene homolog 1 (S	11.6	10	3	3	2	2		
A0MSJ5_HUMAN	A0MSJ5_HUMAN	A0MSJ5	Sodium-ascorbic acid transporter 2	6.77	10	9			1		
B4DVA7_HUMAN	B4DVA7_HUMAN	B4DVA7	Beta-hexosaminidase (EC 3.2.1.52)	8.33	10	1	8				
B7Z5L6_HUMAN	B7Z5L6_HUMAN	B7Z5L6	cDNA FLJ54723, highly similar to Poly (ADP-ribose) polymerase 9 (EC 2.	8.37	10	12					
MROH1_HUMAN	MROH1	Q8NDA8	Maestro heat-like repeat-containing protein family member 1 (HEAT repe	3.6	10	10					
Q7Z3K9_HUMAN	DKFZp781L0540	Q7Z3K9	Putative uncharacterized protein DKFZp781L0540 (Fragment)	4.51	9	1		1	8		
A8K6G9_HUMAN	A8K6G9_HUMAN	A8K6G9	cDNA FLJ76871, highly similar to Homo sapiens DEAH (Asp-Glu-Ala-His) b	4.97	9			1	8		
B3KTC9_HUMAN	B3KTC9_HUMAN	B3KTC9	cDNA FLJ38075 fis, clone CTONG2015815, highly similar to F-box/LRR-rep	4.52	9	1			8		
B2R960_HUMAN	B2R960_HUMAN	B2R960	cDNA, FLJ94230, highly similar to Homo sapiens thioredoxin-like 1 (TXN	11.4	9	1		1	7		
B2RDP6_HUMAN	B2RDP6_HUMAN	B2RDP6	cDNA, FLJ96709, highly similar to Homo sapiens glutamate rich WD repea	12.6	9			3	6		
Q8WVX7_HUMAN	Q8WVX7_HUMAN	Q8WVX7	Ribosomal protein S19 (Fragment)	21	9	2		1	6		

B4DQE1_HUMAN	B4DQE1_HUMAN	B4DQE1	Annexin	13.7	9	1	3		5		
B4DM85_HUMAN	B4DM85_HUMAN	B4DM85	cDNA FLJ56002, highly similar to Kinesin-like protein KIF2	3.23	9	4	1		5		
B4DG42_HUMAN	B4DG42_HUMAN	B4DG42	cDNA FLJ53753, highly similar to Myeloid-associated differentiation ma	9.82	9	2	4	1	2		
B4E2I9_HUMAN	B4E2I9_HUMAN	B4E2I9	cDNA FLJ55486, highly similar to Semaphorin-3C	6.89	9	7			2		
B7Z2B0_HUMAN	B7Z2B0_HUMAN	B7Z2B0	cDNA FLJ53470, highly similar to Calcium/calmodulin-dependent protein	6.75	9	3	3	2	1		
OASL_HUMAN	OASL	Q15646	2'-5'-oligoadenylate synthase-like protein (2'-5'-OAS-related protein)	12.8	9	9					
E5KND7_HUMAN	E5KND7_HUMAN	E5KND7	Mitochondrial elongation factor G	4.39	8				10		
B3KM58_HUMAN	B3KM58_HUMAN	B3KM58	cDNA FLJ10358 fis, clone NT2RM2001238, highly similar to Glutaminase k	11.1	8				8		
A8KAQ5_HUMAN	A8KAQ5_HUMAN	A8KAQ5	cDNA FLJ77404, highly similar to Homo sapiens small nuclear ribonucleo	8.47	8	1		1	7		
A4GW21_HUMAN	A4GW21_HUMAN	A4GW21	Programmed cell death ligand 2	10.6	8		1		7		
B4DYP7_HUMAN	B4DYP7_HUMAN	B4DYP7	cDNA FLJ55435, highly similar to Gamma-tubulin complex component 3	5.02	8	1			7		
Q53EL1_HUMAN	Q53EL1_HUMAN	Q53EL1	Protein KIAA0196 variant (Fragment)	4.64	8	5	2		1		
A8K5T0_HUMAN	A8K5T0_HUMAN	A8K5T0	cDNA FLJ75416, highly similar to Homo sapiens complement factor H (CFH	4.63	8			8			
B2RE24_HUMAN	B2RE24_HUMAN	B2RE24	cDNA, FLJ96882, highly similar to Homo sapiens carbonic anhydrase XII	14.1	8			8			

B2RAY1_HUMAN	B2RAY1_HUMAN	B2RAY1	cDNA, FLJ95184, highly similar to Homo sapiens signal transducing adap	6.67	8	5	1	2			
B4E3I3_HUMAN	B4E3I3_HUMAN	B4E3I3	cDNA FLJ59614, highly similar to Ubiquitin fusion degradation protein	7.94	8	7		1			
Q5JB40_HUMAN	Q5JB40_HUMAN	Q5JB40	Selectin-like protein	1.68	8	7		1			
B4DTZ8_HUMAN	B4DTZ8_HUMAN	B4DTZ8	cDNA FLJ52277, highly similar to Follistatin-related protein 1	9.56	8	5	2				
Q53FU3_HUMAN	Q53FU3_HUMAN	Q53FU3	Coatomer protein complex, subunit zeta 1 variant (Fragment)	25.4	8	7	1				
D6W4Z6_HUMAN	hCG_23833	D6W4Z6	HCG23833, isoform CRA_b	10.2	8	8					
D6RGV2_HUMAN	IL7R	D6RGV2	Interleukin-7 receptor subunit alpha	11.5	8	7					
A8K245_HUMAN	A8K245_HUMAN	A8K245	cDNA FLJ75441, highly similar to Homo sapiens vaccinia related kinase	13.6	7				8		
B2R9K6_HUMAN	B2R9K6_HUMAN	B2R9K6	cDNA, FLJ94436, highly similar to Homo sapiens platelet derived growth	11.6	7				7		
Q53GD7_HUMAN	Q53GD7_HUMAN	Q53GD7	FUS interacting protein (Serine-arginine rich) 1 isoform 2 variant (Fr	19.1	7				7		
B2RDN3_HUMAN	B2RDN3_HUMAN	B2RDN3	cDNA, FLJ96692, highly similar to Homo sapiens nucleotide binding prot	8.49	7			2	5		
A0JLQ5_HUMAN	BXDC2	A0JLQ5	BXDC2 protein (Fragment)	8.06	7	3			5		
B2RBP3_HUMAN	B2RBP3_HUMAN	B2RBP3	cDNA, FLJ95615, highly similar to Homo sapiens ubiquitin-activating en	4.54	7	3			4		
B2R6H7_HUMAN	B2R6H7_HUMAN	B2R6H7	cDNA, FLJ92955, highly similar to Homo sapiens transportin-SR (TRN-SR)	3.47	7	2	1	1	3		
A8K5L5_HUMAN	A8K5L5_HUMAN	A8K5L5	cDNA FLJ75689, highly similar to Homo sapiens poliovirus receptor-rela	8.35	7	1	3		3		

B4DKC1_HUMAN	B4DKC1_HUMAN	B4DKC1	cDNA FLJ53349, highly similar to Transmembrane 9 superfamily protein m	4.66	7	4			3		
B4DVP2_HUMAN	B4DVP2_HUMAN	B4DVP2	cDNA FLJ57208, highly similar to 5'-AMP-activated protein kinase subun	11.2	7	4			3		
B2RB07_HUMAN	B2RB07_HUMAN	B2RB07	cDNA, FLJ95243, highly similar to Homo sapiens ubiquitin carboxyl-term	8.51	7	5			2		
B4E1E2_HUMAN	B4E1E2_HUMAN	B4E1E2	cDNA FLJ61530, highly similar to Hepatocyte growth factor-regulated ty	3.33	7	2	2	2	1		
B3KM36_HUMAN	B3KM36_HUMAN	B3KM36	cDNA FLJ10153 fis, clone HEMBA1003417, highly similar to BAG family mo	15.6	7	2	3	1	1		
Q59FB9_HUMAN	Q59FB9_HUMAN	Q59FB9	Toll interacting protein variant (Fragment)	7.67	7	4	2	1	1		
Q59FC3_HUMAN	Q59FC3_HUMAN	Q59FC3	G protein-coupled receptor kinase interactor 1 variant (Fragment)	3.88	7	6		1	1		
B4DKQ5_HUMAN	B4DKQ5_HUMAN	B4DKQ5	cDNA FLJ54710, highly similar to Target of Myb protein 1	5.79	7	5	1		1		
B4DG62_HUMAN	B4DG62_HUMAN	B4DG62	cDNA FLJ56506, highly similar to Hexokinase-1 (EC 2.7.1.1)	4.7	7	4	1		1		
T3HPD_HUMAN	L3HYPDH	Q96EM0	Trans-L-3-hydroxyproline dehydratase (EC 4.2.1.77) (Trans-3-hydroxy-l-	8.47	7	6			1		
A8K9W7_HUMAN	A8K9W7_HUMAN	A8K9W7	cDNA FLJ77440, highly similar to Homo sapiens general transcription fa	5.21	7			7			
Q96K68_HUMAN	Q96K68_HUMAN	Q96K68	cDNA FLJ14473 fis, clone MAMMA1001080, highly similar to Homo sapiens	8.3	7			7			
B4DXF3_HUMAN	B4DXF3_HUMAN	B4DXF3	cDNA FLJ57650, highly similar to Bleomycin hydrolase (EC 3.4.22.40)	6.25	7	2		4			
B4DVT6_HUMAN	B4DVT6_HUMAN	B4DVT6	cDNA FLJ52170, highly similar to Homo sapiens pyrroline-5-carboxylate	14.2	7	5		2			

B2R7T2_HUMAN	B2R7T2_HUMAN	B2R7T2	cDNA, FLJ93591, highly similar to Homo sapiens transforming growth fac	8.94	7	6		1			
B7Z6S9_HUMAN	B7Z6S9_HUMAN	B7Z6S9	cDNA FLJ56157, highly similar to Glucosylceramidase (EC 3.2.1.45)	5.96	7	3	4				
Q9UHS8_HUMAN	Q9UHS8_HUMAN	Q9UHS8	PRO1975	9.16	7	4	3				
B4E0Z6_HUMAN	B4E0Z6_HUMAN	B4E0Z6	cDNA FLJ59809, highly similar to Bone marrow stromal antigen 2	6.94	7	6	1				
B2R6N9_HUMAN	B2R6N9_HUMAN	B2R6N9	cDNA, FLJ93042, highly similar to Homo sapiens signal sequence recepto	11.9	7	6	1				
5NT3A_HUMAN	NT5C3A	Q9H0P0	Cytosolic 5'-nucleotidase 3A (EC 3.1.3.5) (Cytosolic 5'-nucleotidase 3	13.1	7	8					
B4DP80_HUMAN	B4DP80_HUMAN	B4DP80	cDNA FLJ56357, highly similar to Homo sapiens apolipoprotein A-I bindi	16.9	7	7					
B3KX16_HUMAN	B3KX16_HUMAN	B3KX16	cDNA FLJ44469 fis, clone UTERU2026090, highly similar to Cartilage-ass	11.3	7	7					
A8KAM8_HUMAN	A8KAM8_HUMAN	A8KAM8	Tyrosine-protein kinase receptor (EC 2.7.10.1)	3.98	7	7					
B4DRB1_HUMAN	B4DRB1_HUMAN	B4DRB1	cDNA FLJ50735, highly similar to Calsyntenin-3	4.71	7	7					
A8K946_HUMAN	A8K946_HUMAN	A8K946	mRNA cap guanine-N7 methyltransferase (EC 2.1.1.56)	7.74	6				6		
B2RB06_HUMAN	B2RB06_HUMAN	B2RB06	cDNA, FLJ95242, highly similar to Homo sapiens L-3-hydroxyacyl-Coenzym	11.1	6			1	5		
Q53EQ3_HUMAN	Q53EQ3_HUMAN	Q53EQ3	Tubulin, gamma complex associated protein 2 variant (Fragment)	9.77	6	1			5		
B7Z4Z2_HUMAN	B7Z4Z2_HUMAN	B7Z4Z2	cDNA FLJ52440, highly similar to Lysosomal acid phosphatase (EC 3.1.3.	8.06	6		2		4		

Q59GY3_HUMAN	Q59GY3_HUMAN	Q59GY3	Arginine/serine-rich splicing factor 6 variant (Fragment)	13.3	6	1	1		4		
B7Z561_HUMAN	B7Z561_HUMAN	B7Z561	cDNA FLJ53154, highly similar to Neutral amino acid transporter A	5.31	6	1	3	2	3		
B7Z920_HUMAN	B7Z920_HUMAN	B7Z920	cDNA FLJ61714, highly similar to Tripeptidyl-peptidase 2 (EC 3.4.14.10	2.53	6		1	2	3		
A8K885_HUMAN	A8K885_HUMAN	A8K885	cDNA FLJ77179, highly similar to Homo sapiens sorting nexin 6 (SNX6) m	6.4	6	2		1	3		
Q59EC0_HUMAN	Q59EC0_HUMAN	Q59EC0	Adenosine deaminase, RNA-specific isoform ADAR-a variant (Fragment)	5.06	6	3			3		
A8K923_HUMAN	A8K923_HUMAN	A8K923	cDNA FLJ78690, highly similar to Homo sapiens protein phosphatase 4, r	2.36	6	1	1	2	2		
Q69YP2_HUMAN	DKFZp762K123	Q69YP2	Putative uncharacterized protein DKFZp762K123 (Fragment)	5.65	6	4			2		
B4DND6_HUMAN	B4DND6_HUMAN	B4DND6	cDNA FLJ55804, highly similar to COP9 signalosome complex subunit 1	3.73	6	2		3	1		
A4D0R2_HUMAN	SYPL	A4D0R2	Synaptophysin-like 1, isoform CRA_b (Synaptophysin-like protein)	4.56	6	2	3	2	1		
Q9NXW1_HUMAN	Q9NXW1_HUMAN	Q9NXW1	BJ-HCC-24 tumor antigen (CDNA FLJ20030 fis, clone ADSU02156)	5.83	6	2		2	1		
B4DXC4_HUMAN	B4DXC4_HUMAN	B4DXC4	cDNA FLJ58636, moderately similar to Atlastin	3.63	6	2	3		1		
B4E0U7_HUMAN	B4E0U7_HUMAN	B4E0U7	cDNA FLJ54264, highly similar to Exocyst complex component 5	3.73	6		2	4			
A8K8N5_HUMAN	A8K8N5_HUMAN	A8K8N5	Ubiquitin carboxyl-terminal hydrolase (EC 3.4.19.12)	2.77	6		2	3			
B3KQF4_HUMAN	B3KQF4_HUMAN	B3KQF4	cDNA FLJ90373 fis, clone NT2RP2004606, highly similar to Metalloprotei	18.8	6	3		3			

A8K6L3_HUMAN	A8K6L3_HUMAN	A8K6L3	cDNA FLJ76883, highly similar to Homo sapiens FKSG44 gene (FKSG44), mR	12.7	6	5	1				
A8K2H4_HUMAN	A8K2H4_HUMAN	A8K2H4	cDNA FLJ78235	15	6	4	1				
B4DJW1_HUMAN	B4DJW1_HUMAN	B4DJW1	cDNA FLJ54329, highly similar to Aconitate hydratase, mitochondrial(EC	2.58	6	6					
Q59GW7_HUMAN	Q59GW7_HUMAN	Q59GW7	Replication factor C 5 isoform 1 variant (Fragment)	10.3	5				5		
A8K670_HUMAN	A8K670_HUMAN	A8K670	cDNA FLJ75703, highly similar to Homo sapiens nitric oxide synthase in	9.97	5				5		
NAKD2_HUMAN	NADK2	Q4G0N4	NAD kinase 2, mitochondrial (EC 2.7.1.23) (Mitochondrial NAD kinase) (	7.01	5				5		
B3KN45_HUMAN	B3KN45_HUMAN	B3KN45	cDNA FLJ13521 fis, clone PLACE1005876, highly similar to Cleavage and	3.91	5				5		
Q52NV4_HUMAN	HRS	Q52NV4	Histidyl-tRNA synthetase	6.48	5			1	4		
A8K905_HUMAN	A8K905_HUMAN	A8K905	cDNA FLJ77615, highly similar to Homo sapiens nucleolar complex associ	3.5	5	1			4		
B7Z8G2_HUMAN	B7Z8G2_HUMAN	B7Z8G2	cDNA FLJ58213, highly similar to FK506-binding protein 5 (EC 5.2.1.8)	6.9	5	1			4		
B4DU42_HUMAN	B4DU42_HUMAN	B4DU42	cDNA FLJ56153, highly similar to Homo sapiens transforming growth fact	3.74	5			2	3		
B7Z323_HUMAN	B7Z323_HUMAN	B7Z323	cDNA FLJ52497, highly similar to Protein NDRG3	5.3	5	2			3		
B4DLT2_HUMAN	B4DLT2_HUMAN	B4DLT2	cDNA FLJ56637, highly similar to Nuclear pore complex protein Nup155	2.14	5	2			3		
A9UK01_HUMAN	A9UK01_HUMAN	A9UK01	Rho GTPase activating protein	6.49	5	4			2		

B2RAU5_HUMAN	B2RAU5_HUMAN	B2RAU5	Sorting nexin	2.35	5	3			2		
A8K2Z9_HUMAN	A8K2Z9_HUMAN	A8K2Z9	Receptor-type tyrosine-protein phosphatase (EC 3.1.3.48)	3.89	5	3			2		
B3KML1_HUMAN	B3KML1_HUMAN	B3KML1	cDNA FLJ11308 fis, clone PLACE1010074, highly similar to Sorting nexin	7.51	5	3		1	1		
Q59EZ3_HUMAN	Q59EZ3_HUMAN	Q59EZ3	Insulin-like growth factor 2 receptor variant (Fragment)	1.28	5	4			1		
A6XNE2_HUMAN	A6XNE2_HUMAN	A6XNE2	Complement factor D preproprotein	12.3	5			5			
Q6N094_HUMAN	DKFZp686O01196	Q6N094	Putative uncharacterized protein DKFZp686O01196	8.12	5	2		4			
B7Z1V1_HUMAN	B7Z1V1_HUMAN	B7Z1V1	cDNA FLJ50123	6.86	5	1		4			
B2R7M1_HUMAN	B2R7M1_HUMAN	B2R7M1	cDNA, FLJ93507, highly similar to Homo sapiens ATPase, H+ transporting	14	5	3	3				
B2R6A3_HUMAN	B2R6A3_HUMAN	B2R6A3	cDNA, FLJ92860, highly similar to Homo sapiens solute carrier family 9	7.82	5	3	3				
A6QKW0_HUMAN	SHINC3	A6QKW0	SHINC3	4.86	5	2	3				
A8K249_HUMAN	A8K249_HUMAN	A8K249	Caspase	12.6	5	5					
Q9UG64_HUMAN	DKFZp586I1223	Q9UG64	Putative uncharacterized protein DKFZp586I1223 (Fragment)	12.7	4				4		
VPS51_HUMAN	VPS51	Q9UID3	Vacuolar protein sorting-associated protein 51 homolog (Another new ge	5.88	4				4		
A8K5L4_HUMAN	A8K5L4_HUMAN	A8K5L4	cDNA FLJ76843, highly similar to Homo sapiens guanine nucleotide bindi	6.95	4				4		

B4DSH1_HUMAN	B4DSH1_HUMAN	B4DSH1	cDNA FLJ51295, highly similar to Cell division cycle 5-like protein	3.23	4				4		
B4DG11_HUMAN	B4DG11_HUMAN	B4DG11	cDNA FLJ55382, highly similar to Hsp70-binding protein 1	6.67	4				4		
D3DRP5_HUMAN	C9orf19	D3DRP5	Chromosome 9 open reading frame 19, isoform CRA_a (Fragment)	7.41	4				4		
B2R7R5_HUMAN	B2R7R5_HUMAN	B2R7R5	cDNA, FLJ93570, highly similar to Homo sapiens phosphoribosyl pyrophos	7.59	4			1	3		
B3KTT0_HUMAN	B3KTT0_HUMAN	B3KTT0	cDNA FLJ38675 fis, clone IMR322000243, highly similar to Nucleoporin N	10.8	4			1	3		
B4DW43_HUMAN	B4DW43_HUMAN	B4DW43	cDNA FLJ56162, highly similar to N-acetylgalactosamine kinase (EC 2.7.	5.3	4	1		1	2		
Q8TF11_HUMAN	Q8TF11_HUMAN	Q8TF11	Vacuolar proton pump subunit SFD alpha isoform	6.51	4	2			2		
B4DKS0_HUMAN	B4DKS0_HUMAN	B4DKS0	cDNA FLJ53381, highly similar to Monocarboxylate transporter 1	6.25	4	4			1		
A8KA83_HUMAN	A8KA83_HUMAN	A8KA83	cDNA FLJ78586, highly similar to Homo sapiens VAMP (vesicle-associated	4.96	4	3			1		
A8K5A4_HUMAN	A8K5A4_HUMAN	A8K5A4	cDNA FLJ76826, highly similar to Homo sapiens ceruloplasmin (ferroxida	3.94	4			4			
A8K417_HUMAN	A8K417_HUMAN	A8K417	cDNA FLJ78087, highly similar to Homo sapiens homogentisate 1,2-dioxyg	4.72	4			4			
B2R8N1_HUMAN	B2R8N1_HUMAN	B2R8N1	cDNA, FLJ93976, highly similar to Homo sapiens COP9 homolog (COP9), mR	13.4	4	2		2			
B4DWB1_HUMAN	B4DWB1_HUMAN	B4DWB1	Protein arginine N-methyltransferase	2.71	4	2		2			
Q5HYL6_HUMAN	DKFZp686E1899	Q5HYL6	Putative uncharacterized protein DKFZp686E1899	3.98	4	3		1			

B7Z965_HUMAN	B7Z965_HUMAN	B7Z965	cDNA FLJ56337, highly similar to High mobility group protein B1	8.62	4	1	4				
B7Z2G0_HUMAN	B7Z2G0_HUMAN	B7Z2G0	cDNA FLJ54266, highly similar to G-protein coupled receptor family C g	4.56	4		4				
APMAP_HUMAN	APMAP	Q9HDC9	Adipocyte plasma membrane-associated protein (Protein BSCv)	2.88	4	1	3				
A8K4S9_HUMAN	A8K4S9_HUMAN	A8K4S9	Signal transducer and activator of transcription	3.54	4	6					
Q658S9_HUMAN	DKFZp666I134	Q658S9	Putative uncharacterized protein DKFZp666I134 (Fragment)	8.67	4	4					
B4DWL1_HUMAN	B4DWL1_HUMAN	B4DWL1	cDNA FLJ59240, highly similar to Far upstream element-binding protein	3.43	4	4					
A8K6H1_HUMAN	A8K6H1_HUMAN	A8K6H1	cDNA FLJ76662, highly similar to Homo sapiens type 1 tumor necrosis fa	4.04	4	4					
B4DRC7_HUMAN	B4DRC7_HUMAN	B4DRC7	cDNA FLJ57829	3.4	4	4					
B4DTX0_HUMAN	B4DTX0_HUMAN	B4DTX0	cDNA FLJ60891, highly similar to Macrophage colony-stimulating factor	6.49	4	4					
Q59FU8_HUMAN	Q59FU8_HUMAN	Q59FU8	Tumor necrosis factor receptor superfamily, member 6 isoform 1 variant	4.14	4	4					
E5KN59_HUMAN	E5KN59_HUMAN	E5KN59	Peptidyl-prolyl cis-trans isomerase D	4.59	3				4		
Q53GS0_HUMAN	Q53GS0_HUMAN	Q53GS0	Nucleolar GTP-binding protein 1 (Fragment)	2.05	3				3		
NELFB_HUMAN	NELFB	Q8WX92	Negative elongation factor B (NELF-B) (Cofactor of BRCA1)	4.31	3				3		
B3KNC3_HUMAN	B3KNC3_HUMAN	B3KNC3	cDNA FLJ14222 fis, clone NT2RP3003992, highly similar to Nucleolar com	2.94	3				3		

B3KP06_HUMAN	B3KP06_HUMAN	B3KP06	cDNA FLJ30880 fis, clone FEBRA2004767, highly similar to Breast cancer	1.21	3				3		
B2RAR3_HUMAN	B2RAR3_HUMAN	B2RAR3	Queuine tRNA-ribosyltransferase (EC 2.4.2.29)	3.47	3				3		
B2RCP7_HUMAN	B2RCP7_HUMAN	B2RCP7	cDNA, FLJ96197, highly similar to Homo sapiens connective tissue growt	6.3	3				3		
A8K9B2_HUMAN	A8K9B2_HUMAN	A8K9B2	cDNA FLJ76725, highly similar to Homo sapiens L-2-hydroxyglutarate deh	4.97	3				3		
B4DE94_HUMAN	B4DE94_HUMAN	B4DE94	cDNA FLJ57707, highly similar to Ketosamine-3-kinase (EC 2.7.1.-)	11	3				3		
B4DH44_HUMAN	B4DH44_HUMAN	B4DH44	cDNA FLJ52538, highly similar to Dual specificity mitogen-activated pr	7.98	3				3		
B2RC50_HUMAN	B2RC50_HUMAN	B2RC50	cDNA, FLJ95853, highly similar to Homo sapiens exosome component Rrp46	9.36	3				3		
B4DYF4_HUMAN	B4DYF4_HUMAN	B4DYF4	cDNA FLJ56176, highly similar to Poly(A) polymerase alpha (EC 2.7.7.19	3.15	3			1	2		
B7ZL68_HUMAN	NRP2	B7ZL68	Neuropilin 2	3.35	3			1	2		
B2R6E2_HUMAN	B2R6E2_HUMAN	B2R6E2	cDNA, FLJ92910, highly similar to Homo sapiens programmed cell death 4	4.69	3		1		2		
B4E324_HUMAN	B4E324_HUMAN	B4E324	cDNA FLJ60397, highly similar to Lysosomal protective protein (EC 3.4.	5.62	3		1		2		
B3KM47_HUMAN	B3KM47_HUMAN	B3KM47	cDNA FLJ10273 fis, clone HEMBB1001137, highly similar to SEC23-interac	3.9	3	2			2		
D4YW74_HUMAN	TROPH	D4YW74	Polytrophin	0.19	3			2	1		
B4DZ28_HUMAN	B4DZ28_HUMAN	B4DZ28	cDNA FLJ53577, highly similar to pre-mRNA-splicing factor ATP-dependen	2.14	3			2	1		
B7Z4J7_HUMAN	B7Z4J7_HUMAN	B7Z4J7	Gephyrin	3.69	3			2	1		

A8K9T8_HUMAN	A8K9T8_HUMAN	A8K9T8	cDNA FLJ76106, highly similar to Homo sapiens neurolysin (metallopepti	3.55	3			2	1		
H0Y858_HUMAN	H0Y858_HUMAN	H0Y858	Uncharacterized protein (Fragment)	2.02	3	1	1		1		
Q59G24_HUMAN	Q59G24_HUMAN	Q59G24	Activated RNA polymerase II transcription cofactor 4 variant (Fragment	17.9	3	3			1		
B2R694_HUMAN	B2R694_HUMAN	B2R694	cDNA, FLJ92849, highly similar to Homo sapiens lanosterol synthase (2,	2.05	3			3			
A8K651_HUMAN	A8K651_HUMAN	A8K651	cDNA FLJ75700, highly similar to Homo sapiens complement component 1,	10.3	3			3			
B2R758_HUMAN	B2R758_HUMAN	B2R758	cDNA, FLJ93295, highly similar to Homo sapiens glucosaminyl (N-acetyl)	4.11	3			3			
E5KMT6_HUMAN	E5KMT6_HUMAN	E5KMT6	tRNA pseudouridine synthase (EC 5.4.99.-)	2.51	3	1		2			
Q7Z426_HUMAN	Q7Z426_HUMAN	Q7Z426	Putative MAPK activating protein	4.55	3	2		1			
B4DQY2_HUMAN	B4DQY2_HUMAN	B4DQY2	cDNA FLJ59388, highly similar to Mitochondrial inner membrane protein	2.67	3		3				
A8K2H6_HUMAN	A8K2H6_HUMAN	A8K2H6	Lipase	6.77	3	1	2				
Q53HC3_HUMAN	Q53HC3_HUMAN	Q53HC3	Solute carrier family 25 member 3 isoform b variant (Fragment)	3.32	3	2	1				
B2RAH2_HUMAN	B2RAH2_HUMAN	B2RAH2	Sodium/hydrogen exchanger	1.72	3	3					
Q59E97_HUMAN	Q59E97_HUMAN	Q59E97	Galectin (Fragment)	7.69	3	3					
B3KQ18_HUMAN	B3KQ18_HUMAN	B3KQ18	cDNA FLJ32632 fis, clone SYNOV1000190, highly similar to Alpha-1,3-man	6.74	3	3					

Q59GD7_HUMAN	Q59GD7_HUMAN	Q59GD7	Transporter (Fragment)	5.72	3	3					
A8K6F0_HUMAN	A8K6F0_HUMAN	A8K6F0	cDNA FLJ75449, highly similar to Homo sapiens aquarius homolog (mouse)	2.09	2				3		
B4DDK3_HUMAN	B4DDK3_HUMAN	B4DDK3	cDNA FLJ53706, highly similar to Cell cycle control protein 50A	5.78	2				3		
B4DMA4_HUMAN	B4DMA4_HUMAN	B4DMA4	cDNA FLJ58400, highly similar to Cleavage stimulation factor 77 kDa su	4.77	2				2		
B4DWZ7_HUMAN	B4DWZ7_HUMAN	B4DWZ7	cDNA FLJ56802, highly similar to LanC-like protein 2	3.01	2				2		
B7Z592_HUMAN	B7Z592_HUMAN	B7Z592	cDNA FLJ61635, highly similar to Homo sapiens likely ortholog of mouse	2.55	2				2		
A8K800_HUMAN	A8K800_HUMAN	A8K800	cDNA FLJ76924, highly similar to Homo sapiens brix domain containing 1	2.61	2				2		
B4DQ62_HUMAN	B4DQ62_HUMAN	B4DQ62	cDNA FLJ51935, highly similar to Butyrophilin subfamily 3 member A1	5.31	2				2		
A8K5S1_HUMAN	A8K5S1_HUMAN	A8K5S1	cDNA FLJ78650, highly similar to Homo sapiens mucosa associated lympho	2.18	2				2		
B2RDN4_HUMAN	B2RDN4_HUMAN	B2RDN4	cDNA, FLJ96693, highly similar to Homo sapiens block of proliferation	2.14	2				2		
B3KX30_HUMAN	B3KX30_HUMAN	B3KX30	cDNA FLJ44557 fis, clone UTERU3007913, highly similar to FERM domain-c	4.95	2				2		
B7Z8T5_HUMAN	B7Z8T5_HUMAN	B7Z8T5	cDNA FLJ53003, highly similar to Presenilin-1 (EC 3.4.23.-)	5.68	2				2		
B4DST3_HUMAN	B4DST3_HUMAN	B4DST3	cDNA FLJ56487, highly similar to Methionine synthase (EC 2.1.1.13)	2.33	2				2		
B7Z879_HUMAN	B7Z879_HUMAN	B7Z879	cDNA FLJ54956, highly similar to Vacuolar protein sorting 11	1.18	2				2		

F5H2D1_HUMAN	TENM2	F5H2D1	Teneurin-2	0.865	2				2		
B3KRR1_HUMAN	B3KRR1_HUMAN	B3KRR1	cDNA FLJ34725 fis, clone MESAN2005958, highly similar to RNA-binding p	7.08	2				2		
B7ZB52_HUMAN	B7ZB52_HUMAN	B7ZB52	cDNA, FLJ79416, highly similar to Adapter-relatedprotein complex 4 sig	11.1	2				2		
B2RCZ4_HUMAN	B2RCZ4_HUMAN	B2RCZ4	cDNA, FLJ96384, highly similar to Homo sapiens protein kinase C, iota	5.11	2	1		1	1		
A8K6D2_HUMAN	A8K6D2_HUMAN	A8K6D2	cDNA FLJ76620, highly similar to Homo sapiens C2f protein (C2F), mRNA	4.51	2			1	1		
MK67I_HUMAN	NIFK	Q9BYG3	MKI67 FHA domain-interacting nucleolar phosphoprotein (Nucleolar phosp	4.78	2	1			1		
B2R8A2_HUMAN	B2R8A2_HUMAN	B2R8A2	cDNA, FLJ93804, highly similar to Homo sapiens gp25L2 protein (HSGP25L	4.67	2	1			1		
B4DSZ1_HUMAN	B4DSZ1_HUMAN	B4DSZ1	cDNA FLJ54877, highly similar to Syntaxin-12	13.5	2	1			1		
A8K9G6_HUMAN	A8K9G6_HUMAN	A8K9G6	cDNA FLJ75133, highly similar to Homo sapiens NACHT, leucine rich repe	2.93	2			2			
CCD68_HUMAN	CCDC68	Q9H2F9	Coiled-coil domain-containing protein 68 (Cutaneous T-cell lymphoma-as	7.76	2	1	1				
B2R665_HUMAN	B2R665_HUMAN	B2R665	cDNA, FLJ92810, highly similar to Homo sapiens protein phosphatase 1G	5.13	2	2					
HELZ2_HUMAN	HELZ2	Q9BYK8	Helicase with zinc finger domain 2 (ATP-dependent helicase PRIC285) (H	1.13	2	2					
A8K1C7_HUMAN	A8K1C7_HUMAN	A8K1C7	cDNA FLJ75179, highly similar to Homo sapiens leucine rich repeat cont	1.6	2	2					
B4DVT8_HUMAN	B4DVT8_HUMAN	B4DVT8	cDNA FLJ60173, highly similar to Zinc-finger protein ZPR1	3.43	2	2					

B2RC06_HUMAN	B2RC06_HUMAN	B2RC06	cDNA, FLJ95791, highly similar to Homo sapiens aurora kinase B (AURKB)	4.36	2	2					
A8K6Q9_HUMAN	A8K6Q9_HUMAN	A8K6Q9	cDNA FLJ75882, highly similar to Homo sapiens spastic paraplegia 20, s	2.25	2	2					
B4DL07_HUMAN	B4DL07_HUMAN	B4DL07	cDNA FLJ53353, highly similar to ATP-binding cassette sub-family D mem	1.76	2	2					
A8K2D6_HUMAN	A8K2D6_HUMAN	A8K2D6	cDNA FLJ76011, highly similar to Homo sapiens lactamase, beta 2 (LACTB	3.12	2	2					
A8K8X0_HUMAN	A8K8X0_HUMAN	A8K8X0	cDNA FLJ75187, highly similar to Homo sapiens nap1 P120	2.37	2	2					
B3KW79_HUMAN	B3KW79_HUMAN	B3KW79	cDNA FLJ42481 fis, clone BRACE2032090, highly similar to Probable seri	6.93	2	2					
B2RA14_HUMAN	B2RA14_HUMAN	B2RA14	cDNA, FLJ94653, highly similar to Homo sapiens tumor necrosis factor (	5.12	3				3		
B4DHK5_HUMAN	B4DHK5_HUMAN	B4DHK5	cDNA FLJ61590, moderately similar to Pleckstrin homology domain-contai	0.845	2				2		
A8K940_HUMAN	A8K940_HUMAN	A8K940	cDNA FLJ77630, highly similar to Homo sapiens BPY2 interacting protein	1.23	2				2		
Q53G69_HUMAN	Q53G69_HUMAN	Q53G69	Translocase of inner mitochondrial membrane 44 homolog (Fragment)	2.65	1				1		
B4DEA6_HUMAN	B4DEA6_HUMAN	B4DEA6	cDNA FLJ56566, highly similar to Small glutamine-rich tetratricopeptid	4.81	1				1		
B2RDV7_HUMAN	B2RDV7_HUMAN	B2RDV7	cDNA, FLJ96789, highly similar to Homo sapiens dihydrouridine synthase	2	1				1		
A8K874_HUMAN	A8K874_HUMAN	A8K874	cDNA FLJ77588, highly similar to Homo sapiens MAK10 homolog, amino-aci	1.1	1				1		
B2RD46_HUMAN	B2RD46_HUMAN	B2RD46	cDNA, FLJ96449	1.98	1				1		

B4DNG0_HUMAN	B4DNG0_HUMAN	B4DNG0	cDNA FLJ58142, highly similar to Olfactomedin-like protein 3	3.48	1				1		
Q59HG1_HUMAN	Q59HG1_HUMAN	Q59HG1	Chromosome-associated kinesin KIF4A variant (Fragment)	0.729	1				1		
B4E1D9_HUMAN	B4E1D9_HUMAN	B4E1D9	cDNA FLJ55318, highly similar to Casein kinase I isoform alpha (EC 2.7	5.7	1				1		
B4E256_HUMAN	B4E256_HUMAN	B4E256	cDNA FLJ55348, highly similar to WD repeat protein 6	1.31	1				1		
A2A2Q9_HUMAN	AAR2	A2A2Q9	Protein AAR2 homolog	4.02	1				1		
B2RCM6_HUMAN	B2RCM6_HUMAN	B2RCM6	cDNA, FLJ96161	3.56	1				1		
A8K503_HUMAN	A8K503_HUMAN	A8K503	cDNA FLJ76182, highly similar to Homo sapiens ubiquitin-conjugating en	5.06	1				1		
Q8WWH9_HUMAN	Q8WWH9_HUMAN	Q8WWH9	Amphiphysin IIb-1	1.43	1				1		
B7Z4W4_HUMAN	B7Z4W4_HUMAN	B7Z4W4	cDNA FLJ50817, highly similar to UV excision repair protein RAD23 homo	2.06	1				1		
B4DK32_HUMAN	B4DK32_HUMAN	B4DK32	cDNA FLJ61095, highly similar to Ubiquitin conjugation factor E4 A (Fr	1.15	1				1		
Q63HL4_HUMAN	DKFZp686G04235	Q63HL4	NADPH--cytochrome P450 reductase (EC 1.6.2.4)	1.32	1			1			
T132B_HUMAN	TMEM132B	Q14DG7	Transmembrane protein 132B	0.928	1			1			
A8K6V6_HUMAN	A8K6V6_HUMAN	A8K6V6	cDNA FLJ75883, highly similar to Homo sapiens glucosamine (N-acetyl)-6	2.17	1		1				
B3KPC7_HUMAN	B3KPC7_HUMAN	B3KPC7	Actin-related protein 2/3 complex subunit 5	8.5	1		1				
B2R838_HUMAN	B2R838_HUMAN	B2R838	cDNA, FLJ93726, highly similar to Homo sapiens ras homolog gene family	5.33	1		1				

A8K9U4_HUMAN	A8K9U4_HUMAN	A8K9U4	cDNA FLJ77205, highly similar to Homo sapiens hect domain and RLD 4 (H	1.17	1		1				
B3KY30_HUMAN	B3KY30_HUMAN	B3KY30	cDNA FLJ46699 fis, clone TRACH3013700, highly similar to Stress 70 pro	4.56	2	2					
B4DNZ1_HUMAN	B4DNZ1_HUMAN	B4DNZ1	cDNA FLJ53633, highly similar to Transmembrane BAX inhibitor motif-con	5.62	2	2					
B3KNI2_HUMAN	B3KNI2_HUMAN	B3KNI2	cDNA FLJ14650 fis, clone NT2RP2002185, highly similar to Ubiquilin-1	2.72	2	2					
B3KM97_HUMAN	B3KM97_HUMAN	B3KM97	cDNA FLJ10554 fis, clone NT2RP2002385, highly similar to Synaptic glyc	3.9	1	1					
A8K2Q6_HUMAN	A8K2Q6_HUMAN	A8K2Q6	Peptidyl-prolyl cis-trans isomerase (EC 5.2.1.8)	6.13	1	1					
A8K7T4_HUMAN	A8K7T4_HUMAN	A8K7T4	cDNA FLJ75774, highly similar to Homo sapiens lectin, mannose-binding	3.65	1	1					
B2R8K8_HUMAN	B2R8K8_HUMAN	B2R8K8	cDNA, FLJ93949, highly similar to Homo sapiens NIMA (never in mitosis	2.65	1	1					
B7Z3E3_HUMAN	B7Z3E3_HUMAN	B7Z3E3	Reticulon	1.41	1	1					
B3KSG7_HUMAN	B3KSG7_HUMAN	B3KSG7	cDNA FLJ36171 fis, clone TESTI2026215, highly similar to Homo sapiens	1.47	1	1					
E7ENX8_HUMAN	E7ENX8_HUMAN	E7ENX8	Uncharacterized protein (Fragment)	2.02	1	1					
B7Z6K2_HUMAN	B7Z6K2_HUMAN	B7Z6K2	cDNA FLJ58625, highly similar to Polypeptide N-acetylgalactosaminyltra	3.19	1	1					
M1VE83_HUMAN	SDC4-ROS1_S4;R34	M1VE83	Tyrosine-protein kinase receptor (EC 2.7.10.1)	1.56	1	1					
B7ZW70_HUMAN	LOC727761	B7ZW70	LOC727761 protein	5.85	1	1					

B7Z8X5_HUMAN	B7Z8X5_HUMAN	B7Z8X5	cDNA FLJ61541, highly similar to Homo sapiens PDZ and LIM domain 5 (PD	2.07	1	1					
A8K3J8_HUMAN	A8K3J8_HUMAN	A8K3J8	cDNA FLJ75332, highly similar to Homo sapiens Ras-related associated w	3.57	1	1					

Table S5. Immune-associated proteins in mesothelioma-derived exosomes.

	Gene Name	Protein Accession	Protein Description	SpC Combined (4 cell lines)	Vesiclepedia comparison (BC, DC, TC)	Mesothelioma exosome cancer signature (mEXOS)
Antigen processing and presentation (general)	TNC	Q4LE33	TNC variant protein (Fragment)	16		
	TGFBI	Q15582	Transforming growth factor-beta-induced protein ig-h3 (Beta ig-h3) (Ke	112		
	CYFIP1	Q7L576	Cytoplasmic FMR1-interacting protein 1 (Specifically Rac1-associated p	115	B cells	
	EPB41L2	O43491	Band 4.1-like protein 2 (Generally expressed protein 4.1) (4.1G)	76	B cells	
	SPTBN1	Q01082	Spectrin beta chain, non-erythrocytic 1 (Beta-II spectrin) (Fodrin bet	105	B cells	
	A8K766_HUMAN	A8K766	cDNA FLJ77343, highly similar to Homo sapiens electron-transfer-flavop	28		Y
	B7Z6S9_HUMAN	B7Z6S9	cDNA FLJ56157, highly similar to Glucosylceramidase (EC 3.2.1.45)	7		Y
	AASDHPPT	Q9NRN7	L-aminoadipate-semialdehyde dehydrogenase-phosphopantetheinyl transfer	6		
	OSMR	Q99650	Oncostatin-M-specific receptor subunit beta (Interleukin-31 receptor s	2		Y
	STRN	O43815	Striatin	2		
	Q9NXW1_HUMAN	Q9NXW1	BJ-HCC-24 tumor antigen (CDNA FLJ20030 fis, clone ADSU02156)	6		Y
Antigen processing and presentation (MHC I)	CCT2	P78371	T-complex protein 1 subunit beta (TCP-1-beta) (CCT-beta)	126	B cells	
	PLS3	P13797	Plastin-3 (T-plastin)	125		
	Q53HU0_HUMAN	Q53HU0	Chaperonin containing TCP1, subunit 8 (Theta) variant (Fragment)	103		Y
	A8K590_HUMAN	A8K590	cDNA FLJ77456, highly similar to Homo sapiens interleukin enhancer bin	40		Y
	FAM120A	Q9NZB2	Constitutive coactivator of PPAR-gamma-like protein 1 (Oxidative stres	20		
	CTNND1	C9JZR2	Catenin delta-1	88		
	ACO1	P21399	Cytoplasmic aconitate hydratase (Aconitase) (EC 4.2.1.3) (Citrate hydr	41		
	ATP2B4	P23634	Plasma membrane calcium-transporting ATPase 4 (PMCA4) (EC 3.6.3.8) (Ma	64	T cells	
	PDHB	P11177	Pyruvate dehydrogenase E1 component subunit beta, mitochondrial (PDHE1	30		
	RPL12	P30050	60S ribosomal protein L12	44	B cells	
	GALE	Q14376	UDP-glucose 4-epimerase (EC 5.1.3.2) (Galactowaldenase) (UDP-galactose	36		
	KIAA1199	Q8WUJ3	Protein KIAA1199	43		
	MICAL1	Q8TDZ2	Protein-methionine sulfoxide oxidase MICAL1	5		
	MAP4K4	G5E948	Mitogen-activated protein kinase kinase kinase kinase 4 (Mitogen-activ	37		
	Q53FT8_HUMAN	Q53FT8	Proteasome subunit beta type (EC 3.4.25.1) (Fragment)	69		Y
	DKFZp586I1223	Q9UG64	Putative uncharacterized protein DKFZp586I1223 (Fragment)	4		Y
	DIP2B	Q9P265	Disco-interacting protein 2 homolog B (DIP2 homolog B)	40	B cells	
	B4E2S3_HUMAN	B4E2S3	cDNA FLJ56561	53		Y
	DNMT1	P26358	DNA (cytosine-5)-methyltransferase 1 (Dnmt1) (EC 2.1.1.37) (CXXC-type	4		
	FAM49B	Q9NUQ9	Protein FAM49B (L1)	52	B cells	

	A8K0J3_HUMAN	A8K0J3	cDNA FLJ76732, highly similar to Homo sapiens TAO kinase 3 (TAOK3), mR	14		Y
	USP9X	Q93008	Probable ubiquitin carboxyl-terminal hydrolase FAF-X (EC 3.4.19.12) (D	26	B cells	
	AK4	P27144	Adenylate kinase 4, mitochondrial (AK 4) (EC 2.7.4.10) (EC 2.7.4.6) (A	11		
	ABCC1	P33527	Multidrug resistance-associated protein 1 (ATP-binding cassette sub-fa	90	B cells	
	HSPH1	Q92598	Heat shock protein 105 kDa (Antigen NY-CO-25) (Heat shock 110 kDa prot	38	B cells	
	KIAA1033	B7ZKT9	KIAA1033 protein	16		
	SLC7A5	Q01650	Large neutral amino acids transporter small subunit 1 (4F2 light chain	31	B cells	
	ACACA	Q13085	Acetyl-CoA carboxylase 1 (ACC1) (EC 6.4.1.2) (ACC-alpha) [Includes: Bi	44		
	PTGFRN	Q9P2B2	Prostaglandin F2 receptor negative regulator (CD9 partner 1) (CD9P-1)	54		
	ALDH1A3	P47895	Aldehyde dehydrogenase family 1 member A3 (EC 1.2.1.5) (Aldehyde dehyd	56		
Antigen processing and presentation (MHC II)	DYNC1H1	Q14204	Cytoplasmic dynein 1 heavy chain 1 (Cytoplasmic dynein heavy chain 1)	750	B cells	
	SNRNP200	O75643	U5 small nuclear ribonucleoprotein 200 kDa helicase (EC 3.6.4.13) (Act	81		
	ACLY	Q4LE36	ACLY variant protein (Fragment)	473	B cells	
	GNB2L1	P63244	Guanine nucleotide-binding protein subunit beta-2-like 1 (Cell prolife	80	B cells	
	SF3B1	O75533	Splicing factor 3B subunit 1 (Pre-mRNA-splicing factor SF3b 155 kDa su	42		
	RPL3	P39023	60S ribosomal protein L3 (HIV-1 TAR RNA-binding protein B) (TARBP-B)	26	B cells	
	A8K9V7_HUMAN	A8K9V7	cDNA FLJ77207	75		Y
	CPNE1	B0QZ18	Copine-1 (HCG38213, isoform CRA_b)	46	B cells	
	ALDH18A1	P54886	Delta-1-pyrroline-5-carboxylate synthase (P5CS) (Aldehyde dehydrogenas	31		
	RPS10	P46783	40S ribosomal protein S10	37	B cells	
	B3KTQ2_HUMAN	B3KTQ2	cDNA FLJ38589 fis, clone HCHON2010074, highly similar to LACTADHERIN	105		Y
	PLIN3	O60664	Perilipin-3 (47 kDa mannose 6-phosphate receptor-binding protein) (47	19		
	FAM120C	Q9NX05	Constitutive coactivator of PPAR-gamma-like protein 2 (Protein FAM120C	6		
	PSMD8	K7EJC1	26S proteasome non-ATPase regulatory subunit 8	9		
	RAC2	P15153	Ras-related C3 botulinum toxin substrate 2 (GX) (Small G protein) (p21	16	B cells	
	MOCOS	Q96EN8	Molybdenum cofactor sulfurase (MCS) (MOS) (MoCo sulfurase) (hMCS) (EC	2		
	STX3	F8W9Y0	Syntaxin-3	10		
	COX2	I6N6Q6	Cytochrome c oxidase subunit 2	4		Y
	QSOX2	Q6ZRP7	Sulfhydryl oxidase 2 (EC 1.8.3.2) (Neuroblastoma-derived sulfhydryl ox	4		
	TOR2A	Q5JU69	Torsin-2A (Torsin family 2 member A) (Torsin-related protein 1)	5		
	C5orf51	A6NDU8	UPF0600 protein C5orf51	4		
Immune response	EEF1A1	P68104	Elongation factor 1-alpha 1 (EF-1-alpha-1) (Elongation factor Tu) (EF-	265	B cells	
	B4E1G2_HUMAN	B4E1G2	Serine hydroxymethyltransferase (EC 2.1.2.1)	59		Y
	EIF3M	Q7L2H7	Eukaryotic translation initiation factor 3 subunit M (eIF3m) (Fetal lu	26		
	PLAU	E7ESM2	Urokinase-type plasminogen activator chain B	36		
	RPS18	P62269	40S ribosomal protein S18 (Ke-3) (Ke3)	24	T cells	

	VTN	P04004	Vitronectin (VN) (S-protein) (Serum-spreading factor) (V75) [Cleaved i	27		
	TBCE	B7Z3P1	Tubulin-specific chaperone E (cDNA FLJ59210, highly similar to Tubulin	13		
	DKFZp781L0540	Q7Z3K9	Putative uncharacterized protein DKFZp781L0540 (Fragment)	9		Y
	ISYNA1	Q9NPH2	Inositol-3-phosphate synthase 1 (IPS 1) (EC 5.5.1.4) (Myo-inositol 1-p	13		
	B7Z2A1_HUMAN	B7Z2A1	cDNA FLJ56068, highly similar to DNA damage-binding protein 1	23		Y
	SORD	Q00796	Sorbitol dehydrogenase (EC 1.1.1.14) (L-iditol 2-dehydrogenase)	14		
	RAB3GAP2	Q9H2M9	Rab3 GTPase-activating protein non-catalytic subunit (RGAP-iso) (Rab3	4		
	CMTM6	Q9NX76	CKLF-like MARVEL transmembrane domain-containing protein 6 (Chemokine-	8	T cells	
	A2M	P01023	Alpha-2-macroglobulin (Alpha-2-M) (C3 and PZP-like alpha-2-macroglobul	10	B cells	
	AKR1B1	P15121	Aldose reductase (AR) (EC 1.1.1.21) (Aldehyde reductase) (Aldo-keto re	12	B cells	
	COMMD3	Q9UBI1	COMM domain-containing protein 3 (Protein Bup) (Protein PIL)	7		
	Q59FU8_HUMAN	Q59FU8	Tumor necrosis factor receptor superfamily, member 6 isoform 1 variant	4		Y
	B2R694_HUMAN	B2R694	cDNA, FLJ92849, highly similar to Homo sapiens lanosterol synthase (2,	3		Y
	TOR1B	O14657	Torsin-1B (Torsin family 1 member B)	4		
	STX7	O15400	Syntaxin-7	3	B cells	
	IFI35	P80217	Interferon-induced 35 kDa protein (IFP 35) (Ifi-35)	8		
B cell response	ITGB1	P05556	Integrin beta-1 (Fibronectin receptor subunit beta) (Glycoprotein IIa)	149	T cells	
	VTN	P04004	Vitronectin (VN) (S-protein) (Serum-spreading factor) (V75) [Cleaved i	27		
	Q59FB9_HUMAN	Q59FB9	Toll interacting protein variant (Fragment)	7		Y
NK cell response	TUBB	P07437	Tubulin beta chain (Tubulin beta-5 chain)	637	B cells	
	ACSL4	O60488	Long-chain-fatty-acid--CoA ligase 4 (EC 6.2.1.3) (Long-chain acyl-CoA	102	B cells	
	B2RDF5_HUMAN	B2RDF5	SUMO-1 activating enzyme	26		Y
	B2R9L6_HUMAN	B2R9L6	cDNA, FLJ94450, highly similar to Homo sapiens cyclin-dependent kinase	10		Y
T cell response	A8K9A4_HUMAN	A8K9A4	cDNA FLJ75154, highly similar to Homo sapiens heterogeneous nuclear ri	72		Y
	HSD17B10	Q99714	3-hydroxyacyl-CoA dehydrogenase type-2 (EC 1.1.1.35) (17-beta-hydroxys	38		
	ESYT2	H7BXI1	Extended synaptotagmin-2 (Fragment)	4		
	TNS3	E7ERH3	Tensin-3	11		
	LARP1	Q6PKG0	La-related protein 1 (La ribonucleoprotein domain family member 1)	7		
	GNE	Q9Y223	Bifunctional UDP-N-acetylglucosamine 2-epimerase/N-acetylmannosamine k	7		
	UFL1	O94874	E3 UFM1-protein ligase 1 (EC 6.3.2.-) (LZAP-binding protein)	3		
	CNN2	B4DDF4	Calponin-2 (cDNA FLJ52469, highly similar to Calponin-2)	10		
	TCIRG1	Q13488	V-type proton ATPase 116 kDa subunit a isoform 3 (V-ATPase 116 kDa iso	2		
	TTI1	O43156	TELO2-interacting protein 1 homolog (Protein SMG10)	8		
	KIF11	P52732	Kinesin-like protein KIF11 (Kinesin-like protein 1) (Kinesin-like spin	2		
	B2RCP7_HUMAN	B2RCP7	cDNA, FLJ96197, highly similar to Homo sapiens connective tissue growt	3		Y
	KIAA2013	Q8IYS2	Uncharacterized protein KIAA2013	3		
	IQGAP1	P46940	Ras GTPase-activating-like protein IQGAP1	418		

Other related components	STAT1	P42224	Signal transducer and activator of transcription 1-alpha/beta	139		
	MX1	P20591	Interferon-induced GTP-binding protein Mx1	84		
	CLIC4	Q9Y696	Chloride intracellular channel protein 4	45		
	IFIT1	P09914	Interferon-induced protein with tetratricopeptide repeats 1	36		Y
	WARS	P23381	Tryptophan--tRNA ligase, cytoplasmic	32		
	MX2	P20592	Interferon-induced GTP-binding protein Mx2	30		Y
	OAS2	P29728	2'-5'-oligoadenylate synthase 2 ((2-5')oligo(A) synthase 2)	13		
